# Diversity-Oriented Synthesis Catalyzed by Diethylaminosulfur-Trifluoride—Preparation of New Antitumor Ecdysteroid Derivatives

**DOI:** 10.3390/ijms23073447

**Published:** 2022-03-22

**Authors:** Máté Vágvölgyi, Endre Kocsis, Márta Nové, Nikoletta Szemerédi, Gabriella Spengler, Zoltán Kele, Róbert Berkecz, Tamás Gáti, Gábor Tóth, Attila Hunyadi

**Affiliations:** 1 Institute of Pharmacognosy, Interdisciplinary Excellence Centre, University of Szeged, H-6720 Szeged, Hungary; vagvolgyi.mate@pharmacognosy.hu (M.V.); endrukocsis2@gmail.com (E.K.); 2 Department of Medical Microbiology, Albert Szent-Györgyi Health Center and Albert Szent-Györgyi Medical School, University of Szeged, H-6725 Szeged, Hungary; nove.marta@gmail.com (M.N.); szemeredi.nikoletta@med.u-szeged.hu (N.S.); spengler.gabriella@med.u-szeged.hu (G.S.); 3 Department of Medical Chemistry, University of Szeged, H-6720 Szeged, Hungary; kele.zoltan@med.u-szeged.hu; 4 Institute of Pharmaceutical Analysis, University of Szeged, H-6720 Szeged, Hungary; berkecz.robert@szte.hu; 5 Servier Research Institute of Medicinal Chemistry (SRIMC), H-1031 Budapest, Hungary; tamas.gati@hu.netgrs.com; 6 NMR Group, Department of Inorganic and Analytical Chemistry, Budapest University of Technology and Economics, H-1111 Budapest, Hungary; 7 Interdisciplinary Centre of Natural Products, University of Szeged, H-6720 Szeged, Hungary

**Keywords:** DAST, semi-synthesis, fluorination, Beckmann-rearrangement, cyclopropane, natural product, ecdysteroid, NMR, structure elucidation, anticancer

## Abstract

Fluorine represents a privileged building block in pharmaceutical chemistry. Diethylaminosulfur-trifluoride (DAST) is a reagent commonly used for replacement of alcoholic hydroxyl groups with fluorine and is also known to catalyze water elimination and cyclic Beckmann-rearrangement type reactions. In this work we aimed to use DAST for diversity-oriented semisynthetic transformation of natural products bearing multiple hydroxyl groups to prepare new bioactive compounds. Four ecdysteroids, including a new constituent of *Cyanotis arachnoidea*, were selected as starting materials for DAST-catalyzed transformations. The newly prepared compounds represented combinations of various structural changes DAST was known to catalyze, and a unique cyclopropane ring closure that was found for the first time. Several compounds demonstrated in vitro antitumor properties. A new 17-*N*-acetylecdysteroid (**13**) exerted potent antiproliferative activity and no cytotoxicity on drug susceptible and multi-drug resistant mouse T-cell lymphoma cells. Further, compound **13** acted in significant synergism with doxorubicin without detectable direct ABCB1 inhibition. Our results demonstrate that DAST is a versatile tool for diversity-oriented synthesis to expand chemical space towards new bioactive compounds.

## 1. Introduction

Due to its small size and high electronegativity, fluorine has become a building block of major importance for medicinal chemistry [1]. Fluorine may serve as a bioisostere and functional mimetic of a wide range of functional groups, while its unique properties confer fluorine substituted compounds higher lipophilicity and a typically greater metabolic stability than their non-fluorine containing counterparts [2]. Its importance in drug design is well illustrated by the fact that the annual contribution of organofluorine compounds to the FDA-approved small-molecule drugs has reached ca. 40–50% during the last few years [3]. Further, fluorination is also a useful labelling tool to improve bioanalytical sensitivity [4,5].

Diethylaminosulfur trifluoride (DAST) is a mild, nucleophilic reagent that may convert non-phenolic alcohols, aldehydes and ketones, carboxylic acids, and sulfoxides into monofluorides, difluorides, acyl fluorides, and α-fluoro sulfides, respectively. Other than fluorination, however, DAST is known to catalyze the formation of many further building blocks valuable for medicinal chemistry, including various heterocycles through dehydration and intramolecular cyclization [6], sulfonamides through cross-coupling of arylboronic acids [7], aromatic thiols [8], etc. Previously, we reported the DAST catalyzed transformation of 20-hydroxyecdysone 2,3;20,22-diacetonide, and obtained 14- and/or 25-fluorinated and Δ^14,15^ analogs with or without a 25-fluorine moiety [9]. Several compounds showed stronger antiproliferative activity on various cancer cell lines than their parent ecdysteroid, and promising adjuvant antitumor properties when co-administered with doxorubicin. Further, all the compounds showed an increased potency as inhibitors of the ABCB1 transporter, commonly referred to as P-glycoprotein (Pgp) [9]. In contrast with the diacetonide, we found the sidechain cleaved ecdysteroid derivative poststerone to form cyclic sulfite esters that were only moderately active against some cancer cell lines [10].

From our previous extensive structure-activity relationship studies on the antitumor properties of ecdysteroids we found that their multidrug resistance decreasing activity does not rely on Pgp inhibition [11,12,13,14]. Because of this, our research on new antitumor ecdysteroid derivatives focuses on compounds that exert their activity without directly affecting drug efflux, and, as such, are free from the many potential problems (e.g., unwanted drug-drug interactions, altered pharmacokinetics of co-administered antitumor drugs, etc.) frequently attributed to efflux pump inhibitors. As a follow-up to our previous work, in the current study it was our aim to (i) explore the use of DAST as a chemical tool for diversity-oriented semi-synthesis, and in doing so, to (ii) prepare new ecdysteroid derivatives with adjuvant antitumor effect against MDR cancer.

## 2. Results and Discussion

### 2.1. Chemistry

#### 2.1.1. Preparation of Starting Materials for Reactions with DAST

**Figure 1:**
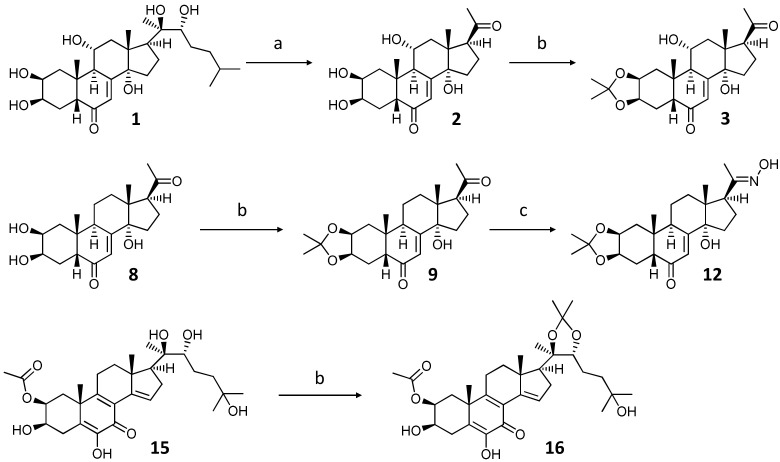
Semi-synthetic transformations of ajugasterone C (**1**), poststerone (**8**), and calonysterone 2-acetate (**15**). Reagents and conditions: (**a**) PIDA, methanol, RT, 60 min; (**b**) PMA, acetone, RT, 30 min; (**c**) NH_2_OH·HCl, KOH, EtOH, RT, 24 h.

We selected four apolar ecdysteroids including three sidechain-cleaved (**3**, **9**, **12**) and a sidechain intact compound (**16**) as substrates for diethylaminusulfur-trifluoride (DAST)-mediated transformations. Each of these compounds represent a derivative of a natural ecdysteroid (compounds **1**, **8**, and **15**, respectively) used as a precursor in the semi-synthetic processes that afforded the selected intermediates for further transformations (Figure 1).

Selection of these compounds was based on our previous findings. These may be summed up in two key points: (i) the adjuvant antitumor activity of ecdysteroids requires the presence of apolar functional groups, e.g., acetonide, particularly at the A-ring, and (ii) removal of the sterol side-chain results in the loss of the compounds’ direct Pgp inhibitory activity [11,12].

Oxidative side-chain cleavage of ajugasterone C (**1**) was achieved in good yield following our previously published procedure using hypervalent iodine reagent (diacetoxyiodo)benzene (PIDA) in methanol that afforded 11*α*-hydroxypoststerone (**2**), which was subsequently converted to its 2,3-acetonide derivative (**3**) using phosphomolybdic acid (PMA) in acetone [11].

To increase the diversity of interesting, potentially bioactive substrates for our fluorination reactions, we selected another sidechain cleaved ecdysteroid, poststerone (**8**), for transformation. Poststerone is known as a natural metabolite of 20-hydroxyecdysone, the most abundant ecdysteroid existing in nature, and thus, it can be straightforwardly obtained in larger quantities from the oxidative side-chain cleavage of the parent compound [11]. To facilitate antitumor properties, poststerone (**8**) was also further converted to its corresponding 2,3-acetonide derivative (**9**).

Oximes and oxime ethers are valuable precursors in the preparation of bioactive nitrogen-containing scaffolds [15,16]. As a follow-up to our previous work with nitrogen-containing ecdysteroids [13], we transformed poststerone 2,3-acetonide (**9**) to its 20-oxime derivative by reacting the substrate with hydroxylamine in an ethanol solution. As an update to our former semi-synthetic strategy [17], the regioselectivity of the oximation can be significantly improved by changing the solvent from pyridine to ethanol that can afford the desired 20-acetoxime product in >80% yield, under simplified workup conditions.

Calonysterone 2-acetate (**15**) is a natural analogue of its parent compound calonysterone. This ecdysteroid was recently revealed in an in silico screening as a putative inhibitor of papain-like protease (PL^pro^), a major druggable target in SARS-CoV-2 treatment, affording potential anti-COVID-19 properties to the compound [18]. To increase potential regioselectivity in a subsequent DAST-catalyzed transformation, we carried out the acetonide protection of the molecule’s 20,22-diols. Preparation of the above-described intermediates is shown in Figure 1.

#### 2.1.2. DAST-Mediated Transformation of Compounds **3**, **9**, **12**, **16**, **18**, and Structure Elucidation of the Products

Reactions were carried out according to our previously reported procedure [9]. DAST is known to react violently with water [19]; thus, substrates were dissolved in anhydrous methylene-chloride, and the obtained solutions were cooled down to −84 °C to avoid any undesired exothermic side-reactions. When the transformations were complete, the products were purified via single- or multi-step preparative HPLC separations. Following this strategy, we successfully obtained a total of nine new ecdysteroid derivatives.

The NMR signals of the products were assigned by comprehensive one- and two-dimensional NMR methods using widely accepted strategies [20,21]. Most ^1^H assignments were accomplished using general knowledge of chemical shift dispersion with the aid of the proton–proton coupling pattern (^1^H NMR spectra). ^1^H NMR chemical shifts of overlapped signals were identified by 2D HSQC and HMBC experiments, and by utilizing 1D selective ROESY (Rotating frame Overhauser Enchancement Spectroscopy) or NOESY responses and 1D selective TOCSY experiments. ^1^H and ^13^C NMR signal assignments for compounds **4**–**7**, **10**, **11**, **13**, **14**, **15**, **17**, and **19** are compiled in Table 1 and Table 2.

**Figure 2:**
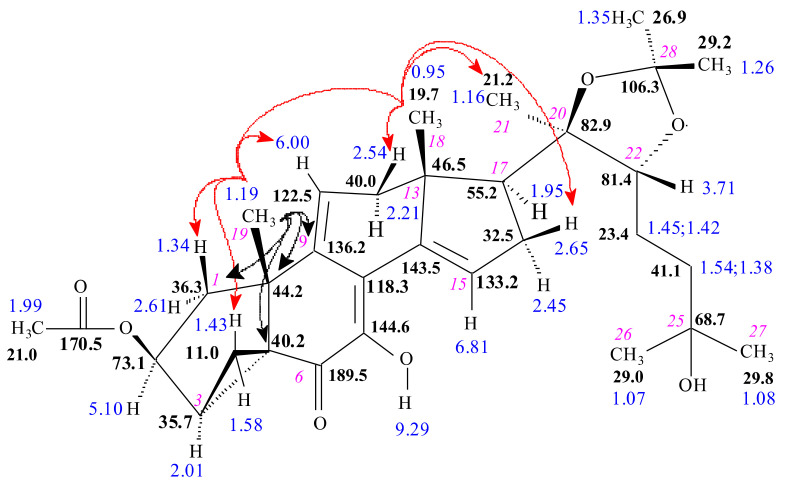
Stereostructure of compound **3** (C_24_H_34_O_6_) along with its ^1^H (blue numbers) and ^13^C NMR (black numbers in bold) signal assignments. Cursive numbers give the atomic numbering. The α or β orientation of hydrogen atoms was determined by selROE measurements starting from Hα-2 (4.53 ppm), H_3_-18 (0.62 ppm), and H_3_-19 (1.02 ppm), and red arrows show spatial proximities proven this way.

To facilitate understanding of the structure elucidation steps, we prepared the yet unpublished complete ^1^H and ^13^C NMR signal assignments of the intermediate compound **3** (Figure 2), aided by its ^1^H, DEPTQ, edHSQC, and HMBC spectra. In the following, structure elucidation of the sidechain shortened derivatives (compounds **4**–**7**, **10**, **11**, **13**, and **14**) is described in comparison with these results.

For compound **4**, HRMS data indicated an elemental composition of C_24_H_32_O_5_ (Supporting Material Figure S56), the molecule consists of one oxygen and two hydrogen atoms less than its parent compound **3**, and the number of its double bond equivalents increased to 9. The most characteristic changes in the NMR spectra, as compared with that of **3**, indicated the disappearance of the HO–CH–CH_2_ group in the B ring (δC-11 67.9, δH-11 4.25; δC-12 41.0, δH_2_-12 2.32, 2.18 ppm) and presence of a new –CH=CH– group (δC-11 124.5, δH-11 5.78, *J*_11,12_ = 10 Hz; δC-12 133.7, δH-12 6.33 *J*_11,12_ = 10 Hz). To achieve the complete ^1^H and ^13^C NMR signal assignment in CDCl_3_, ^1^H, and selTOCSY on H-5, H-12, and H-7 spectra (Figure S1), selNOESY on δMe, H_3_-19, and H_3_-18 (Figure S2); DEPTQ (Figure S3); edHSQC (Figure S4); and HMBC (Figure S5) spectra were utilized. SelNOESY experiments on H_3_-18 and H_3_-19 (Figure S2) unambiguously differentiated H-11 and H-12 signals; furthermore, the 0.62/41.0 ppm H_3_-19/C-12 HMBC cross-peak (Figure S5) confirmed the Δ^11,12^ position of the new double bond. The chemical shifts of all other ^1^H and ^13^C NMR signals in compound **4** are similar to that of **3**.

According to the HRMS measurement, the molecular formula of compound **5** is identical to that of compound **4** C_24_H_32_O_5_ (Figure S57) and the number of its double bond equivalents is also nine; both compounds are structural isomers. The ^1^H and DEPTQ spectra of 5 revealed in this compound the appearance of one tri-substituted C=CH− double bond (δC-9 135.7; δC-11 130.8, δH-11 6.17 ppm). The following spectra were used for NMR study of **5**: ^1^H and selTOCSY on H-7 spectra (Figure S6), selNOESY on αMe, H_3_-19, and H_3_-18 (Figure S7), DEPTQ (Figure S8), edHSQC (Figure S9) and HMBC (Figure S10) spectra were utilized. The 1.23/135.7 ppm H_3_-19/C-9 HMBC response (Figure S9) revealed the C-9 position of the quaternary C= atom, i.e., the Δ^9,11^ position of the new double bond. In addition, the 0.64/36.0 ppm H_3_-18/C-12 HMBC cross-peak confirmed the existence of H_2_C group in this position. All other ^1^H and ^13^C NMR signals in **5** are highly similar to that of **4**.

The molecular formula of compound **6** was established as C_24_H_30_O_4_ by means of HRMS (Figure S58), i.e., the number of double bond equivalents rose to 10. The ^1^H (Figure S11) and DEPTQ (Figure S13) spectra of **6** confirmed in this compound the presence of two tri-substituted C=CH− double bonds (δC-9 135.9; δC-11 130.1, δH-11 6.17 ppm) and (δC-14 142.9; δC-15 128.4, δH-15 6.28 ppm). The Δ^9,11^ position is supported by the 1.14/135.9 ppm H_3_-19/C-9 HMBC response, whereas the 0.84/142.9 ppm H_3_-18/C-14 HMBC cross-peak confirmed the Δ^14,15^ arrangement (Figure S15) of the other double bond, respectively, formed by the elimination of the 14-OH group. The edHSQC (Figure S14) experiment served the selective identification of CH_2_ and CH/CH_3_ ^1^H and ^13^C signals, and was supplemented by the results of the selNOESY measurements on αMe, H_3_-19, and H_3_-18 (Figure S2); therefore, even the stereochemical assignment of ^1^H signals has been achieved.

The reaction of **3** with DAST also resulted in compound **7**, and its elemental composition was established as C_24_H_31_O_4_F (Figure S59), and the number of double bond equivalents is 9. The ^1^H (Figure S16) and DEPTQ (Figure S16) spectra and the 1.20/135.7 ppm H_3_-19/C-9 HMBC response (Figure S19) revealed the Δ^9,11^ position of the new tri-substituted C=CH− double bond (δC-9 135.7; δC-11 130.6, δH-11 6.18 ppm), which was produced by the splitting of the 11-OH group. Previously, we reported the NMR data of 14-fluorinated ecdysteroid derivatives obtained by DAST catalyzed transformation [9]. It was found that changes from an HO-14 group to a F-14 manifest in a ~25 ppm paramagnetic shift on δC-14, and at the same time in dublet multiplicity of the signal, caused by ^1^*J*_C,F_ ~ 165 Hz coupling. It is worth noting that the geminal, vicinal, and ^n^*J*_C,F_ couplings result characteristic ~25 Hz, ~10 Hz, and ~3 Hz signal splittings, respectively. The 0.67/106.0 ppm H_3_-18/C-14 HMBC cross-peak (Figure S19) and its doublet multiplicity (^1^*J*_C,F_ ~ 168 Hz) clearly justified the fluorination in C-14 position. The edHSQC (Figure S18) spectrum served the selective identification of CH_2_ and CH/CH_3_ ^1^H and ^13^C signals. It is important to mention that the selROESY measurements on H_3_-19 and H_3_-18 (Figure S16) revealed not only the α or β position of the hydrogen atoms, but clearly demonstrated the *trans* C/D ring-junction and thus the α position of the 14-fluorine atom.

Recently we reported the NMR characteristics of posterone and a series of posterone 2,3-dioxalanes, including compound **9** [11]. The DAST-catalyzed transformation of posterone 2,3-acetonide afforded compound **10**. Its HRMS data indicated an elemental composition of C_24_H_32_O_4_ (Figure S60): the molecule consists of one oxygen and two hydrogen atoms less than its parental **9**, and the number of its double bond equivalents rose to 9. These suggested that water elimination took place. This was confirmed by the ^1^H (Figure S20), DEPTQ (Figure S22), and edHSQC (Figure S23) spectra indicating the presence of a new tri-substituted C=CH− double bond (δC-14 146.1; δC-15 128.2, δH-15 5.97 ppm). The 0.85/146.1 ppm H_3_-18/C-14 HMBC cross-peak confirmed the Δ^14,15^ arrangement (Figures S24 and S25). The selTOCSY experiment on H-15 and Hα-1 (Figure S20) served the separate recognition of the ^1^H signals in the A and D rings, whereas selNOESY on signals βMe, H_3_-19, and H_3_-18 (Figure S21) supported the α or β assignment of hydrogen atoms. 

Compound **11** was obtained also from DAST-catalyzed transformation of **9**. The elemental composition was C_24_H_33_O4_F_ (Figure S60), obtained by HRMS, and the number of double bond equivalents was 8. These results suggested that the HO- group was exchanged to –F atom. This was completely confirmed by the NMR data (Figures S26–S31). The 0.65/107.3 ppm H_3_-18/C-14 HMBC cross-peak (Figures S30 and S31) and its doublet multiplicity (^1^*J*_C,F_ = 166 Hz) perfectly justified the fluorination in C-14 position. The selNOESY experiments on signals βMe, H_3_-19, and H_3_-18 revealed the stereochemistry of hydrogen atoms, and perfectly supported *trans* C/D ring-junction and in this way the Fα–14 substitution. 

Based on HRMS data, an elemental composition of C_24_H_33_O_4_N (Figure S60) was established for compound **13**. All of its ^1^H and ^13^C spectra—^1^H and selTOCSY on H-17 and H-3 spectra (Figure S32), selROESY on H_3_-19 and H_3_-18 (Figure S33), DEPTQ (Figure S34), edHSQC (Figures S35 and S37), and HMBC (Figures S36 and S37) spectra—are quite similar to spectra measured for compound **10**. Characteristic changes were detected only for the CH_3_−C=O (2.02 s; 23.4, and 170.2 ppm) group, and appeared a new δNH signal (5.80 d *J*_H-17,NH_ = 9.0 Hz). The measured 37.6 ppm diamagnetic shift of the C=O signal in relation to compound **10**, is in accord with one amide group. The 0.67/106.0 ppm H_3_-18/C-14 HMBC cross-peak (Figure S19) and its doublet multiplicity (^1^*J*_C,F_ ~ 168 Hz) clearly justified the fluorination in C-14 position.

For compound **14**, HRMS data indicated an elemental composition of C_24_H_34_O_4_NF (Supporting Material Figure S56), the molecule consists of one fluorine and one hydrogen atom more than in the former compound **13**, and the number of its double bond equivalents decreased to 8. Comparing the ^1^H (Figure S38), DEPTQ (Figure S41) and edHSQC (Figure S42) spectra with the fairly similar spectra of compound **13**, the presence of the HN–CO–CH_3_ (δNH 5.57, 170.1 and 2.02 s; 23.6 ppm) amide group in position 17 is straightforward. The disappearance of the signals for the Δ^14,15^ moiety (141.6 and 128.2; 5.97 ppm) and the appearance of characteristic (^1^*J*_C,F_ = 165 Hz) signal splitting at 105.5 d ppm revealed the fluorination. The 0.71/105.5 ppm H_3_-18/C-14 HMBC correlation (Figure S44) confirmed the fluorination in C-14 position. The selNOESY experiments on signals NH, H_3_-19, and H_3_-18 (Figure S40) revealed the characteristic hydrogen/hydrogen steric proximities, and perfectly supported trans C/D ring-junction and in this way the 14α-F substitution. Despite of strong ^1^H signal overlaps the separate identification of spin-systems of A, C, and D rings was successfully implemented by selTOCSY on H-3, H-9, and H-17 (Figure S39). The edHSQC spectrum (Figure S43) with insertion of the one-dimensional selTOCSY on H-17 spectrum (Figure S39) proved to be very effective in assignment of close and broad signals.

The natural product calonysterone 2-acetate **15** served as the starting material for further synthesis; for reference, its ^1^H and ^13^C NMR data are inserted to Table 2.

**Figure 3:**
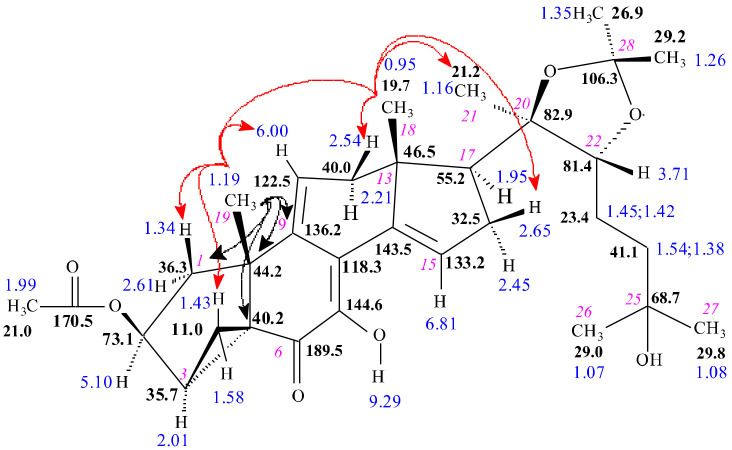
Stereostructure of compound **17** along with ^1^H (blue) and ^13^C NMR (black) chemical shifts. Cursive numbers denote the atomic numbering. The red arrows show spatial proximities determined by selNOE measurements and black arrows indicate characteristic HMBC responses.

The reaction of calonysterone 2-acetate 20,22-acetonide (**16**) with DAST resulted in **17**. HRMS revealed an elemental composition of C_32_H_44_O_7_ (Figure S64) for this compound, suggesting that water elimination took place. The number of double bond equivalents increased to 11; therefore, this compound contains five double bonds and six rings. The presence of seven methyl signals in the ^1^ H (Figure S45) and DEPTQ (Figure S47) spectra suggested that the sterol side chain at C-17 and the 2-acetate group remained intact. Appearance of the characteristic signals of the Δ^14,15^ moiety (δC-14 145.3; δC-15 133.2, δH-15 6.81 ppm) revealed the unchanged steroid D-ring. Based on the HSQC (Figure S48) experiment, the signals of the H-C-3 group (2.01; 35.7 ppm) showed an extra high diamagnetic shift. This means that elimination of the 3-OH group took place. The selTOCSY experiment on H-2 (Figure S46) also identified the δH_2_-4 signals (1.42 and 1.58 ppm). The extreme high deshielding on the corresponding δC-4 (11.0 ppm) (Figure S48) clearly indicated the formation of a cyclopropane ring between C-3 and C-5, and thus the existence of a five-membered A-ring. At the same time the quaternary C-5 atom changed to an sp^3^ carbon and its δ40.2 ppm chemical shift is identified by the H_3_-19/C-5 cross-peak in the HMBC spectrum (Figure S48). The H_3_-19/C-9 (1.19/136.22 ppm) HMBC correlation revealed a Δ^9,11^ double bond in the B ring. SelNOESY experiments on H_3_-18 and H_3_-19 (Figure S46) unambiguously differentiated α/β positions of hydrogen atoms and differentiated between the methylene hydrogens in the cyclopropane ring. The significant changes in the NMR chemical shifts in the B and C rings can be well explained with the rearrangement of double bonds of the 6-hydroxy-Δ^5,6^-7-one-Δ^8,9^ chromophore of calonysterone. This is in agreement with our previous report on the NMR characteristics of calonysterone and isocalonysterone [22]. The revealed stereostructure of compound 17 along with characteristic NOE and HMBC correlations is shown in Figure 3.

**Figure 4:**
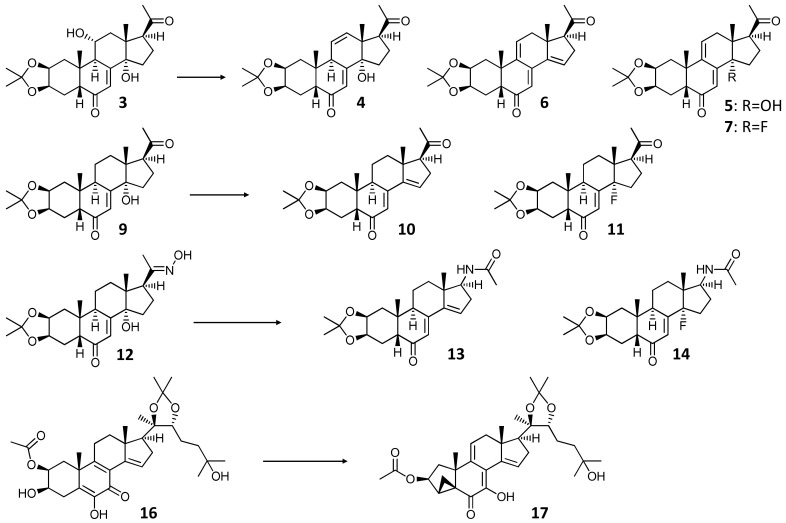
Structures of products obtained by the DAST-mediated transformation of compounds **3**, **9**, **12**, and **16**. Reagents and conditions: DAST, anh. CH_2_Cl_2_, −84 °C → room temperature, 70 min.

The reactions carried out with DAST, and structures of the compounds obtained are presented in Figure 4.

In general, the DAST-mediated transformation of ecdysteroids afforded structurally diverse products including ecdysteroid anhydro derivatives (e.g., compound **10**) formed by water elimination, fluorine substituted analogues (e.g., compound **11**), compounds with both newly formed olefins and fluoride groups (e.g., compound **7**), two new acetamides (**13** and **14**), and a unique new ecdysteroid with a cyclopropane ring (**17**).

The ability of DAST to promote the skeletal rearrangement of oximes to substituted amides (known as Beckmann-rearrangement) was previously reported [23]. Additionally, DAST was recently found to be a useful reagent to induce the Beckmann-fragmentation of *α*-oximinoketones resulting in the preparation of aryloyl and aliphatic acyl fluorides [24]. In accordance with the previous reports, the reaction of ecdysteroid 20-acetoximes with DAST could effectively furnish the corresponding amides and resulted in two different Beckmann products, whereas the single available hydroxyl group at the 14*α* position was either eliminated (**13**) or substituted with fluorine (**14**).

**Figure 5:**
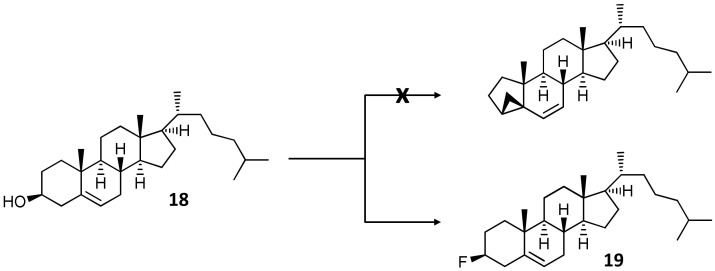
DAST-mediated fluorination of cholesterol (**18**). Reagents and conditions: DAST, anh. CH_2_Cl_2_, −84 °C → room temperature, 70 min.

The reaction of calonysterone 2-acetate 20,22-acetonide (**16**) with DAST resulted in a highly complex, multi-component mixture of products that had been subjected to a multistep preparative HPLC separation. This led to the isolation of one single product, compound **17**, in a very low yield. The subsequent structure elucidation of this compound revealed an unexpected cyclopropane ring closure between C-3 and C-5, providing a unique, five-membered A-ring steroid skeleton to the product. DAST is known to form a carbocation by water elimination. Our hypothesis is that this intermediate could react with the Δ^5,6^-olefin of the substrate, inducing ring closure and the subsequent rearrangement of the conjugated double bonds of rings B and C. The stereoselectivity of the reaction should be due to the rigid steroid skeleton. To further elaborate this notion, cholesterol (**18**) was selected as a structurally related, commercially available model compound for a DAST-mediated transformation under the same synthetic conditions as before. This reaction selectively yielded **19.** This compound showed ^1^H (Figure S50) and APT (Attached Proton Test, Figure S52) spectra very similar to those of **18**. Noticeable differences were identified only for the A-ring signals, mainly around C-3. The nearly ~25 ppm paramagnetic shift of δC-3 at 92.8 d ppm and its characteristic (^1^*J*_C,F_ = 174 Hz) signal splitting proved C-3 fluorination. To analyze the broad multiplet Hα-3 signal at 4.39 ppm, the H_2_-4 hydrogen atoms were decoupled, and thus the simplified multiplicity allowed reading of the couplings of Hα-3 (^2^*J*_H,F_ = 51 Hz; *J*_2α,3α_ = 4.8 Hz and *J*_2β,3α_ = 11.1 Hz). Due to the rather crowded feature of the ^1^H spectrum, the ^1^H,^1^H-COSY (Figure S51) could be used only for separated signals, but the selROE experiment (Figure S51) on signal 4.39 ppm marked out the Hα-2 and Hα-1 signals (1.99 and 1.07 ppm, respectively). To overcome difficulties caused by the moderate resolution of the HSQC experiment (Figure S53), band-selective HSQC method was used to unambiguously assign the neighboring signals in the ranges of 36–37 and 39–40 ppm. selROE experiment was used in combination with edited HSQC to elucidate stereochemistry of hydrogen atoms (Figure S54), and the HMBC experiment (Figure S55), especially correlations of hydrogens of 18, 19, 21, 26, and 27 methyl groups supported the assignments. The combination of all these experiments allowed a complete ^1^H and ^13^ C assignment for compound **19**. The result of this transformation is shown in Figure 5.

In contrast to the case of calonysterone 2-acetate 20,22-acetonide (**17**), the reaction of cholesterol (**18**) with DAST selectively yielded the corresponding 3-fluorine analogue (**19**) of the substrate, and no traces of the sought cyclopropane ring-closed derivative were observed in the product mixture. Nevertheless, the retained β-orientation of the fluorine substituent confirms the S_N_1 reaction mechanism and therefore the involvement of a carbocation intermediate. This supports the mechanism proposed for the ring closure to form compound **17**, while a more extended conjugation in the B-ring, such as the *o*-quinol dienone moiety of calonysterone, may be a prerequisite for the rearrangement to a cyclopropane ring.

### 2.2. Biology

#### 2.2.1. Cytotoxicity, Anti-Proliferative, and ABCB1 Inhibitory Activities

Selected compounds, all possessing a purity of over 95% by means of HPLC (see Supplementary Materials, Figures S65–S76), were tested on two mouse lymphoma cell lines: L5178Y, and its transfected multi-drug resistant counterpart, LT5178Y_MDR_ that expresses a major human MDR efflux transporter, ABCB1. The compounds were also tested on MRC-5 normal human fibroblasts. Results are summarized in Table 3.

To assess the compounds’ effect on cell growth and viability, we used two different experimental setups of MTT assay. This allowed a comparative evaluation of results obtained from a short-term (24 h) treatment of a relatively higher number (10,000) of cells and those from a long-term (72 h) treatment of a lower cell number (6000), which makes it possible to gain some insight into the cytotoxic vs. cytostatic properties of the compounds. Even though MTT assay is an endpoint detection method, i.e., it provides a single readout of viable cells at the end of the experiment, such a comparison offers a reliable tool to identify antiproliferative activity of compounds that are not cytotoxic.

In general, the cell viability data indicated that all sidechain cleaved compounds exerted very weak cytotoxicity on each cell line, while their cytostatic, antiproliferative activity was several times higher. This was particularly true for the 17-*N*-acetyl derivative **13** whose antiproliferative activity on the lymphoma cell lines (IC_50_ ca. 4.7 µM) was by a remarkable over 20 times stronger than its cytotoxicity (IC_50_ > 100 µM). On the other hand, compound **17** that contains an intact sidechain was the most cytotoxic among the tested ecdysteroids, and its cytotoxicity was also in a similar dose range as its antiproliferative activity. Further, **17** was also the only compound that showed a significant, ca. 50% direct ABCB1 inhibition. In line with our previous results [11], all sidechain shortened derivatives were inactive in this regard. Further structure-activity relationships may also be concluded concerning the steroid core. When comparing the antiproliferative activities of compounds **10**, **11**, **13**, and **14**, it appears that a 17-*N*-acetyl group is favorable over the 17-acetyl, and that a Δ^14,15^ olefin is favorable over a 14α-F group. Nevertheless, it is also clear that the effect of a 14-fluorine group on the antitumor activity of ecdysteroids depends on its chemical environment. In case of compounds **5**, **6**, and **7** that also contain a Δ^6(9,11)^ conjugated olefin, the antiproliferative activity increases in the 14-OH < Δ^14,15^ < 14-F order. This is well in agreement with our previous findings on the fluorination products of 20-hydroxyecdysone 2,3;20,22-diacetonide [9].

Compounds **6**–**10**, **14**, and **16** also exerted tumor selective activity in both experimental setups, and compound **13** demonstrated a good, ca. 2.5 times selective antiproliferative activity against both tumor cell lines.

Concerning the plausible mechanism of action for our ecdysteroid derivatives, it is of interest that 20-hydroxyecdysone has most recently been identified as a mitochondrial assembly receptor (MasR) agonist, i.e., mimicking the action of angiotensin-(1-7) ((Ang-(1-7)) [25]. Activation of the ACE2/Angiotensin-(1-7)/MasR axis points towards suppressing cancer proliferation, angiogenesis, and metastasis [26], and Ang-(1-7) itself acts as such on various types of cancer [27]. It is a tempting hypothesis that the potent antiproliferative effect of our ecdysteroids might be due to their action through this signaling pathway. Further studies are needed to elaborate on this.

#### 2.2.2. Combination Assays

Compounds **7**, **10**, **11**, **13**, and **14** had sufficient individual antiproliferative properties for their assessment of chemo-sensitizing activity. This was carried out as an antiproliferative assay on L5178Y_MDR_ cells in combination with doxorubicin, arranged according to the checkerboard microplate method [28]. Results are shown in Table 4.

Most of the compounds showed nearly additive to mild synergistic effects with doxorubicin. The only exception was the 17-*N*-acetyl derivative (**13**) that showed a relevant synergism with doxorubicin already at 50% inhibition. This, taken together with the potent cytostatic effect of compound **13**, makes it a valuable candidate for further studies toward a chemosensitizer for combination therapy with the cytotoxic drug doxorubicin. It is worth stressing here that we consider it as an advantage that compound **13** is inactive as an efflux pump inhibitor, and therefore its adjuvant antitumor action is free from the potential complications associated with Pgp inhibitors [29]. Nevertheless, the mechanism of action behind this phenomenon is unclear, and currently it is also not known whether a possible MasR agonist activity of our compounds could be associated with their MDR decreasing properties.

## 3. Materials and Methods

### 3.1. Synthesis and Chromatographic Purification

Solvents and reagents were purchased from Sigma (Merck KGaA, Darmstadt, Germany), and were used without any further purification. The progress of the reactions was monitored by thin layer chromatography on Kieselgel 60F_254_ silica plates purchased from Merck (Merck KGaA, Darmstadt, Germany), and characteristic spots of compounds were examined under UV illumination at 254 and 366 nm. Chromatographic purification of the semi-synthesized steroid derivatives was carried out in one or two steps, depending on the complexity of their product mixtures (see Table 5). For flash chromatography, a CombiFlash^®^ Rf+ Lumen instrument (TELEDYNE Isco, Lincoln, NE, USA) was used that was equipped with ELS and diode array detectors. The crude product mixtures were separated on RediSep NP-silica flash columns (TELEDYNE Isco, Lincoln, NE, USA) purchased from commercial source. Purity analysis of products was performed via HPLC on normal- or reverse phase 5 µm, 250 × 4.6 mm, 100 Å, Phenomenex columns (Phenomenex Inc., Torrance, CA, USA) at 1 mL/min. flow rate, while using a dual pump (PU-2080) Jasco HPLC instrument (Jasco International Co. Ltd., Hachioji, Tokyo, Japan) that was equipped with an MD-2010 Plus PDA detector to collect data in a range of 210–400 nm. Preparative HPLC separations were carried out on an Armen Spot Prep II integrated HPLC purification system (Gilson, Middleton, WI, USA) with dual-wavelength detection applied.

### 3.2. Preparation of Ecdysteroid Derivatives

#### 3.2.1. Natural Ecdysteroids Isolated from Plants or Semi-Synthesized before

Ajugasterone C (**1**) was isolated during our previous phytochemical work [30]. Calonysterone 2-acetate (**15**) was isolated from a fraction of a commercial extract of *Cyanotis arachnoidea* C.B. Clarke (Shaanxi KingSci Biotechnology Co., Ltd., Xi’an, People’s Republic of China) during the current study and is reported here as a new ecdysteroid. We have recently reported the purification process in detail. Briefly, calonysterone 2-acetate was obtained after the following fractionation steps: percolation with methanol, column chromatography on silica (Merck KGaA, Darmstadt, Germany; eluent: dichloromethane—methanol/95:5, *v*/*v*), column chromatography on Lichroprep RP C18 (Merck KGaA, Darmstadt, Germany; eluent: 20% aqueous acetonitrile), and column chromatography on silica 60 GF_254_ (Merck KGaA, Darmstadt, Germany; eluent: *n*-hexane—ethyl acetate 6:5, *v*/*v*). For ^1^H and ^13^C NMR chemical shifts of compound **15** see Table 2.

Poststerone was previously prepared by semi-synthesis from 20-hydroxyecdysone isolated from the same extract of *C. arachnoidea* [11].

#### 3.2.2. Preparation of Sidechain Cleaved Ajugasterone C Derivative (2)

An aliquot of 2 g of ajugasterone C (**1**) (4.16 mmol) was dissolved in 160 mL of methanol and 1.2 equiv. (2.15 g; 4.99 mmol) of PIDA was added to the solution. The reaction mixture was stirred at room temperature for 60 min and subsequently neutralized by 5% aq. NaHCO_3_-solution. After evaporation under reduced pressure, the product’s dry residue was redissolved in methanol, and silica gel (~10 g) was added to the solution. Following this, the solvent was evaporated to prepare the sample for dry loading flash chromatographic separation (see Table 5) to obtain 11*α*-hydroxypoststerone (**2**) (1.11 g, 70.2%).

#### 3.2.3. General Procedure for the Preparation of Ecdysteroid Acetonides **3**, **9**, **16**

Compounds **2**, **8**, and **15** were each dissolved in acetone in 1 g/100 mL concentration. To these solutions, 1 g of phosphomolybdic acid for each gram of starting material was added. The mixtures were sonicated at room temperature for 30 min. Then, the reaction mixtures were neutralized with 10% aq. NaHCO_3_-solution, which was followed by the evaporation of acetone under reduced pressure on a rotary evaporator. Compounds were extracted from their aqueous residue with 3 × 50 mL of dichloromethane, and the combined organic fractions were dried over Na_2_SO_4_. After filtration, the products’ solutions were evaporated to dryness on a rotary evaporator. Compounds **3**, **9**, and **16** were obtained in their pure form after dry loading flash chromatographic separation (see Table 5), in yields of 59.8%, 55.8%, and 84.6%, respectively.

#### 3.2.4. Preparation of Poststerone 2,3-acetonide 20-oxime (**12**)

An aliquot of 240 mg of hydroxylamine hydrochloride (3.47 mmol) was dissolved in ethanol, and under stirring, 195 mg of potassium hydroxide (3.47 mmol) was added to the solution. Following this, 930 mg of poststerone 2,3-acetonide (**9**) (2.31 mmol) was added to the resulting mixture. The reaction mixture was stirred at room temperature for 24 h. Subsequently, the reaction solution was evaporated to dryness on a rotary evaporator, 50 mL of water was added to the dry residue, and extraction was performed with 3 × 50 mL of dichloromethane. The collected organic fractions were combined, dried over Na_2_SO_4_, filtered, and evaporated to dryness on a rotary evaporator. Subsequently, the dry residue was subjected to dry loading flash chromatographic purification (see Table 5), which afforded the desired 20-oxime product (**12**) in a yield of 82% (791 mg). ^1^H and ^13^C NMR chemical shifts of compound **12** were in perfect agreement with our previously published data [17].

#### 3.2.5. General Procedure for the DAST-Catalyzed Transformation of Ecdysteroids

Compounds **3**, **9**, **12**, **16**, and **18** were each dissolved in anhydrous dichloromethane in a concentration of 10 mg/mL in a round-bottom flask. The solutions were cooled down to −84 °C in an ethyl-acetate containing liquid nitrogen-cooled bath, and under stirring, 1.5 equiv. of diethylaminosulfur trifluoride (DAST) was added to them dropwise. As the reaction progressed, the mixtures were allowed to warm up to room temperature. After 70 min of stirring, the reactions were neutralized using 5% aq. NaHCO_3_-solution, and after water dilution, the compounds were extracted from their mixture with 3 × 50 mL of dichloromethane. The collected organic fractions were dried over Na_2_SO_4_, filtered, and subsequently evaporated under reduced pressure on a rotary evaporator. The purification procedures and yields of the obtained compounds **4**, **5**, **6**, **7**, **10**, **11**, **13**, **14**, **17**, and **19** are detailed in Table 5.

### 3.3. Structure Elucidation

^1^H (600 and 500 MHz) and ^13^C (150 and 125 MHz) NMR spectra were recorded at room temperature on Bruker Avance III NMR spectrometers equipped with Prodigy and CryoProbe heads, using CDCl_3_, DMSO-d_6_, or MeOH-d_4_ as solvents. Chemical shifts are given on a δ scale and referenced to the solvents (CDCl_3_ δH = 7.27 and δC = 77.0 ppm; DMSO-d_6_ δH = 2.50 and δC = 39.5 ppm; and CH_3_OH-d_4_ δH = 3.31 and δC = 49.1 ppm). Coupling constant (*J*) values are expressed in Hz. Aliquots of approximately 2–5 mg samples were dissolved in 0.1 mL of solvent and transferred to 2.5 mm Bruker MATCH NMR sample tubes or 0.6 mL to 5 mm NMR sample tubes. Pulse programs of all experiments (^1^H, ^13^C, DEPTQ, APT, sel-TOCSY, sel-ROE (τ_mix_: 300 ms), sel-NOE, edited gs-HSQC, band-selective-gs-HSQC, and gs-HMBC) were taken from the Bruker software library. ^1^H and ^13^C NMR data for the new compounds are presented in Table 1 and Table 2. Characteristic NMR spectra of these compounds, along with their stereostructures, ^1^H and ^13^C assignments, characteristic HMBC correlations, and steric proximities are presented in Figures S1–S55, Supporting Information. High-resolution mass spectra were recorded on a Q Exactive Plus Hybrid Quadrupole-Orbitrap mass spectrometer (Thermo Fisher Scientific, Waltham, MA, USA), and they are shown in Figures S56–S64.

### 3.4. Cell Lines

L5178Y mouse *T*-cell lymphoma cells (ECACC Cat. No. 87111908) were obtained from FDA, Silver Spring, MD, USA. These were transfected with pHa MDR1/A retrovirus as described previously. *ABCB1*-expressing L5178Y_MDR_ cells were selected by culturing the infected cells with colchicine. The cell lines were cultured in McCoy’s 5A medium supplemented with 10% heat inactivated horse serum, 2 mM L-glutamine, and penicillin–streptomycin mixture.

MRC-5 human embryonal lung fibroblasts (CCL-171, ATCC) were acquired from Sigma-Aldrich (Merck, KGaA, Darmstadt, Germany), and were cultured in EMEM medium containing 4.5 g/L glucose and supplemented with a non-essential amino acid mixture, a selection of vitamins, and 10% of FBS.

### 3.5. Cell Viability Assay for Determination of Cytotoxicity and Antiproliferative Activity

MTT assay was performed in 96-well flat-bottomed microtiter plates as described before. Briefly, a 10 mM concentration stock solution in DMSO was prepared for each compound. These were diluted in 100 μL of McCoy’s 5A medium. Subsequently, 1 × 10^4^ (cytotoxicity assay) or 6 × 10^3^ (antiproliferative assay) *T*-cell mouse lymphoma cells in 100 μL of medium were added to each well, except for the medium control wells.

The adherent human fibroblast cells were seeded in 100 μL of EMEM medium overnight before each assay. Two-fold serial dilutions of the compounds (0.19–100 µM) were prepared in separate plates, then transferred to the plates containing the adherent cell lines.

The culture plates were further incubated at 37 °C for 24 h (cytotoxicity assay) or 72 h (antiproliferative assay); at the end of the incubation period, 20 μL of MTT solution (from a 5 mg/mL stock) was added to each well. After incubation at 37 °C for 4 h, 100 μL of SDS solution (10% in 0.01 M HCI) was added to each well and the plates were further incubated at 37 °C overnight. The cell growth was determined by measuring the optical density at 540 nm (ref. 630 nm) with a Multiscan EX ELISA reader (Thermo Labsystems, Cheshire, WA, USA). IC_50_ values were calculated by variable slope nonlinear regression using the log(inhibitor) vs. normalized response of GraphPad Prism 5.01 (GraphPad Software Inc., San Diego, CA, USA).

### 3.6. ABCB1 Inhibition Assay

ABCB1 inhibition was determined by the intracellular accumulation of rhodamin 123 as reported before [12]. Briefly, 2 × 10^6^ cells/mL of L5178Y and L5178Y_MDR_ cell lines were re-suspended in serum-free McCoy’s 5A medium and distributed in 0.5 mL aliquots into Eppendorf centrifuge tubes. The compounds were added at a final concentration of 2 or 20 μM and the samples were incubated for 10 min at room temperature. Tariquidar was used as positive control and DMSO as negative control; for the latter, no activity was observed. Subsequently, 10 μL (5.2 μM final concentration) of the ABCB1 substrate fluorescent dye rhodamine 123 was added and the cells were incubated further for 20 min at 37 °C, washed twice and re-suspended in 1 mL PBS for analysis. Fluorescence of the cell population was measured with a PartecCyFlow^®^ flow cytometer (Partec, Münster, Germany). Mean fluorescence intensity percentage was calculated for the treated vs. untreated L5178Y_MDR_ cells. Inhibition percentage for the treated cells was calculated from the corresponding values of the untreated L5178Y and L5178Y_MDR_ cells, representing 100% and 0% inhibition, respectively.

### 3.7. Combination Assay

The checkerboard microplate method was used to evaluate the compounds’ interaction with doxorubicin, as described before [12]. Briefly, doxorubicin (2 mg/mL, Teva Pharmaceuticals, Budapest, Hungary) was serially diluted in the horizontal direction (100 µL per well), and the ecdysteroid was subsequently diluted in the vertical direction (50 µL per well). L5178Y_MDR_ cells were re-suspended in culture medium and added to each test well in 50 µL to contain 1 × 10^4^ cells per well, and 50 µL of medium was added to the medium control wells. The plates were incubated for 72 h at 37 °C in a CO_2_ incubator and, at the end of the incubation period, the cell growth was determined by MTT assay as described above. Cell viability data were analyzed by the Calcusyn software using the Chou method [28], and drug interactions were expressed as combination index (CI) values, in which 0 < CI < 1, CI = 1, and CI > 1 refer to synergism, additivity, and antagonism, respectively.

## 4. Conclusions

On the example of ecdysteroids, we demonstrated that DAST-mediated transformation of natural products containing several OH groups is a valuable tool for diversity-oriented semi-synthesis of new bioactive compounds. Several unique ecdysteroid derivatives were obtained, and the DAST-mediated formation of a cyclopropane ring was described for the first time. Combinations of the different reactions that can be mediated by DAST manifested in the chemical complexity of the compounds obtained, and this allowed us to reveal valuable structure–activity relationships concerning their antitumor activity.

Altogether, the new ecdysteroids showed stronger antitumor properties than their parent compounds, and particularly compound **13** demonstrated a potent cytostatic activity against drug susceptible and multi-drug resistant cancer cell lines. Further, it showed significant synergism with doxorubicin on a Pgp expressing MDR cancer cell line without functional efflux pump inhibition. This makes it a promising lead compound for the future development of a possible combination therapy. 

## Figures and Tables

**Table 1:** ^1^H and ^13^C chemical shifts and *J*_C,F_ coupling constants of compounds **4**–**7**, **10**, and **11** in CDCl_3_.

	4		5		6		7		10		11	
no.	^1^H	^13^C	^1^H	^13^C	^1^H	^13^C	^1^H	^13^C	^1^H	^13^C	^1^H	^13^C
1β	1.37	37.8	1.56	37.0	1.48	37.8	1.55	37.1	1.28	37.5	1.30	37.3
α	2.04		2.19		2.25		2.22		1.96		1.98	
2	4.28	71.5	4.18	71.9	4.19	71.5	4.17	71.8	4.18	71.9	4.23	72.0
3	4.31	71.8	4.15	71.2	4.16	71.5	4.17	71.2	4.28	71.6	4.29	71.4
4β	2.17	26.9	2.05	28.2	2.17	29.0	2.08	28.2	2.17	26.7	2.09	28.2
α	1.86		1.86		1.58		1.86		1.77		1.91	
5	2.44	49.7	2.45	50.6	2.47	50.2	2.48	50.7	2.36	50.7	2.39	50.6
6		201.6		202.4		202.3		202.2		201.9		201.9
7	5.88	121.2	5.82	119.5	6.12	117.5	5.91	121.8 d 7 Hz	6.10	121.5	5.91	124.1 d 6.7 Hz
8		158.1		152.2		141.8		149.5 d 19 Hz		152.7		155.0 d 20 Hz
9	3.39	38.5		135.7		135.9		135.4	2.41	38.6	2.69	35.7
10		38.9		39.4		40.4		39.4		38.4		37.6
11β	5.78	124.5	6.17	130.8	6.17	130.1	6.18	130.6	1.72	20.5	1.63	20.6
α									1.84		1.84	
12β	6.33	133.7	2.41	36.0	2.75	40.9	2.46	35.9 d 6 Hz	2.31	38.7	1.91	30.4 d 4.5 Hz
α			2.87		2.57		2.81		1.74		2.13	
13		51.0		46.9		46.0		46.9 d 20 Hz		47.3		48.2 d 19.5 Hz
14		84.6		83.7		142.9		106.0 d 168 Hz		146.1		107.3 d 166 Hz
15β	2.08	28.6	2.05	31.1	6.28	128.4	2.04	28.5 d 25 Hz	5.97	128.2	1.98	28.4 d 28 Hz
α	1.75		1.86				2.14					
16β	2.30	21.8	2.31	21.5	2.98	32.0	2.34	21.5	2.93	31.6	2.32	21.1
α	2.09		2.01		2.48		2.03		2.36		1.95	
17	3.37	55.2	3.34	58.4	3.08	64.1	3.22	58.6	3.06	64.9	3.17	58.8
18	0.73	21.1	0.64	17.4	0.84	19.4	0.67	16.9 d 5 Hz	0.85	18.9	0.65	16.5 d 4 Hz
19	0.89	23.9	1.23	29.6	1.14	30.1	1.20	29.7	0.98	23.2	1.00	23.7
20		208.9		209.2		207.6		208.9		207.8		208.3
21	2.22	31.0	2.17	31.0	2.22	31.0	2.19	31.0	2.20	31.2	2.17	31.3
22				108.4		108.5		108.5		108.3		108.3
βMe	1.51		1.52	28.5	1.52	28.5	1.53	28.5	1.50	28.5	1.50	28.5
αMe	1.35		1.32	26.3	1.32	26.4	1.33	26.4	1.34	26.3	1.34	26.4
HO-14	2.37		1.92				1.92					

**Table 2:** ^1^H and ^13^C chemical shifts and *J*_C,F_ coupling constants of compounds **13**, **14**, and **17** in CDCl_3_; **15** in CD_3_OD; and **19** in DMSO-d_6_.

	13		14		15		17		19	
no.	^1^H	^13^C	^1^H	^13^C	^1^H	^13^C	^1^H	^13^C	^1^H	^13^C
1β	1.25	37.5	1.28	37.3	2.47	40.5	1.48	36.3	1.89	36.4 d 11 Hz
α	1.95		1.98		1.47		2.25		1.07	
2β		71.9		72.0		73.0		73.1	1.70	28.8 d 18 Hz
α	4.16		4.23		5.20		4.19		1.99	
3	4.26	71.6	4.29	71.3	3.64	71.8	2.01	35.7	4.39	92.8 d 174 Hz
4β	2.14	26.7	2.08	26.27	2.52	28.3	1.43	11.0	2.45	39.4 d 19 Hz
α	1.76		1.92		3.24		1.58		2.45	
5	2.34	50.7	2.39	50.7		131.3		40.2		139.4 d 12 Hz
6		202.2		202.2		144.8		189.5	5.40	123.0
7	6.06	120.9	5.88	123.3 7 Hz		181.4		144.6	1.99;1.54	31.9
8		153.3		155.4 d 20 Hz		125.0		118.3	1.46	31.87
9	2.38	38.7	2.69	35.8		164.5		136.2	0.93	50.0
10		38.5		37.7		41.8		44.2		36.5
11β	1.60	20.3	1.55	20.2	2.60	25.4	6.00	122.5	1.50	21.1
α	1.77		1.80		2.60				1.50	
12β	2.02	37.0	1.61	28.7 d 5 Hz	2.28	37.8	2.54	40.0	2.03	39.7
α	1.59		2.05		1.52		2.21		1.18	
13		46.9		47.9 d 19 Hz		47.9		46.5		42.3
14		147.5		105.5 d 166 Hz		142.4		143.5	1.00	56.7
15β	5.93	126.5	2.09	27.7 d 28 Hz	6.85	128.2	6.81	133.2	1.08	24.3
α			1.95						1.58	
16β	2.21	36.0	1.50	26.9	2.71	32.7	2.65	32.5	1.27	28.2
α	2.67		2.41		2.25		2.45		1.85	
17	4.41	60.3	4.60	55.4	1.99	56.5	1.95	55.2	1.10	56.2
18	0.89	17.2	0.71	15.3 d 4 Hz	1.07	18.3	0.95	19.7	0.69	11.9
19	0.96	23.2	1.00	23.4	1.44	27.3	1.19	26.4	1.04	19.3
20		170.2		170.1		77.3		82.9	1.38	35.8
21	2.02	23.4	2.02	23.6	1.26	20.5	1.16	21.2	0.93	18.7
22		108.3		108.3	3.38	78.7	3.71	81.4	1.35;1.00	36.2
βMe	1.49	28.5	1.50	28.6			1.52	28.5	1.52	
αMe	1.33	26.3	1.34	26.3			1.32	26.4	1.32	
23					1.62;1.31	27.3	1.45;1.42	23.4	1.34;1.15	23.8
24					1.81;1.43	42.4	1.54;1.38	41.4	1.14	39.5
25						71.4		68.7		28.0
26					1.17	28.9		29.0	0.87	22.6
27					1.20	30.0		29.8	0.88	22.8
28								106.3		
βMe							1.26	29.2		
αMe							1.35	26.9		
NH	5.80		5.57							
HO-7							9.29			

**Table 3:** Cytotoxicity and antiproliferative activity of compounds **3**, **5**–**7**, **9**–**11**, **13**, **14**, **16**, and **17** on mouse T-cell lymphoma cells and human fibroblasts (n = 3), and functional inhibition of the ABCB1 transporter (n = 1). For the ABCB1 inhibition, positive control: 20 nM of tariquidar (87.5% inhibition), negative control: 2% DMSO (0.2% inhibition).

	Cytotoxicity (IC_50_ ± SD [µM])			Antiproliferative Effect (IC_50_ ± SD [µM])			ABCB1 Inhibition (%)	
	L5178Y	L5178Y_MDR_	MRC-5	L5178Y	L5178Y_MDR_	MRC-5	2 µM	20 µM
**3**	>100	>100	>100	75.5 ± 4.1	>100	21.4 ± 0.2	−0.03	−0.35
**5**	>100	>100	>100	56.5 ± 6.0	80.2 ± 2.1	20.9 ± 3.6	0.20	−0.41
**6**	91.1 ± 6.8	>100	>100	31.2 ± 3.9	54.1 ± 2.3	>100	−0.38	0.13
**7**	38.6 ± 1.6	57.5 ± 6.3	>100	15.2 ± 1.9	20.0 ± 2.4	>100	−0.24	0.29
**9**	>100	>100	>100	49.2 ± 3.4	85.2 ± 5.1	>100	−0.40	−0.42
**10**	72.9 ± 5.6	83.0 ± 5	>100	27.7 ± 0.6	42.3 ± 1.5	>100	−0.38	−0.16
**11**	91.9 ± 9.3	81.9 ± 2.9	>100	31.8 ± 2.1	55.7 ± 5.9	10.0 ± 2.1	−0.57	−0.51
**13**	>100	>100	35.0 ± 3.2	4.6 ± 0.8	4.8 ± 0.6	12.0 ± 1.9	0.09	–0.01
**14**	>100	>100	>100	71.6 ± 1.5	65.9 ± 2.1	>100	0.20	−0.12
**16**	63.0 ± 4.4	78.7 ± 6.7	>100	50.3 ± 0.2	40.0 ± 3.4	67.2 ± 14.6	0.71	15.7
**17**	27.2 ± 1	35.9 ± 0.6	5. 9 ± 1.2	20.3 ± 1	14.3 ± 0.5	5.9 ± 1.2	1.08	45.5
**Doxorubicin**	0.30 ± 0.10	8.1 ± 2.8	1.7 ± 0.5	0.014 ± 0.002	0.71 ± 0.2	0.45 ± 0. 13	-	-

**Table 4:** Chemo-sensitizing activity of compounds **7**, **10**, **11**, **13**, and **14** on the L5178Y_MDR_ cell line towards doxorubicin at 50, 75, and 90% of growth inhibition (ED_50_, ED_75_, and ED_90_, respectively). CI: combination index; CI_avg_: weighted average CI value; CI_avg_ = (CI_50_ + 2CI_75_ + 3CI_90_)/6. CI < 1, CI = 1, and CI > 1 represent synergism, additivity, and antagonism, respectively. Dm, m, and r represent antilog of the x-intercept, slope, and linear correlation coefficient of the median-effect plot, respectively.

	Drug Ratio	CI at			Dm	m	r	CI_avg_
		ED50	ED75	ED90				
**7**	11.6:1	0.98	0.76	0.59	4.05	2.18	0.940	0.71
	23.1:1	1.16	1.26	1.37	7.83	1.32	0.971	1.30
	46.4:1	1.17	0.84	0.60	11.56	2.64	0.974	0.78
**10**	23.2:1	0.75	0.50	0.33	6.50	2.20	0.988	0.46
	46.4:1	0.87	0.51	0.30	10.99	2.76	0.983	0.46
	92.8:1	0.86	0.55	0.35	14.02	2.14	0.999	0.50
	185.6:1	1.02	0.61	0.36	19.47	2.37	0.989	0.55
**11**	23.2:1	0.78	0.56	0.41	7.00	2.43	0.964	0.52
	46.4:1	0.81	0.55	0.39	11.66	3.07	0.996	0.51
	92.8:1	0.77	0.53	0.38	15.83	3.42	0.991	0.49
	185.6:1	0.93	0.66	0.48	24.06	3.51	0.991	0.61
	371.2:1	0.95	0.72	0.55	28.63	3.27	0.968	0.67
**13**	23.2:1	0.64	0.55	0.49	8.17	2.65	0.992	0.53
	46.4:1	0.67	0.51	0.39	14.17	4.18	0.963	0.48
	92.8:1	0.55	0.51	0.47	17.54	2.60	0.966	0.50
	185.6:1	0.71	0.68	0.65	29.89	2.54	0.997	0.67
**14**	23.2:1	0.85	0.73	0.63	9.04	1.78	0.994	0.70
	46.4:1	0.86	0.72	0.60	13.53	1.99	0.998	0.68
	92.8:1	1.00	0.86	0.74	20.47	1.99	0.996	0.82
	185.6:1	1.28	1.26	1.24	30.97	1.65	0.994	1.25

**Table 5:** HPLC and flash chromatographic methods used for the purification of the compounds. X g silica: “RediSep” flash chromatographic columns (TELEDYNE Isco, Lincoln, NE, USA). The following HPLC columns were used (each purchased from Phenomenex Inc., Torrance, CA, USA). “XB-C18”: Kinetex^®^, 5 µm, XB-C18, 100 Å, 250 × 21.2 mm; “biphenyl”: Kinetex^®^, 5 µm, Biphenyl, 100 Å, 250 × 21.2 mm; “phenyl-hexyl”: Luna^®^, 5 µm, Phenyl-Hexyl, 100 Å, 250 × 10 mm; “Luna silica”: Luna^®^, 5 µm, Silica (2) 100 Å 250 × 21.2 mm. Yields refer to the isolated yield%. Solvent ratios are given in *v*/*v*.

Compound (Yield)	Column	Flow Rate	Elution	Detection
**2** (70.2%)	24 g silica	35 mL/min	CH_2_Cl_2_:CH_3_OH (A:B) 12% B (40 min)	254 nm
**3** (78.9%)	24 g silica	35 mL/min	CH_2_Cl_2_:ethyl-acetate (A:B) 2→4% B (60 min)	254 nm
**4** (6.1%)	*(1)* biphenyl *(2)* phenyl-hexyl	15 mL/min	*(1)* water:CH_3_CN (A:B) 26% B 40 min-40% B 40–60 min *(2)* water:CH_3_CN (A:B) 44% B (30 min)	254 nm
**5** (19.4 %)				
**6** (9.4%)				
**7** (3.4%)				
**9** (55.8%)	80 g silica	60 mL/min	CH_2_Cl_2_:CH_3_OH (A:B) 0→3% B (60 min)	254 nm
**10** (20.3%)	XB-C18	15 mL/min	water:CH_3_CN (A:B) 42% B (40 min)	254 nm 300 nm
**11** (12.0%)				
**12** (82.0%)	24 g silica	35 mL/min	CH_2_Cl_2_:CH_3_OH (A:B) 0→5% B (70 min)	254 nm
**13** (16.6%)	*(1)* XB-C18 *(2)* Luna silica	15 mL/min	*(1)* water: CH_3_CN (A:B) 35% B (40 min) *(2)* cyclohexane:(2-propanol:water 97:3) (A:B) 17% B (40 min)	245 nm 300 nm
**14** (6.1%)				
**16** (84.6%)	80 g silica	60 mL/min	*n*-hexane:(acetone:2-propanol 8:2) (A:B) 10→15% B (60 min)	254 nm
**17** (6.4%)	*(1)* Luna silica *(2)* XB-C18	15 mL/min	*(1)* cyclohexane:2-propanol (A:B) 3% B (40 min) *(2)* water:CH_3_CN (A:B) 63% B (40 min)	254 nm 366 nm
**19** (43.9%)	40 g silica	40 mL/min	100% *n*-hexane (60 min)	210 nm

## Data Availability

Data is contained within the article or Supplementary Material; raw datasets are available from the authors upon request.

## Supplementary Materials

The following are available online at https://www.mdpi.com/article/10.3390/ijms23073447/s1.
