# Supplementary material for: Diversity-Oriented Synthesis Catalyzed by Diethylaminosulfur-Trifluoride—Preparation of New Antitumor Ecdysteroid Derivatives
Source: Int J Mol Sci. 2022 Mar 22;23(7):3447. doi: 10.3390/ijms23073447 (PMC8998355; doi:10.3390/ijms23073447)

Supplementary Materials to Diversity-oriented synthesis catalyzed by diethylaminosulfur-trifluoride – preparation of new antitumor ecdysteroid derivatives

Máté Vágvölgyi [1,a], Endre Kocsis [1,a], Márta Nové [2], Nikoletta Szemerédi [2], Gabriella Spengler [2], Zoltán Kele [3], Róbert Berkecz [4], Tamás Gáti [5], Gábor Tóth [6,*], and Attila Hunyadi [1,7,*]

[1] Institute of Pharmacognosy, Interdisciplinary Excellence Centre, University of Szeged, H-6720 Szeged, Hungary; vagvolgyi.mate@pharmacognosy.hu (M.V.); endrukocsis2@gmail.com (E.K.)
[2] Department of Medical Microbiology and Immunobiology, University of Szeged, H-6720 Szeged, Hungary; nove.marta@gmail.com (M.N.); szemeredi.nikoletta@med.u-szeged.hu (N.S.); spengler.gabriella@med.u-szeged.hu (G.S.)
[3] Department of Medical Chemistry, University of Szeged, H-6720, Szeged, Hungary; kele.zoltan@med.u-szeged.hu
[4] Institute of Pharmaceutical Analysis, University of Szeged, H- 6720 Szeged, Hungary; berkecz.robert@szte.hu
[5] Servier Research Institute of Medicinal Chemistry (SRIMC), H-1031 Budapest, Hungary; tamas.gati@hu.netgrs.com
[6] Department of Inorganic and Analytical Chemistry, NMR Group, Budapest University of Technology and Economics, H-1111 Budapest, Hungary
[7] Interdisciplinary Centre of Natural Products, University of Szeged, H-6720 Szeged, Hungary
[a] shared first authorship by M.V. and E.K.
* Correspondence: hunyadi.a@pharmacognosy.hu (A.H.); and drtothgabor@t-online.hu (G.T.)

Table of contents

Figure S1. Compound **4**, ^{1}H NMR $CDCl_3$ 600 MHz and selTOCSY on H-5, H-12 and H-7.
Figure S2. Compound **4**, steric proximities detected by selNOESY on signals αMe, H_3-19 and H_3-18.
Figure S3. Compound **4**, DEPTQ 150 MHz.
Figure S4. Compound **4**, edHSQC and edHSQC CH_2 section.
Figure S5. Compound **4**, HMBC and HMBC CH_3 section.
Figure S6. Compound **5**, ^{1}H NMR $CDCl_3$ 600 MHz and selTOCSY on H-17.
Figure S7. Compound **5**, Steric proximities detected by selNOESY on signals αMe, H_3-19 and H_3-18.
Figure S8. Compound **5**, DEPTQ 150 MHz.
Figure S9. Compound **5**, edHSQC and edHSQC CH_2 section.
Figure S10. Compound **5**, HMBC and HMBC CH_3 section.
Figure S11. Compound **6**,^{1}H NMR $CDCl_3$ 600 MHz.
Figure S12. Compound **6**, steric proximities detected by selNOESY on signals αMe, H_3-19 and H_3-18.
Figure S13. Compound **6**, DEPTQ 150 MHz.
Figure S14. Compound **6**, edHSQC and edHSQC CH_2 section.

Figure S15. Compound **6**, HMBC and HMBC CH_3 section.
Figure S16. Compound **7**, ^{1}H NMR $CDCl_3$ 500 MHz and steric proximities detected by selROESY on signals H_3-19 and H_3-18.
Figure S17. Compound **7**, DEPTQ 125 MHz.
Figure S18. Compound **7**, edHSQC and edHSQC CH_2 section.
Figure S19. Compound **7**, HMBC and HMBC CH_3 section.
Figure S20. Compound **10**, ^{1}H NMR $CDCl_3$ 600 MHz and selTOCSY on H-15 and Hα-1.
Figure S21. Compound **10**, steric proximities detected by selNOESY on signals βMe, H_3-19 and H_3-18.
Figure S22. Compound **10**, DEPTQ 150 MHz.
Figure S23. Compound **10**, edHSQC.
Figure S24. Compound **10**, HMBC.
Figure S25. Compound **10**, edHSQC CH_2 section and HMBC CH_3 section.
Figure S26. Compound **11**, ^{1}H NMR $CDCl_3$ 600 MHz.
Figure S27. Compound **11**, steric proximities detected by selNOESY on signals βMe, H_3-19 and H_3-18.
Figure S28. Compound **11**, DEPTQ 150 MHz.
Figure S29. Compound **11**, edHSQC.
Figure S30. Compound **11**, HMBC.
Figure S31. Compound **11**, edHSQC CH_2 section and HMBC CH_3 section.
Figure S32. Compound **13**, ^{1}H NMR $CDCl_3$ 600 MHz and selTOCSY on H-17 and H-3.
Figure S33. Compound **13**, steric proximities detected by selROESY on signals H_3-19 and H_3-18.
Figure S34. Compound **13**, DEPTQ 150 MHz.
Figure S35. Compound **13**, edHSQC.
Figure S36. Compound **13**, HMBC.
Figure S37. Compound **13**, edHSQC CH_2 section and HMBC CH_3 section.
Figure S38. Compound **14**, ^{1}H $CDCl_3$ 600 MHz.
Figure S39. Compound **14**, Identification of spin-systems of **A**, **C** and **D** rings by selTOCSY on H-3, H-9 and H-17.
Figure S40. Compound **14**, steric proximities detected by selNOE on CH_3-19, CH_3-18 and NH signals.
Figure S41. Compound **14**, DEPTQ 150 MHz.
Figure S42. Compound **14**, edHSQC.
Figure S43. Compound **14**, edHSQC section with inserted selTOCSY on H-17.
Figure S44. Compound **14**, HMBC.
Figure S45. Compound **17**, ^{1}H DMSO-d_6 600 MHz.
Figure S46. Compound **17**, Steric proximities detected by selNOE on CH_3-19 and CH_3-18 and selTOCSY on H-2 signals.

Figure S47. Compound **17**, DEPTQ 150 MHz.
Figure S48. Compound **17**, HSQC.
Figure S49. Compound **17**, HMBC and HMBC CH_3 section.
Figure S50. Compound **19**, ^{1}H $CDCl_3$ 500 MHz.
Figure S51. Compound **19**, ^{1}H,^{1}H-C0SY and selROE on Hα-3.
Figure S52. Compound **19**, APT 125 MHz.
Figure S53. Compound **19**, HSQC section with inserted band-selective HSQC measurements of 37–36 and 40– 39ppm.
Figure S54. Compound **19**, edHSQC section with inserted selROE on H_3-18.
Figure S55. Compound **19**, HMBC and HMBC CH_3 section.
Figure S56. Compound **4**, HR-MS spectra.
Figure S57. Compound **5**, HR-MS spectra.
Figure S58. Compound **6**, HR-MS spectra.
Figure S59. Compound **7**, HR-MS spectra.
Figure S60. Compound **10**, HR-MS spectra.
Figure S61. Compound **11**, HR-MS spectra.
Figure S62. Compound **13**, HR-MS spectrum.
Figure S63. Compound **14**, HR-MS spectrum.
Figure S64. Compound **17**, HR-MS spectrum.
Figure S65. Compound **3**, HPLC chromatogram at its UV absorbance maximum (λ=242.6 nm). Purity: 95.1 %.
Figure S66. Compound **4**, HPLC chromatogram at its UV absorbance maximum (λ=300 nm). Purity: 97.5 %.
Figure S67. Compound **5**, HPLC chromatogram at its UV absorbance maximum (λ=300 nm). Purity: 97.5 %.
Figure S68. Compound **6**, HPLC chromatogram at its UV absorbance maximum (λ=327.5 nm). Purity: 95.2 %.
Figure S69. Compound **7**, HPLC chromatogram at its UV absorbance maximum (λ=300 nm). Purity: 95.4 %.
Figure S70. Compound **9**, HPLC chromatogram at its UV absorbance maximum (λ=242.6 nm). Purity: 98.0 %.
Figure S70. Compound **10**, HPLC chromatogram at its UV absorbance maximum (λ=300 nm). Purity: 99.5 %.
Figure S72. Compound **11**, HPLC chromatogram at its UV absorbance maximum (λ=300 nm). Purity: 99.3 %.
Figure S73. Compound **13**, HPLC chromatogram at its UV absorbance maximum (λ=300 nm). Purity: 98.4 %.
Figure S74. Compound **14**, HPLC chromatogram at its UV absorbance maximum (λ=240 nm). Purity: 97.7 %.
Figure S75. Compound **16**, HPLC chromatogram at its UV absorbance maximum (λ=220.8 nm). Purity: 98.4 %.
Figure S76. Compound **17**, HPLC chromatogram at its UV absorbance maximum (λ=358.5 nm). Purity: 98.1 %.

Figure S1. Compound **4**, ^{1}H NMR $CDCl_3$ 600 MHz and selTOCSY on H-5, H-12 and H-7.

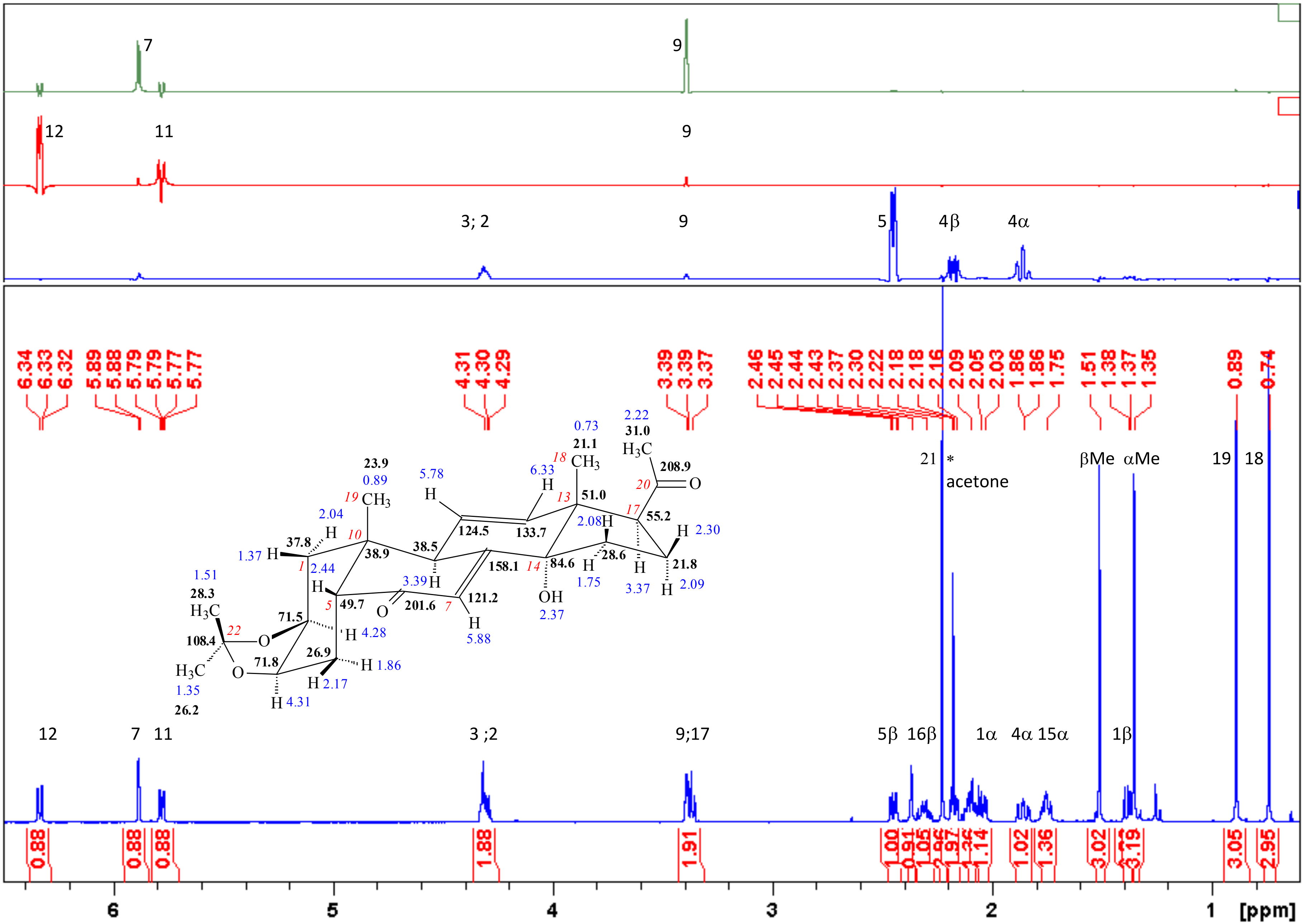

Figure S2. Compound **4**, steric proximities detected by selNOESY on signals αMe, H_3-19 and H_3-18.

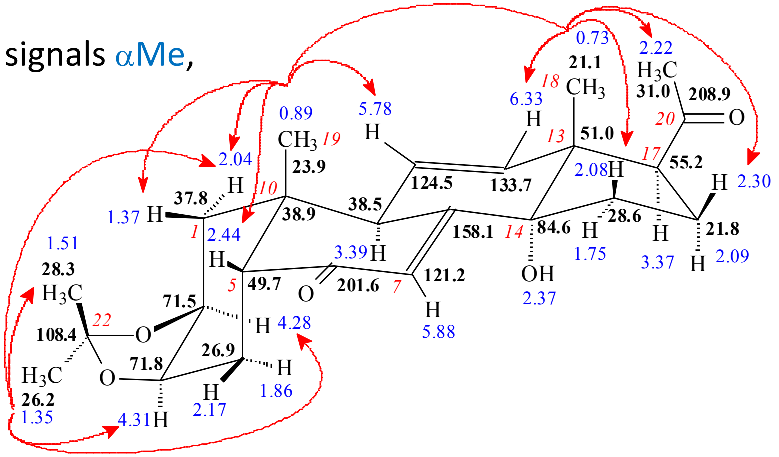

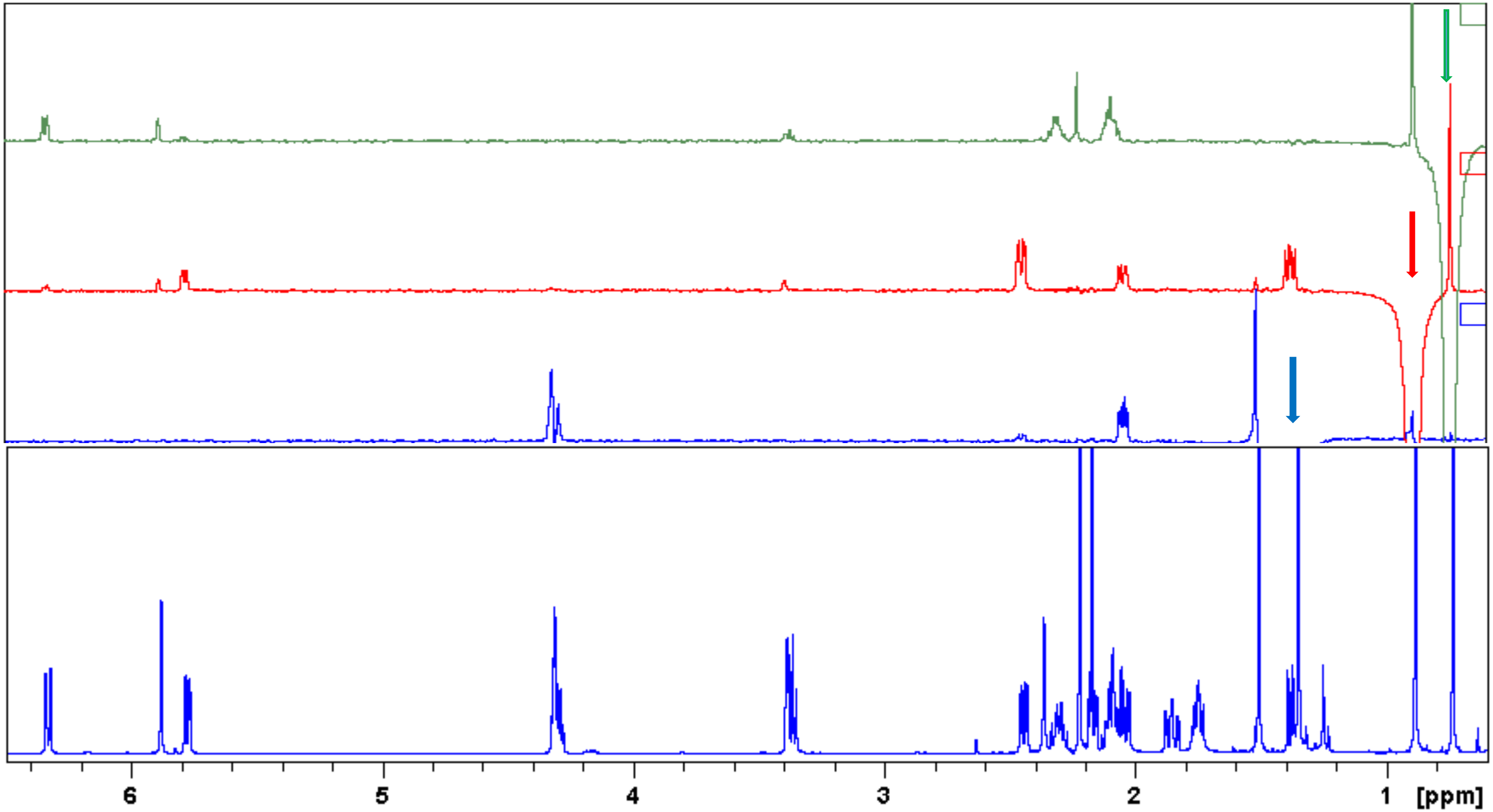

Figure S3. Compound **4**, DEPTQ 150 MHz.

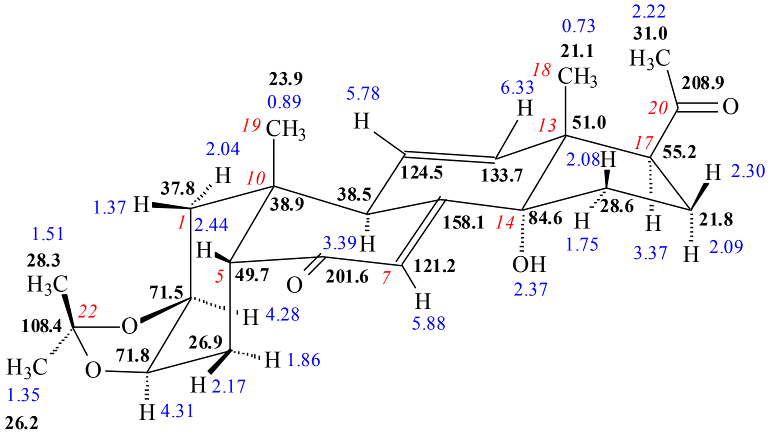

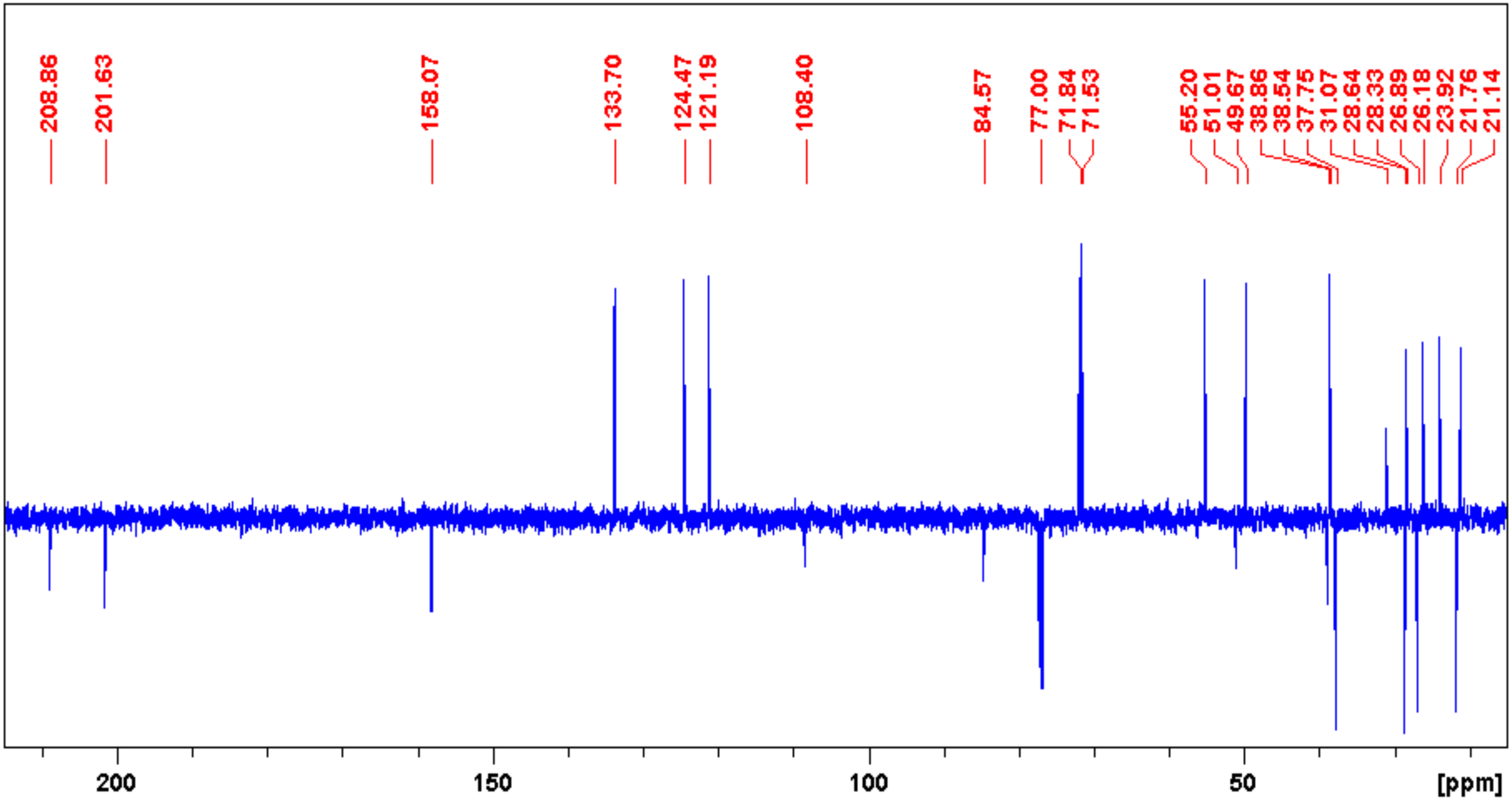

Figure S4. Compound **4**, edHSQC and edHSQC CH_2 section.

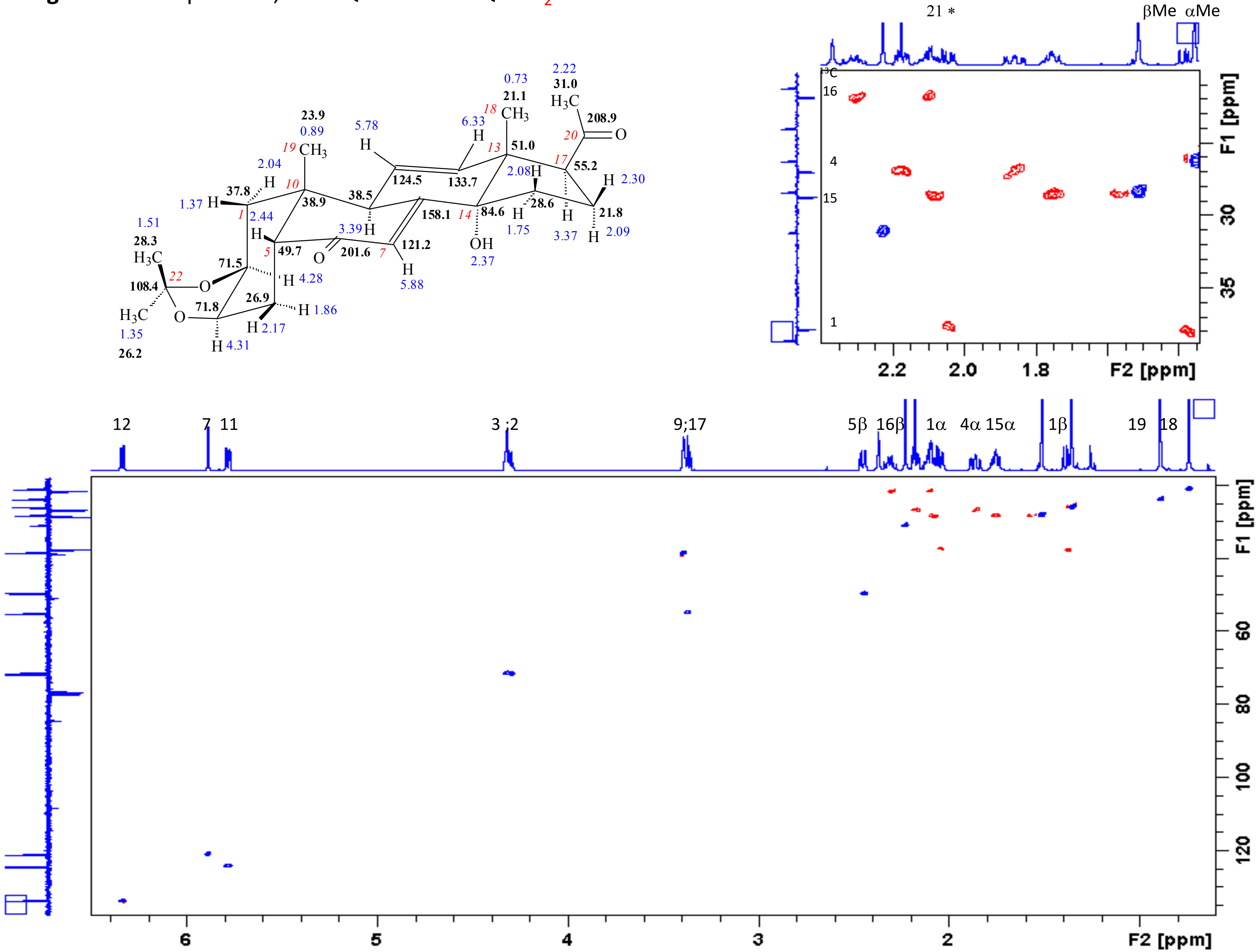

Figure S5. Compound **4**, HMBC and HMBC CH_3 section.

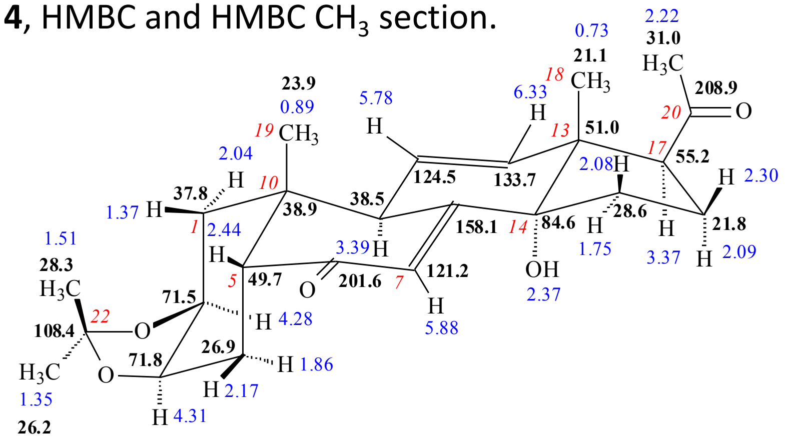

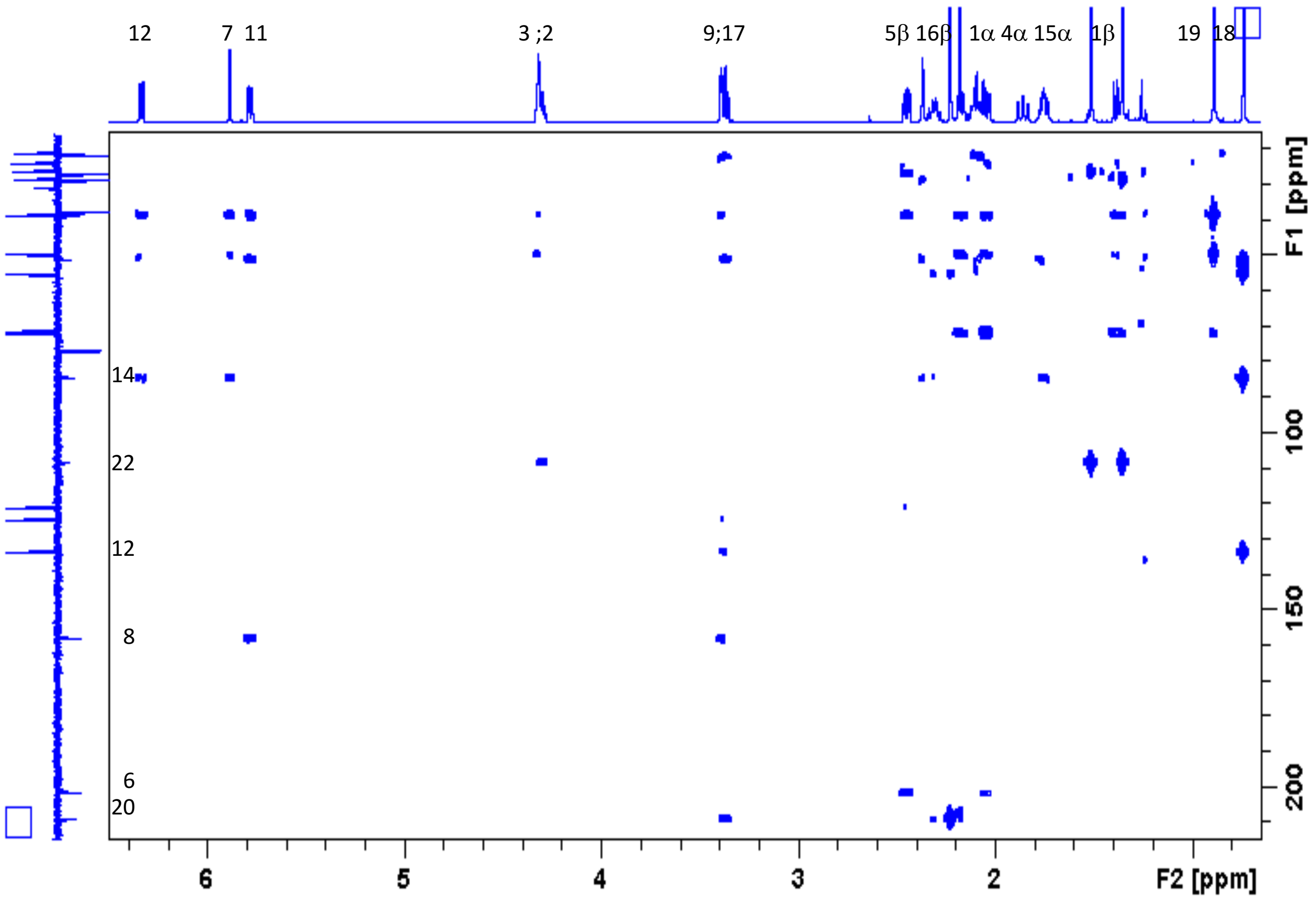

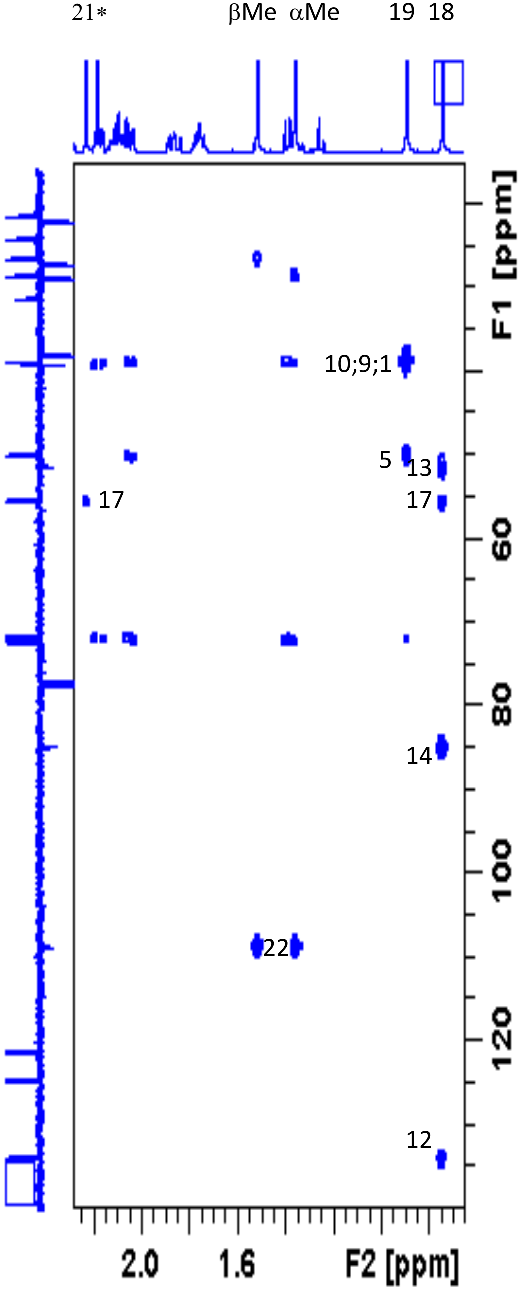

Figure S6. Compound **5**, ^{1}H NMR $CDCl_3$ 600 MHz and selTOCSY on H-17.

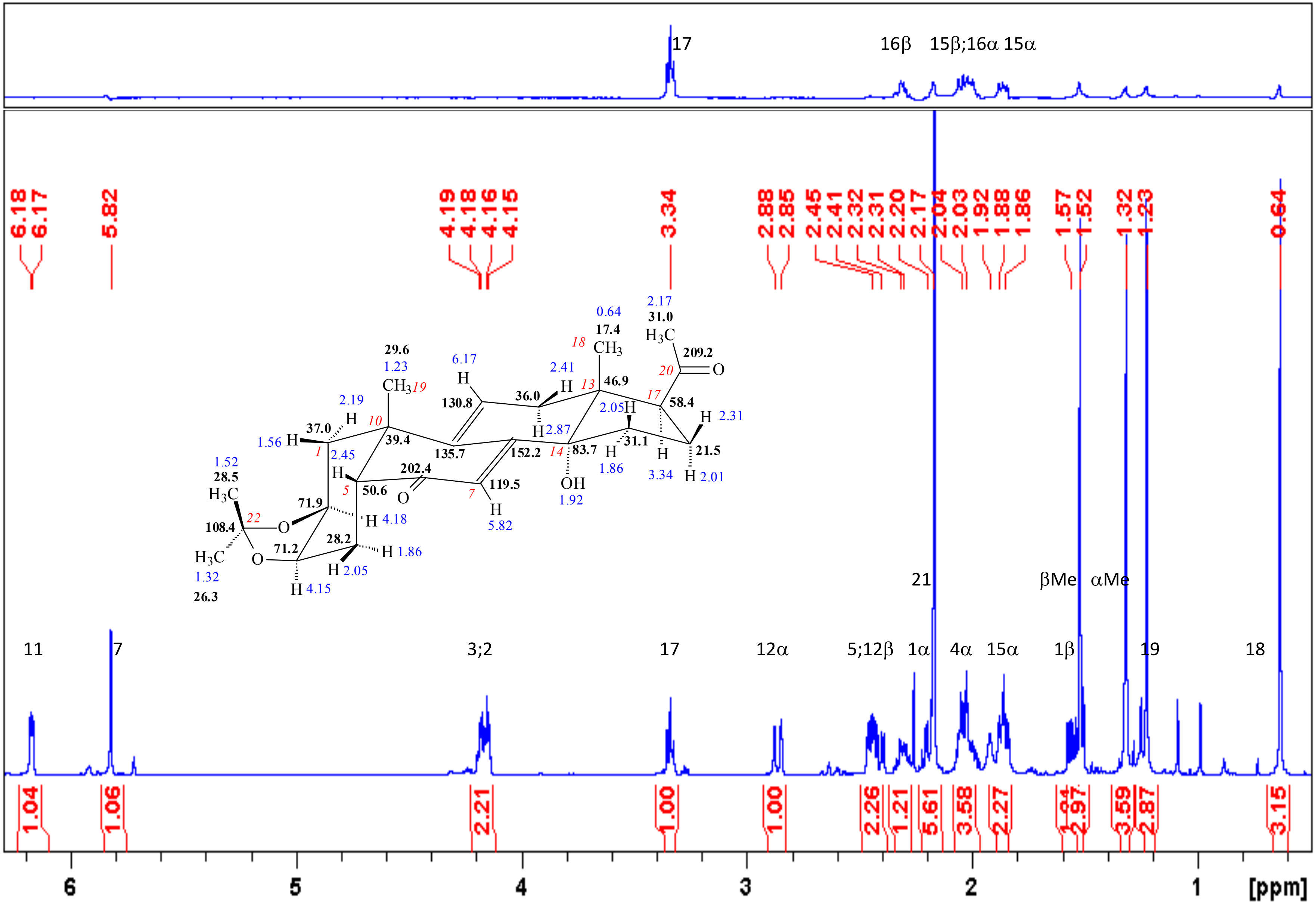

Figure S7. Compound **5**, steric proximities detected by selNOESY on signals αMe, H_3-19 and H_3-18.

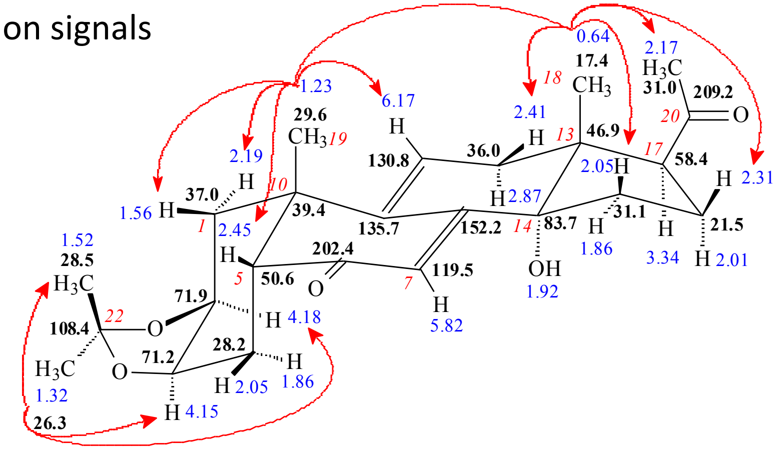

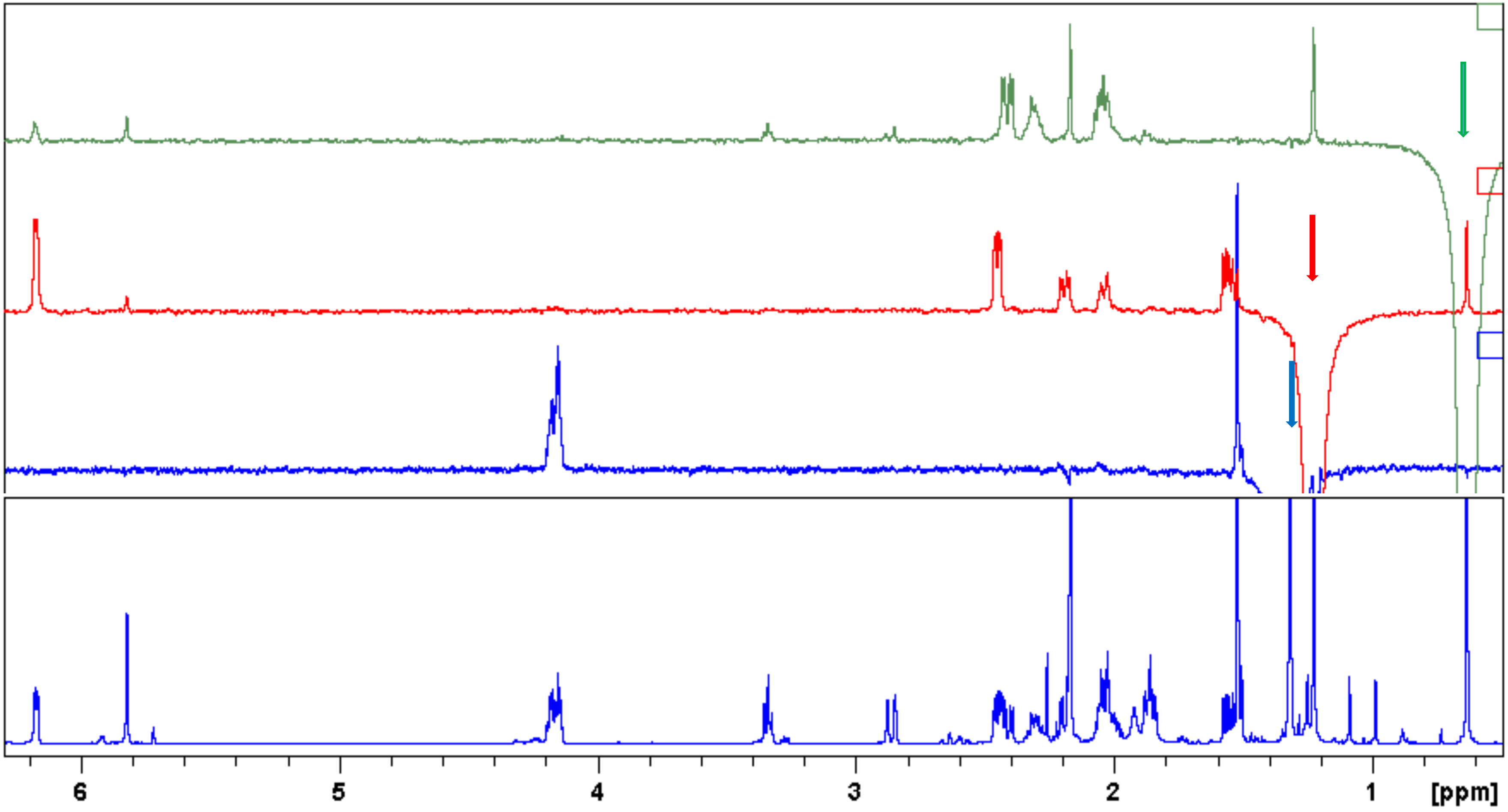

Figure S8. Compound **5**, DEPTQ 150 MHz.

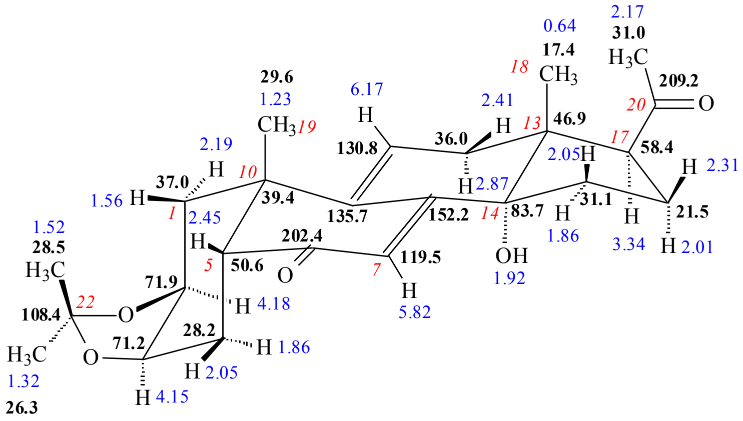

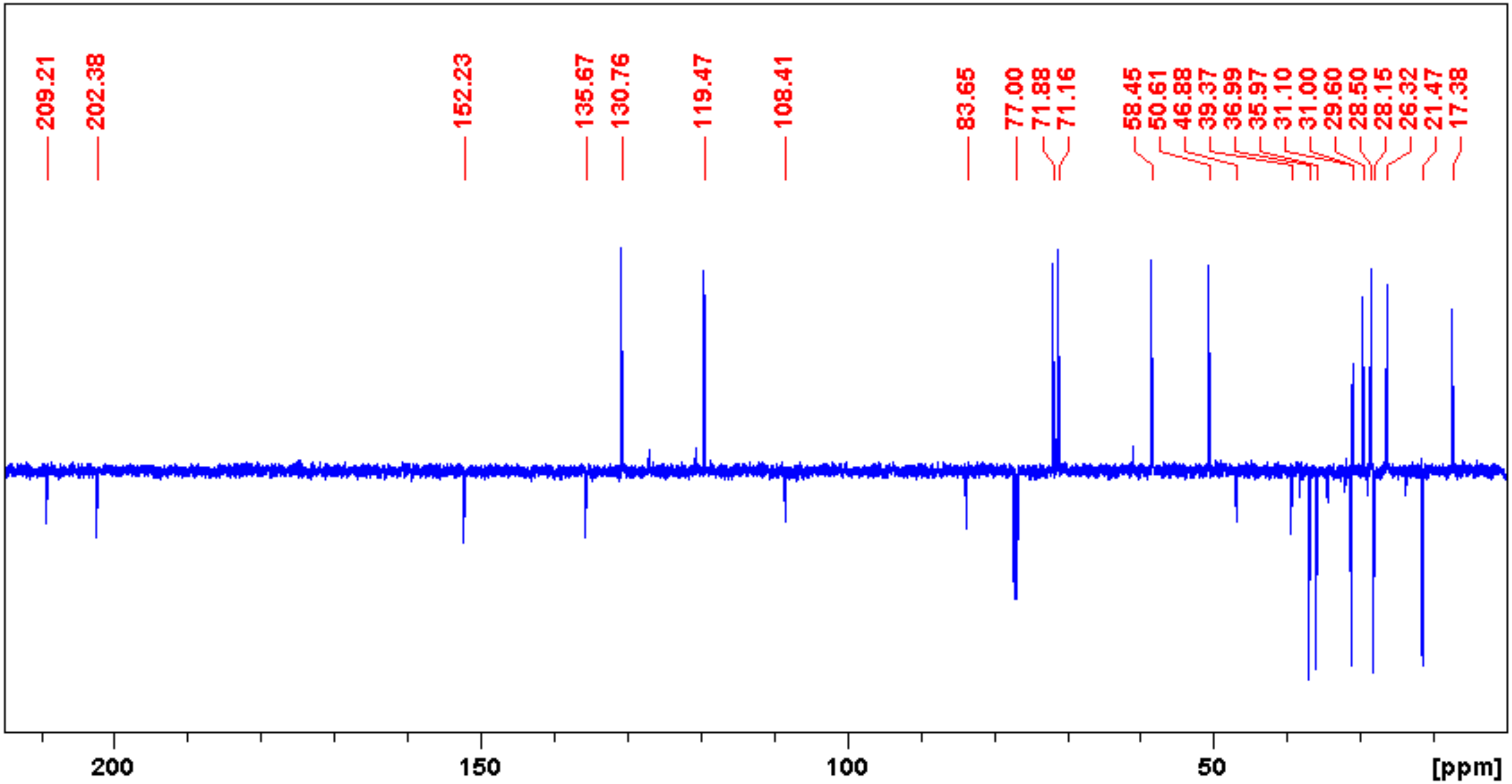

Figure S9. Compound **5**, edHSQC and edHSQC CH_2 section.

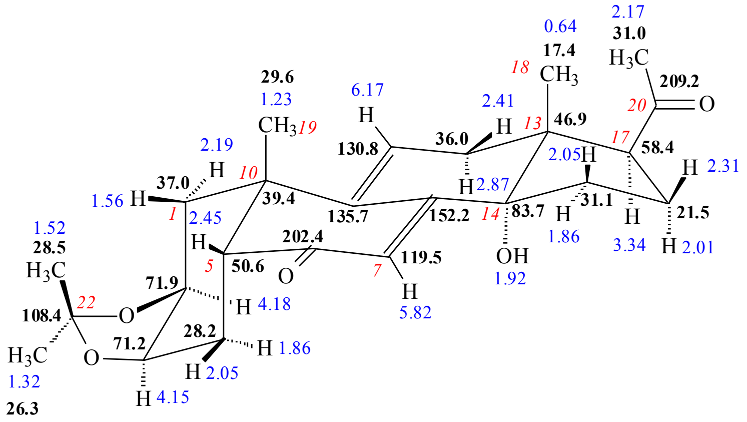

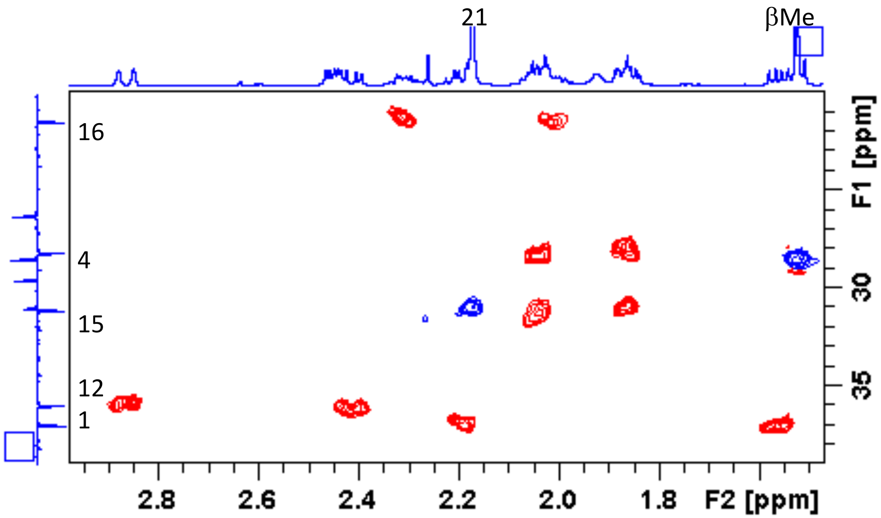

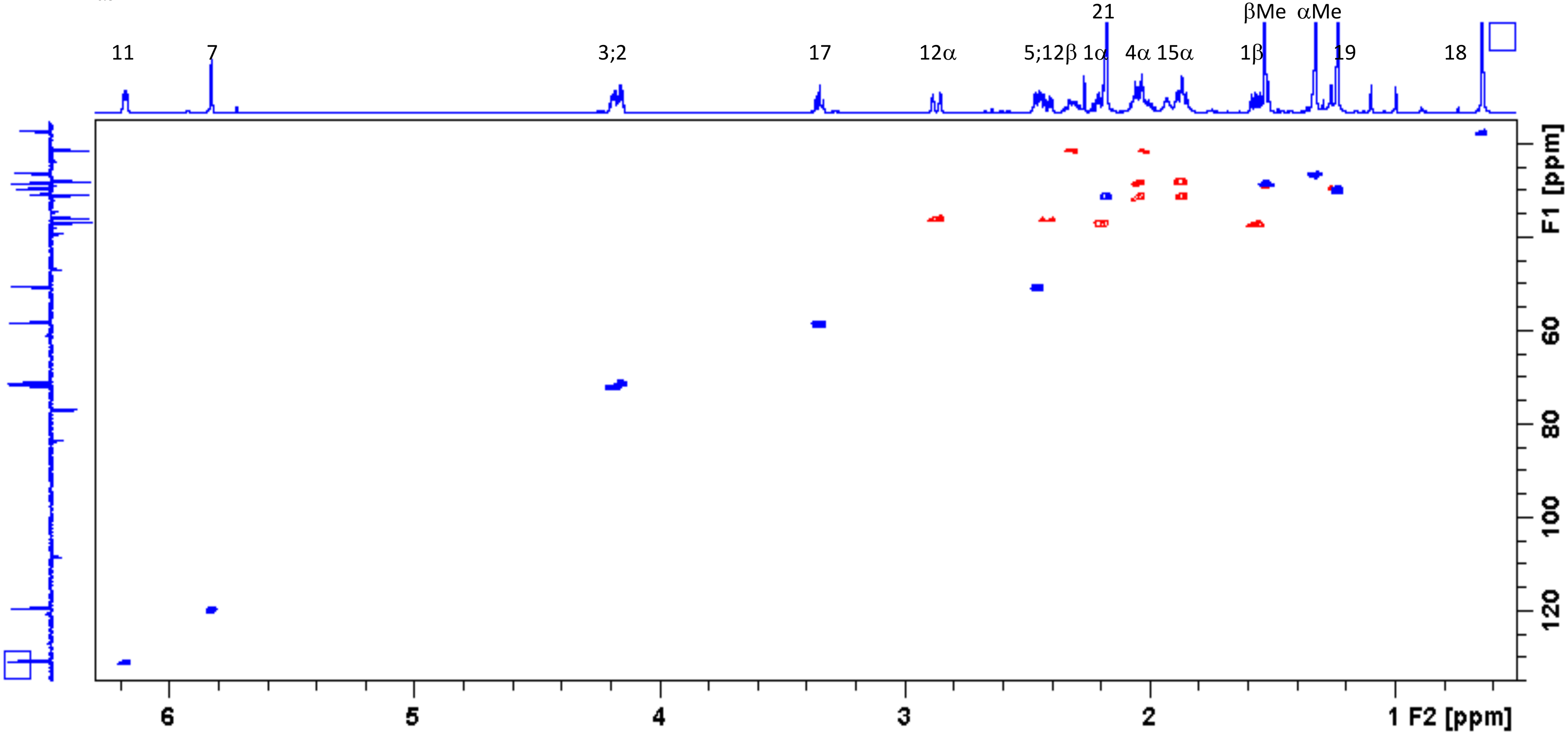

Figure S10. Compound **5**, HMBC and HMBC CH_3 section.

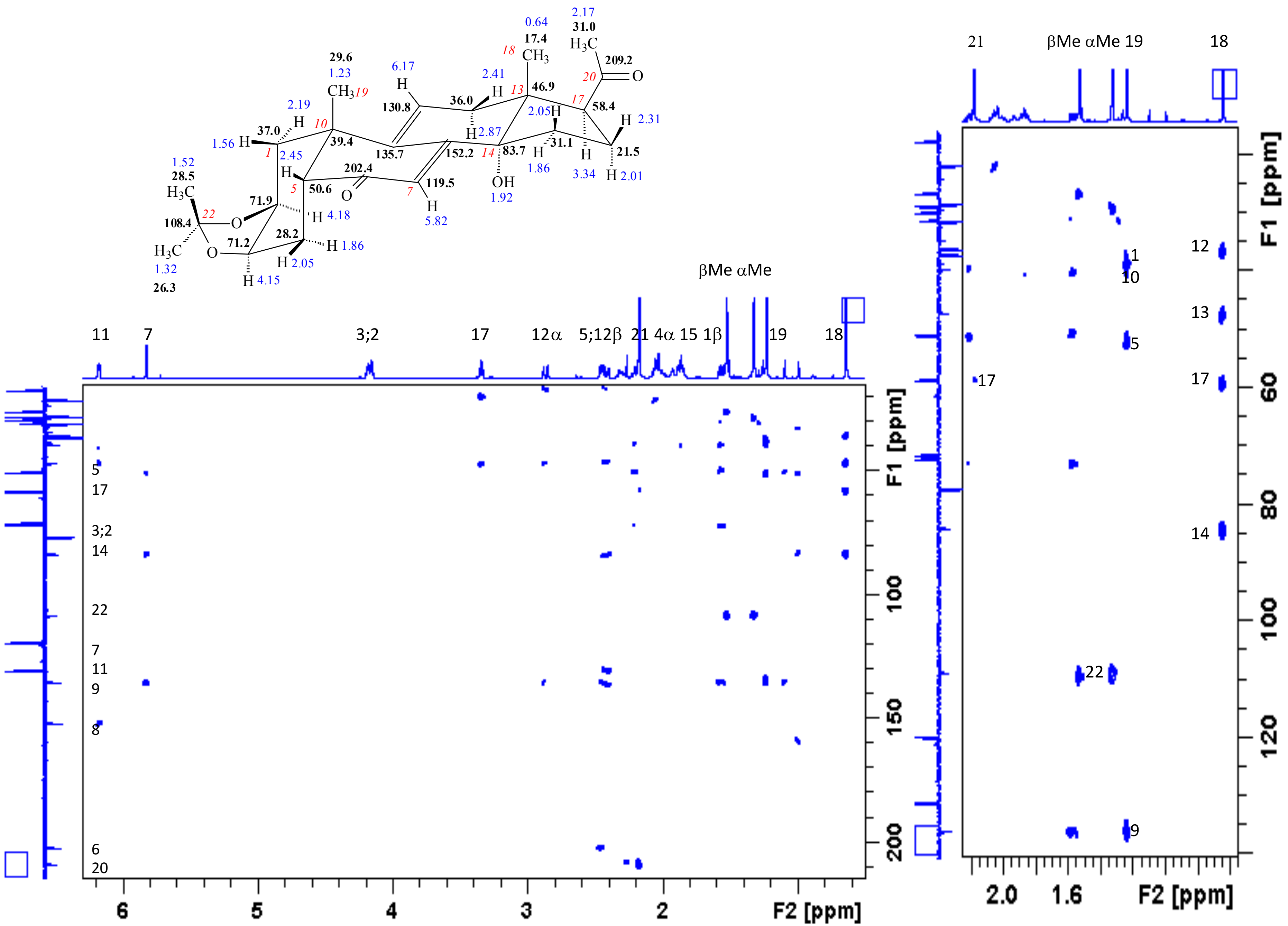

Figure S11. Compound **6**, ^1H NMR $CDCl_3$ 600 MHz.

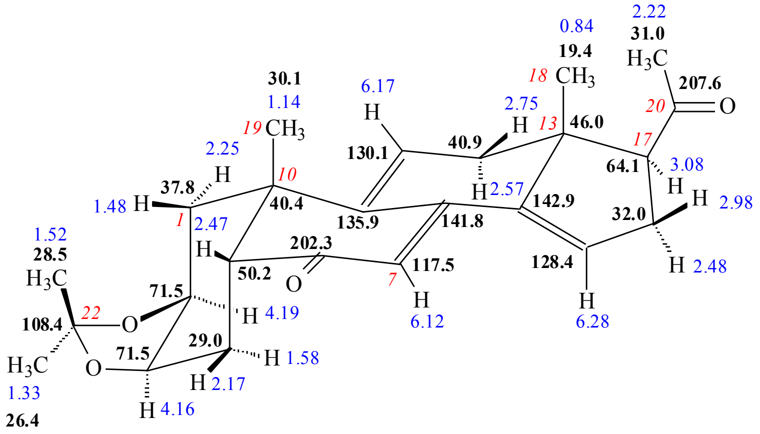

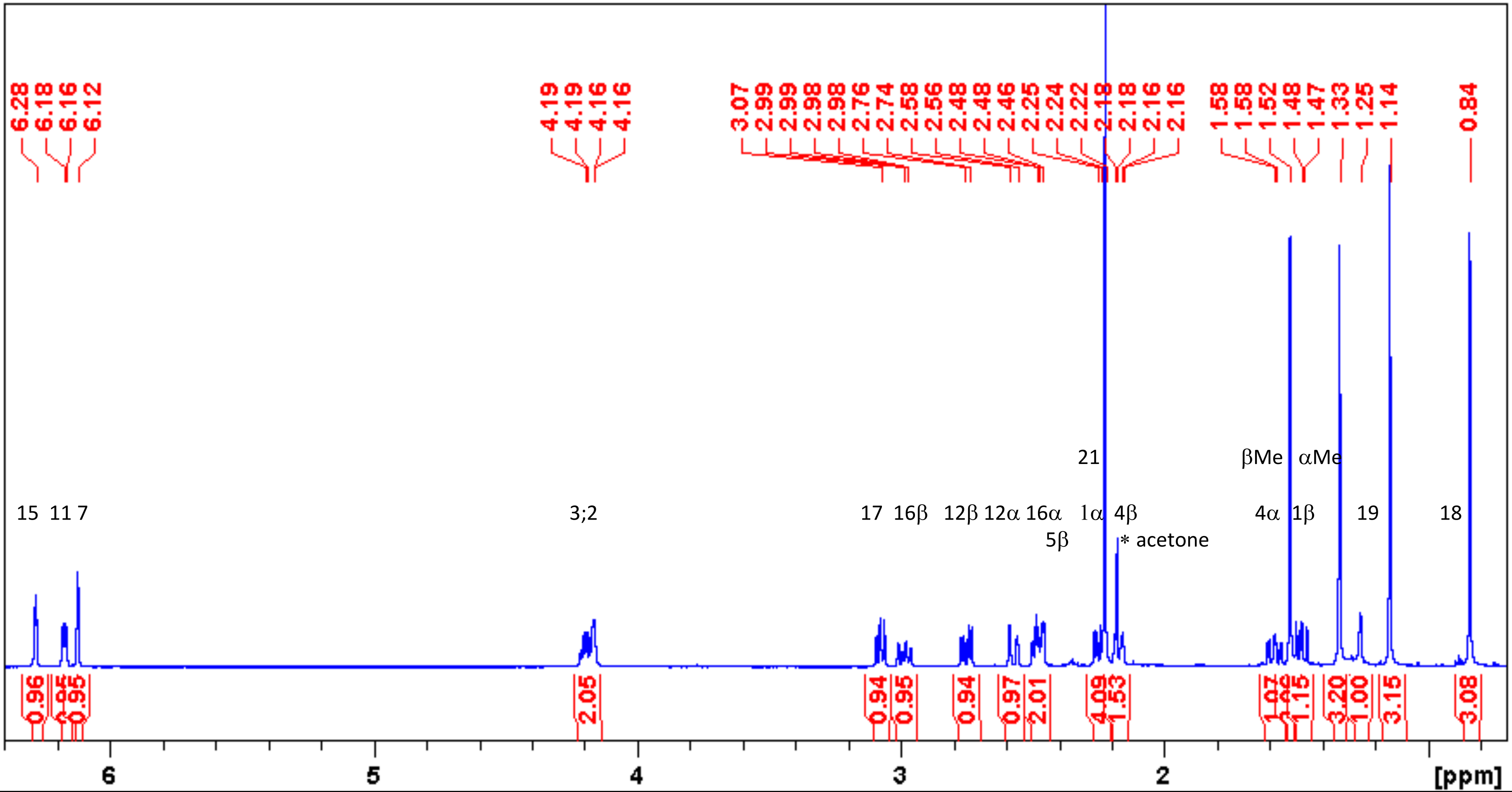

Figure S12. Compound **6**, steric proximities detected by selNOESY on signals αMe, H_3-19 and H_3-18.

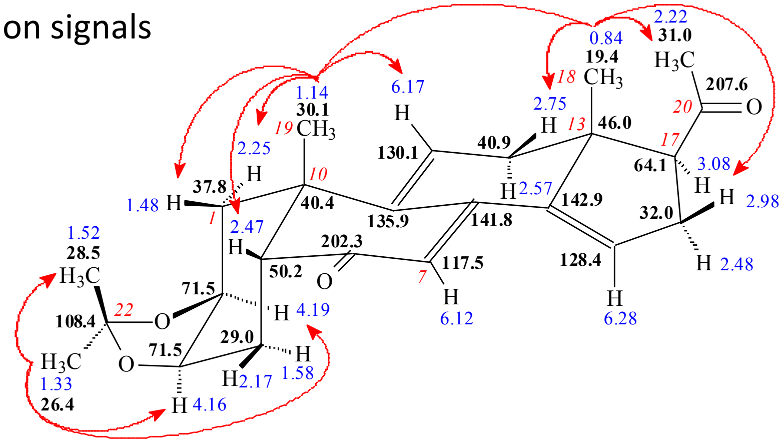

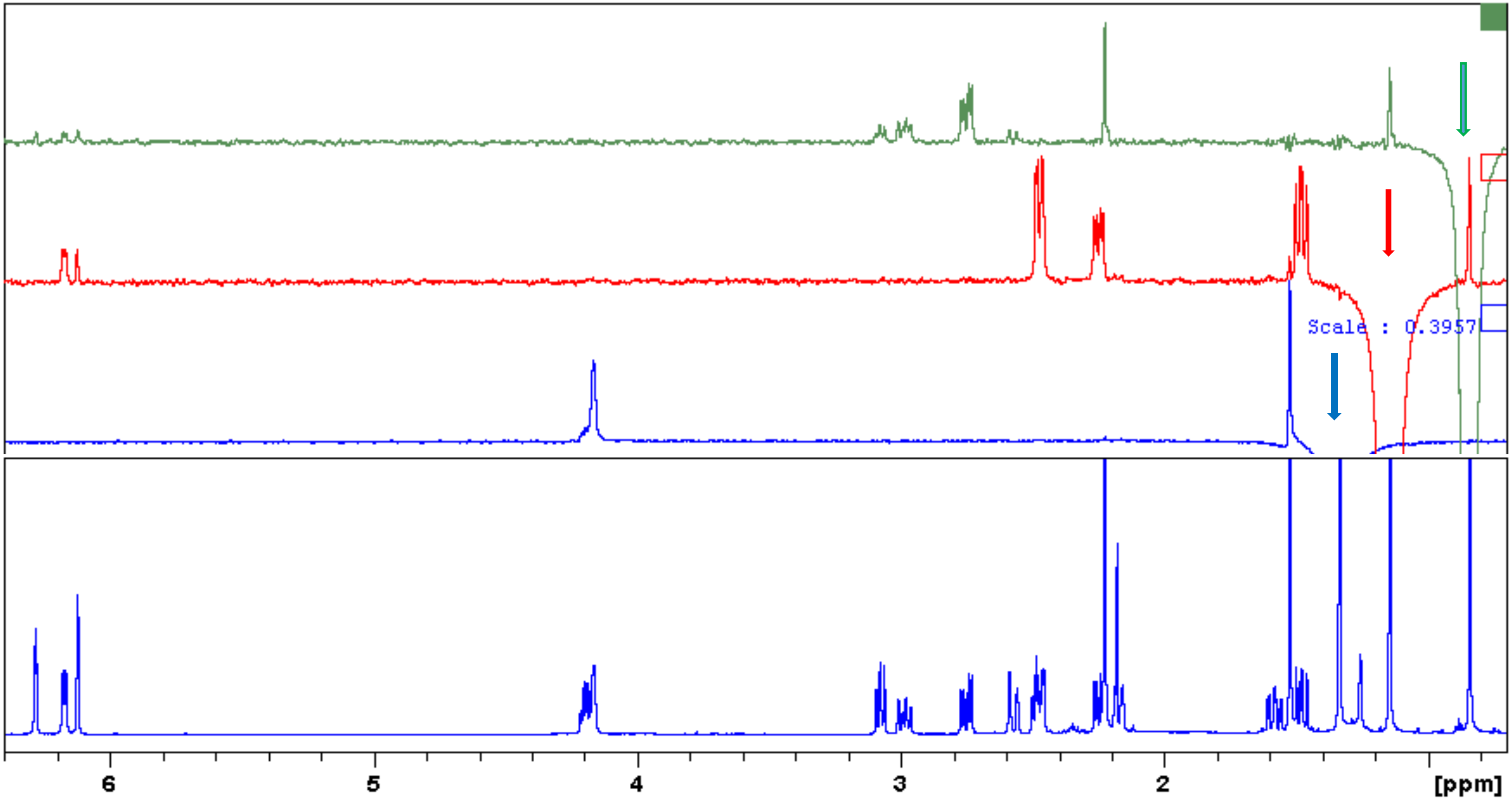

Figure S13. Compound **6**, DEPTQ 150 MHz.

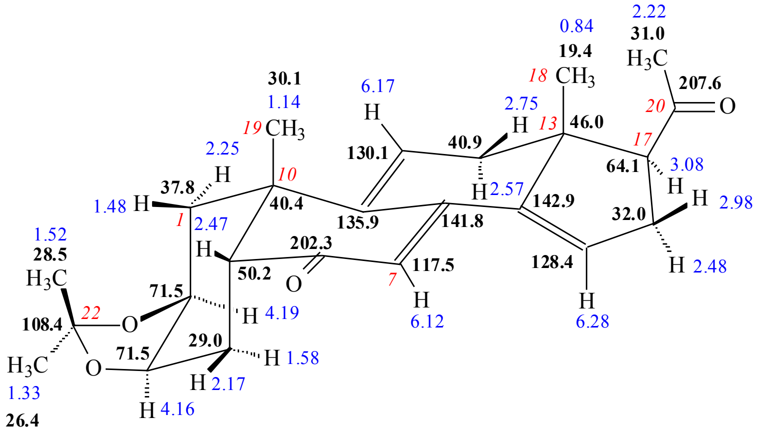

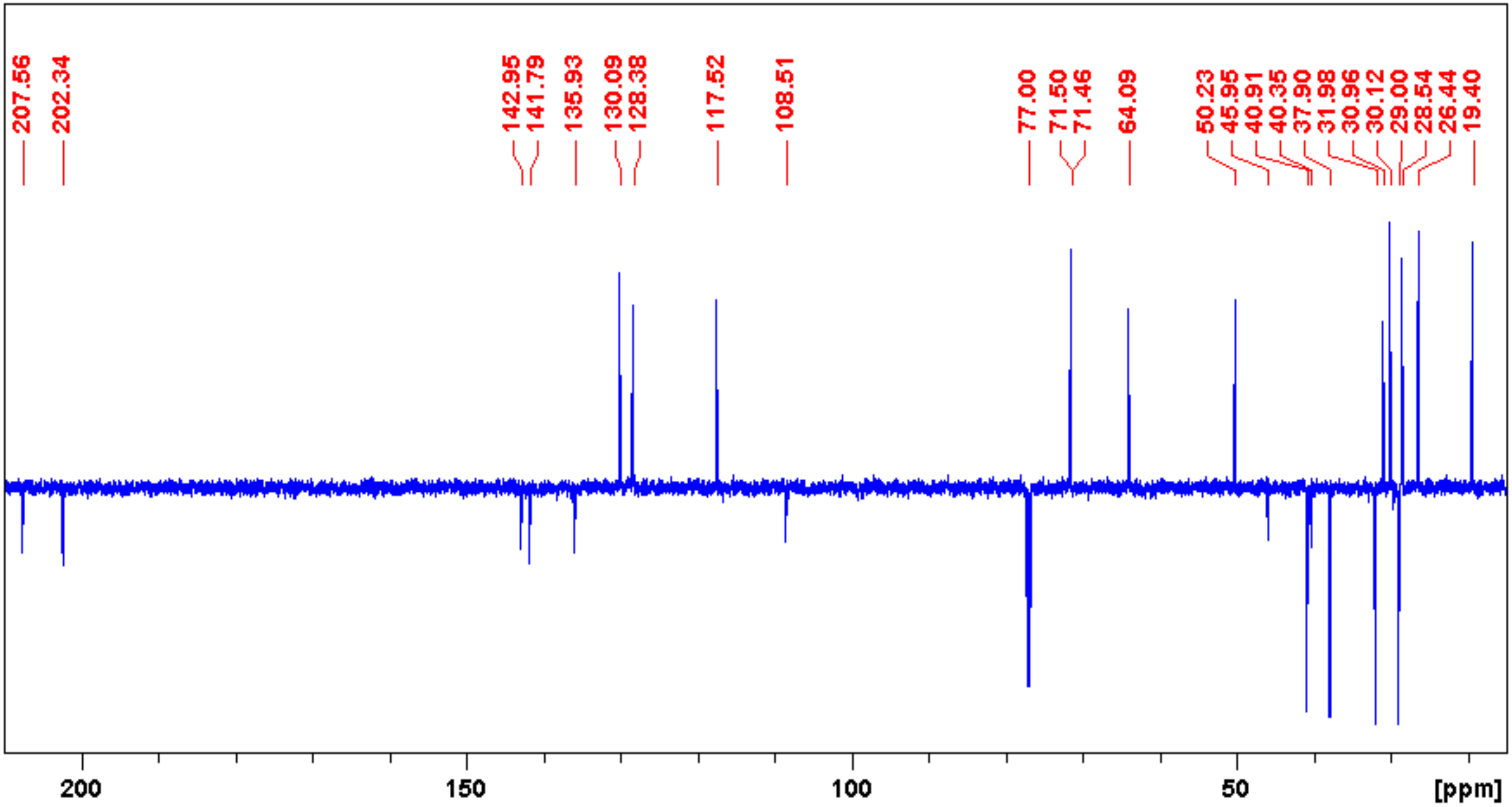

Figure S14. Compound **6**, edHSQC and edHSQC CH_2 section.

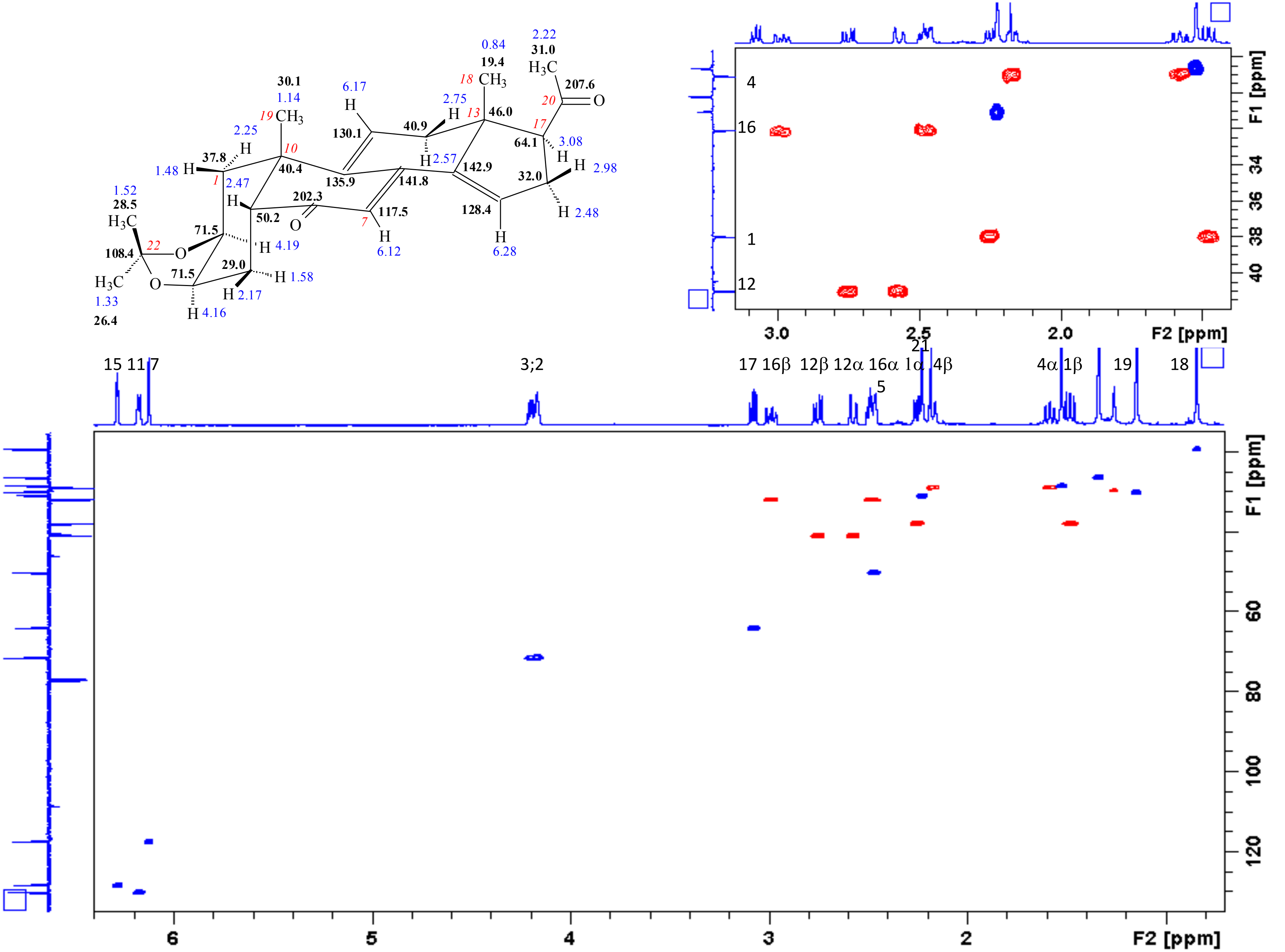

Figure S15. Compound **6**, HMBC and HMBC CH_3 section.

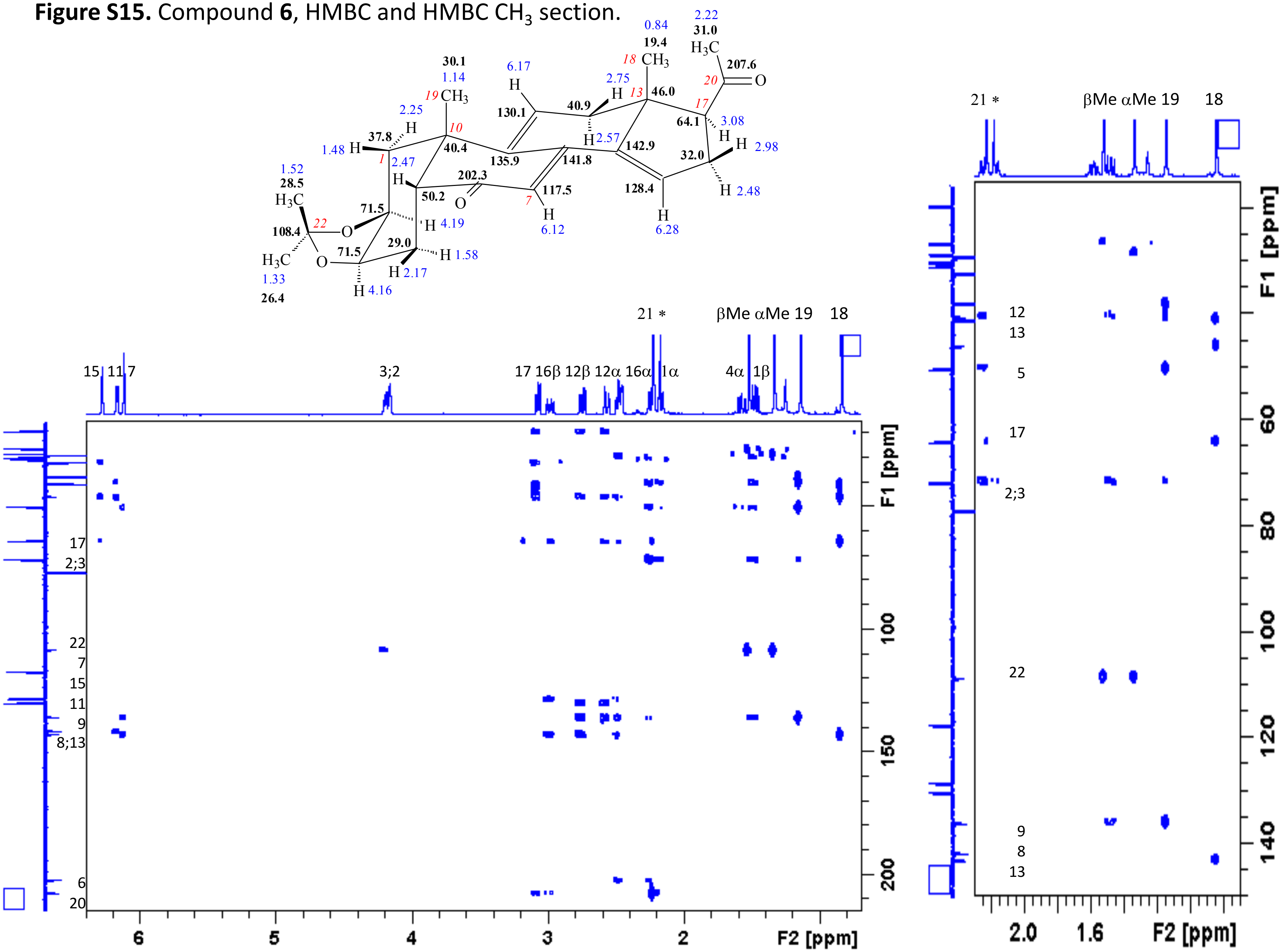

Figure S16. Compound **7**, ^{1}H NMR $CDCl_3$ 500 MHz and steric proximities detected by selROESY on signals H_3-19 and H_3-18.

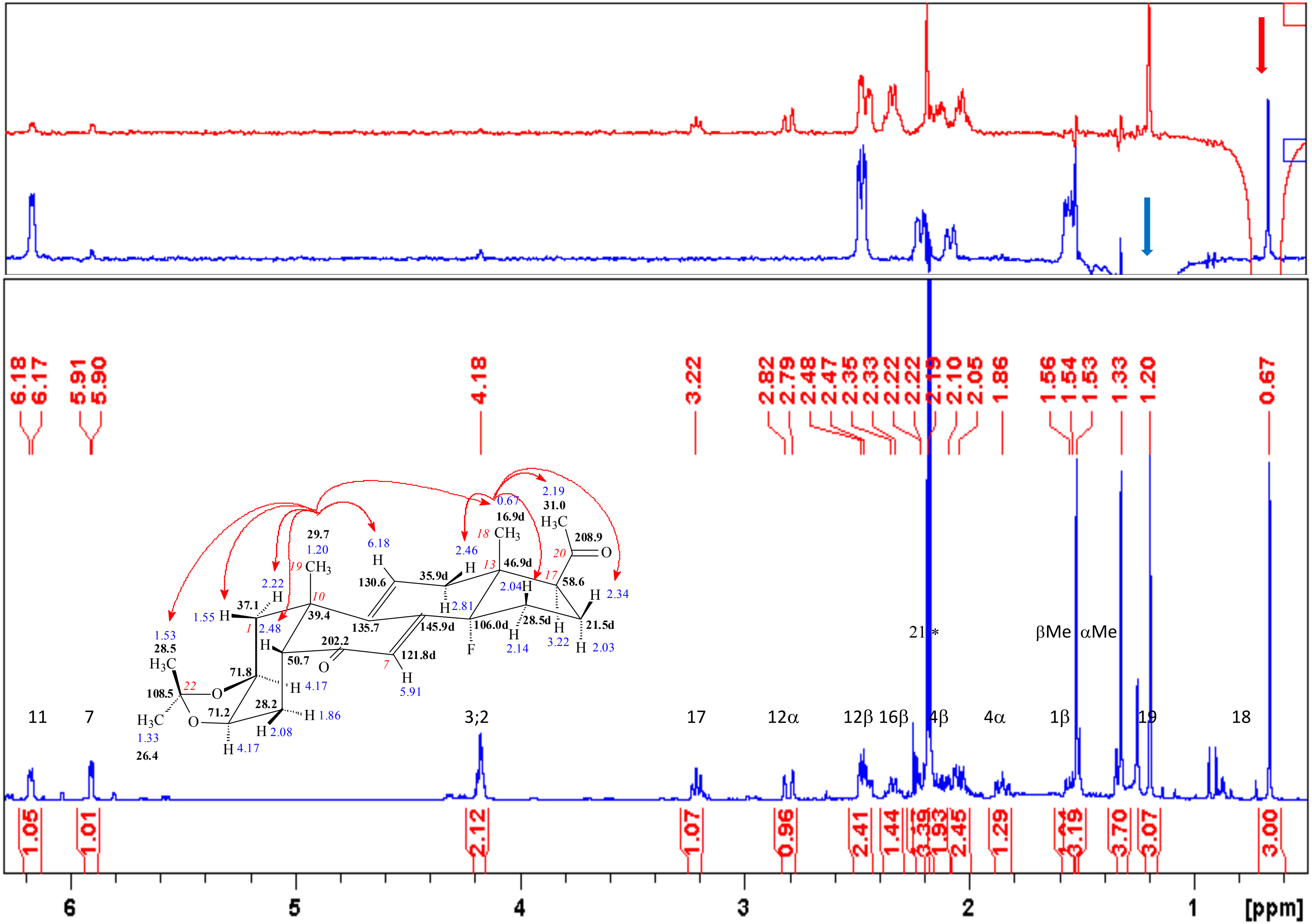

Figure S17. Compound **7**, DEPTQ 125 MHz.

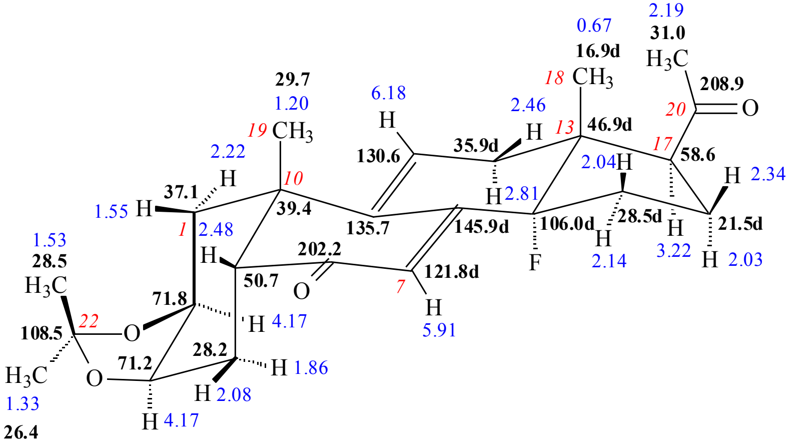

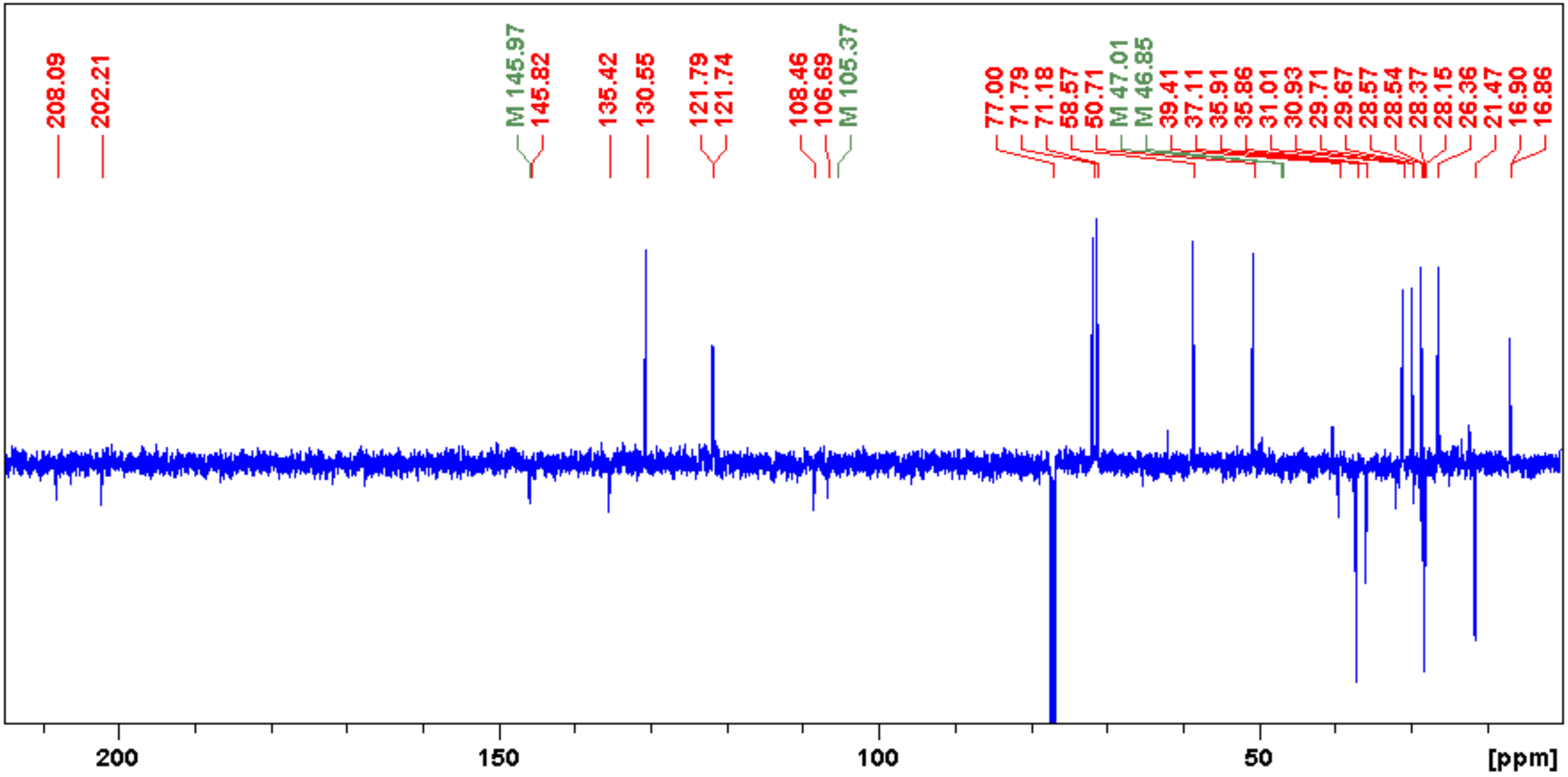

Figure S18. Compound **7**, edHSQC and edHSQC CH$_2$ section.

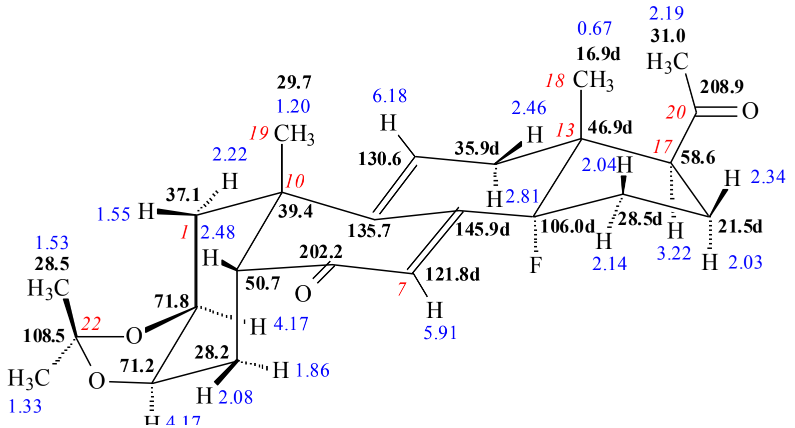

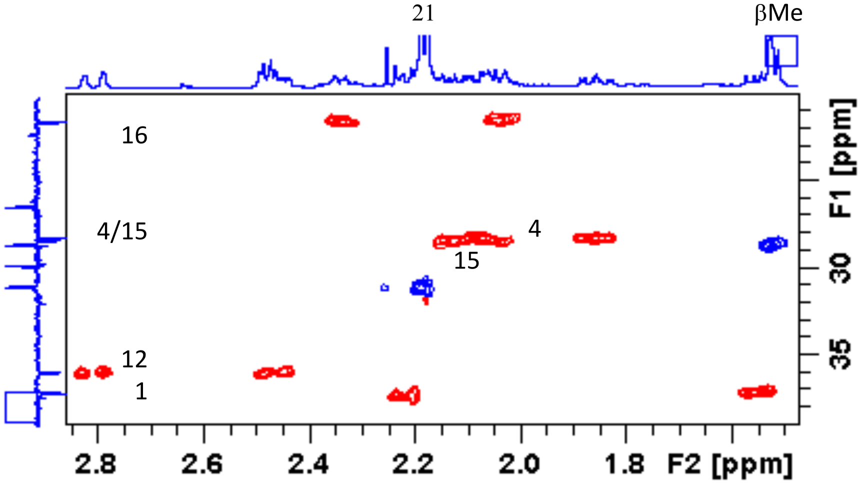

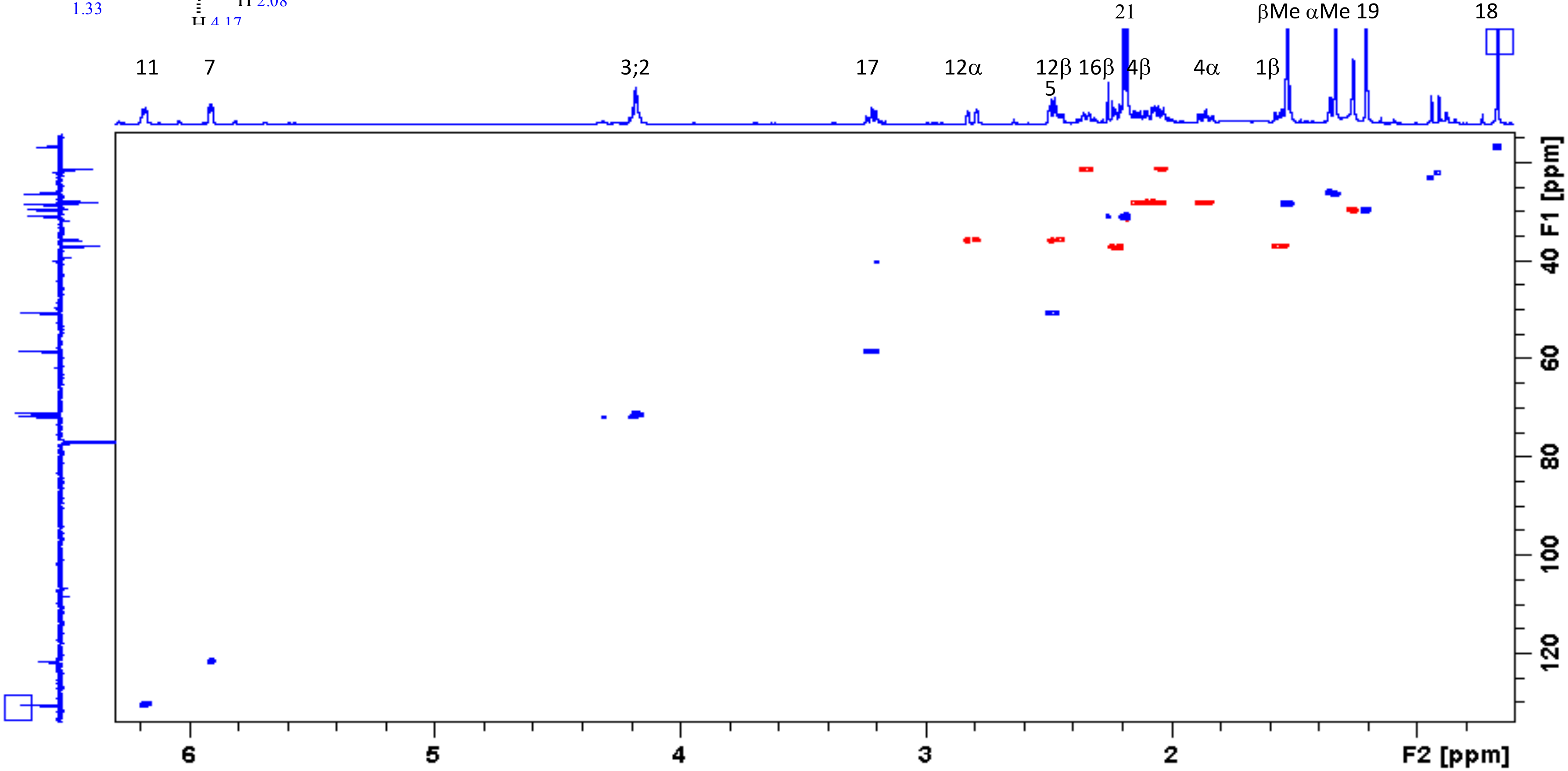

Figure S19. Compound **7**, HMBC and HMBC CH_3 section.

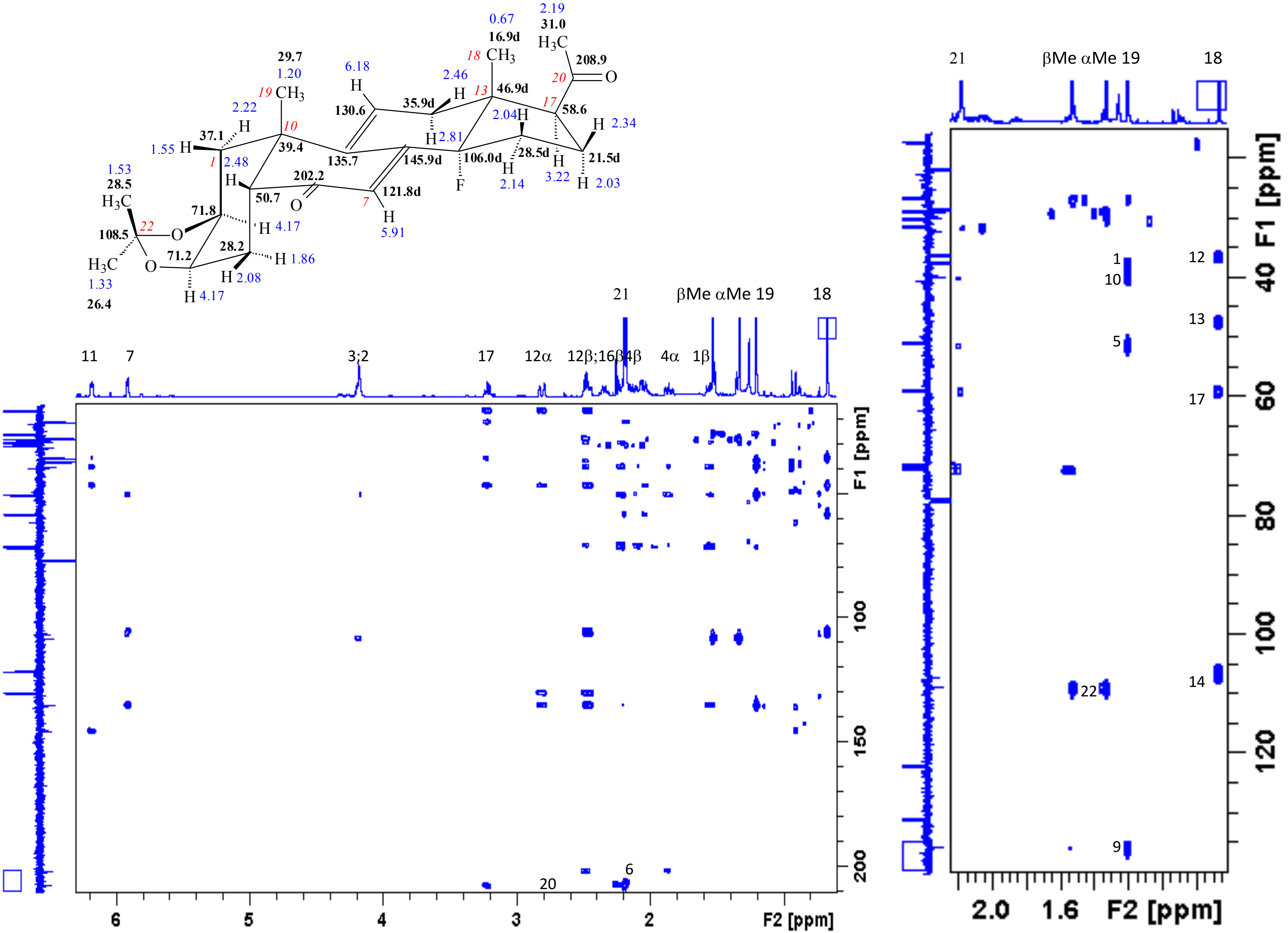

Figure S20. Compound **10**, ^{1}H NMR $CDCl_3$ 600 MHz and selTOCSY on H-15 and Hα-1.

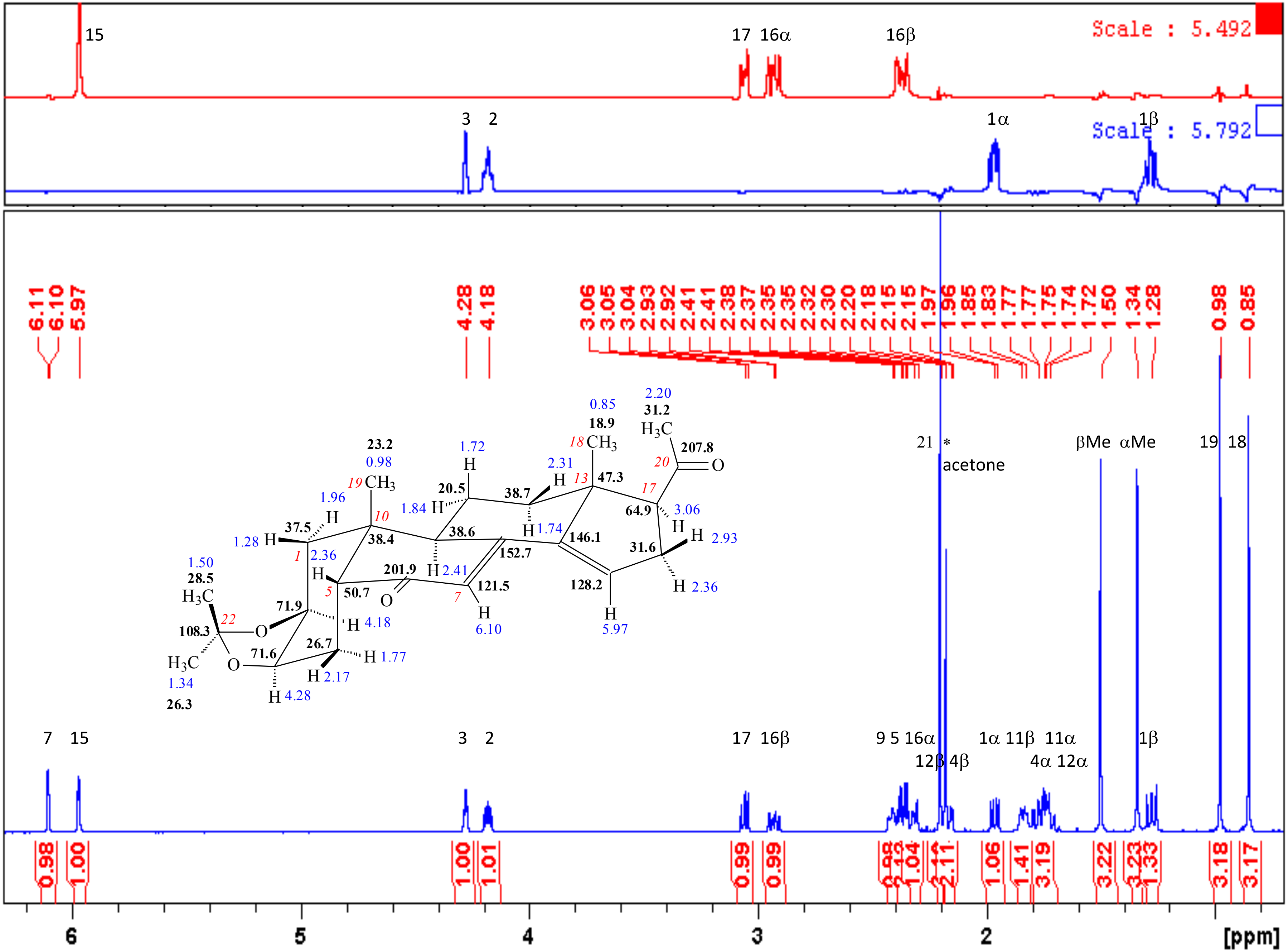

Figure S21. Compound **10**, steric proximities detected by selNOESY on signals βMe, H_3-19 and H_3-18.

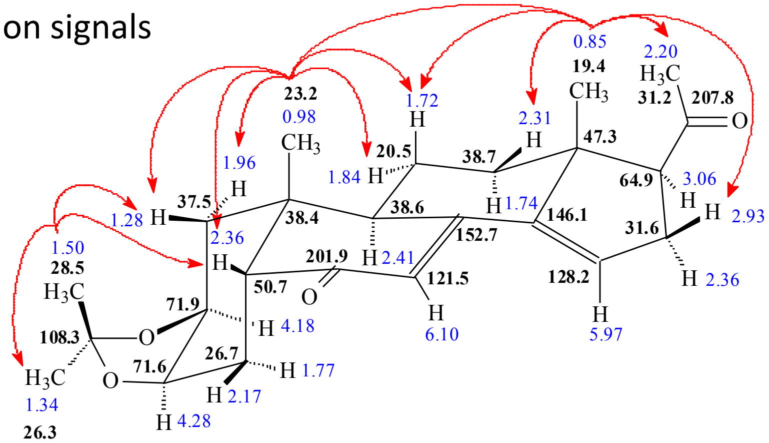

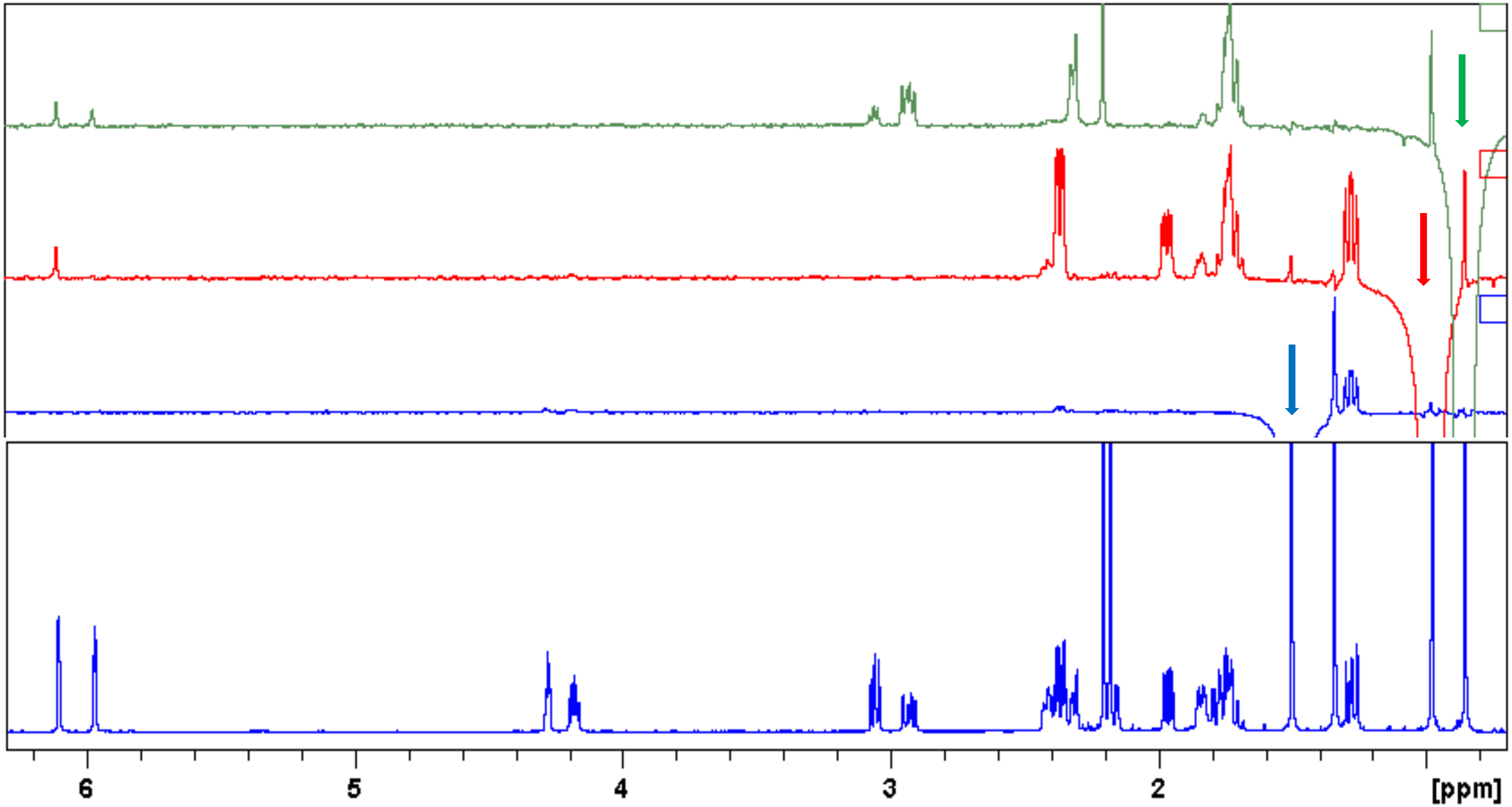

Figure S22. Compound **10**, DEPTQ 150 MHz.

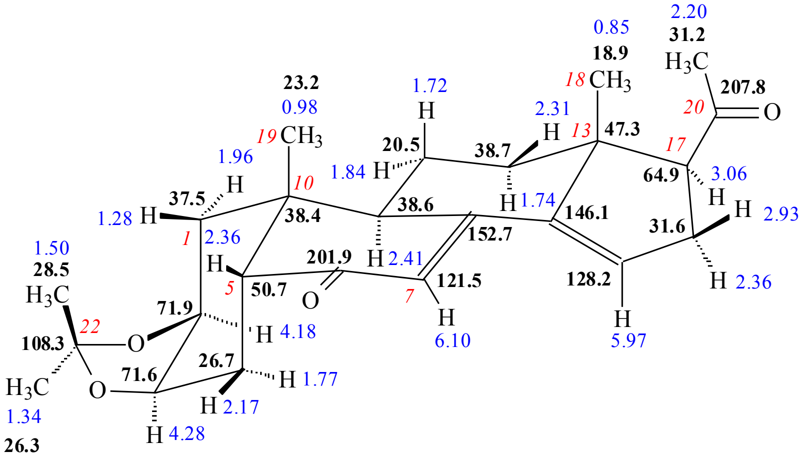

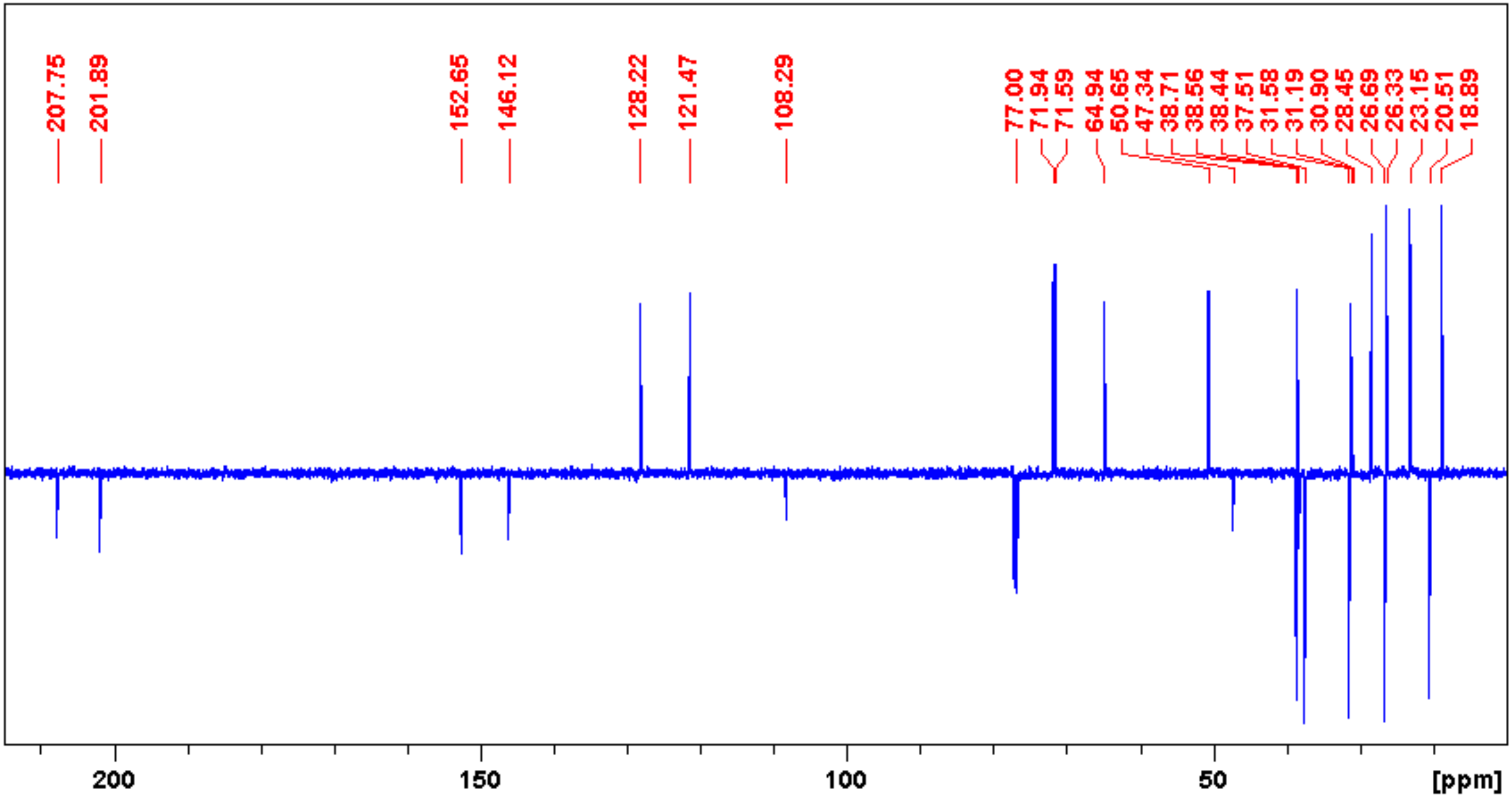

Figure S23. Compound **10**, edHSQC.

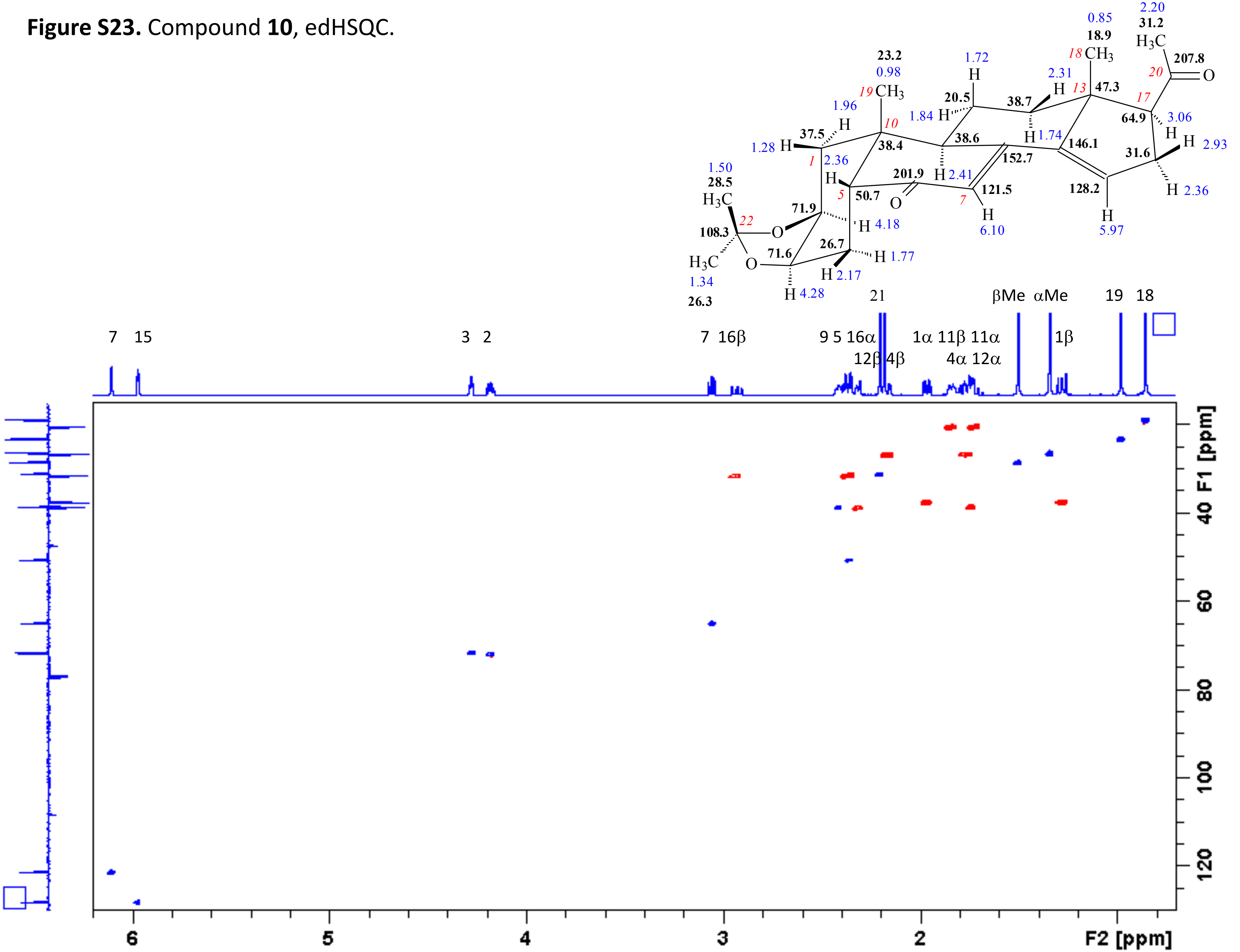

Figure S24. Compound **10**, HMBC.

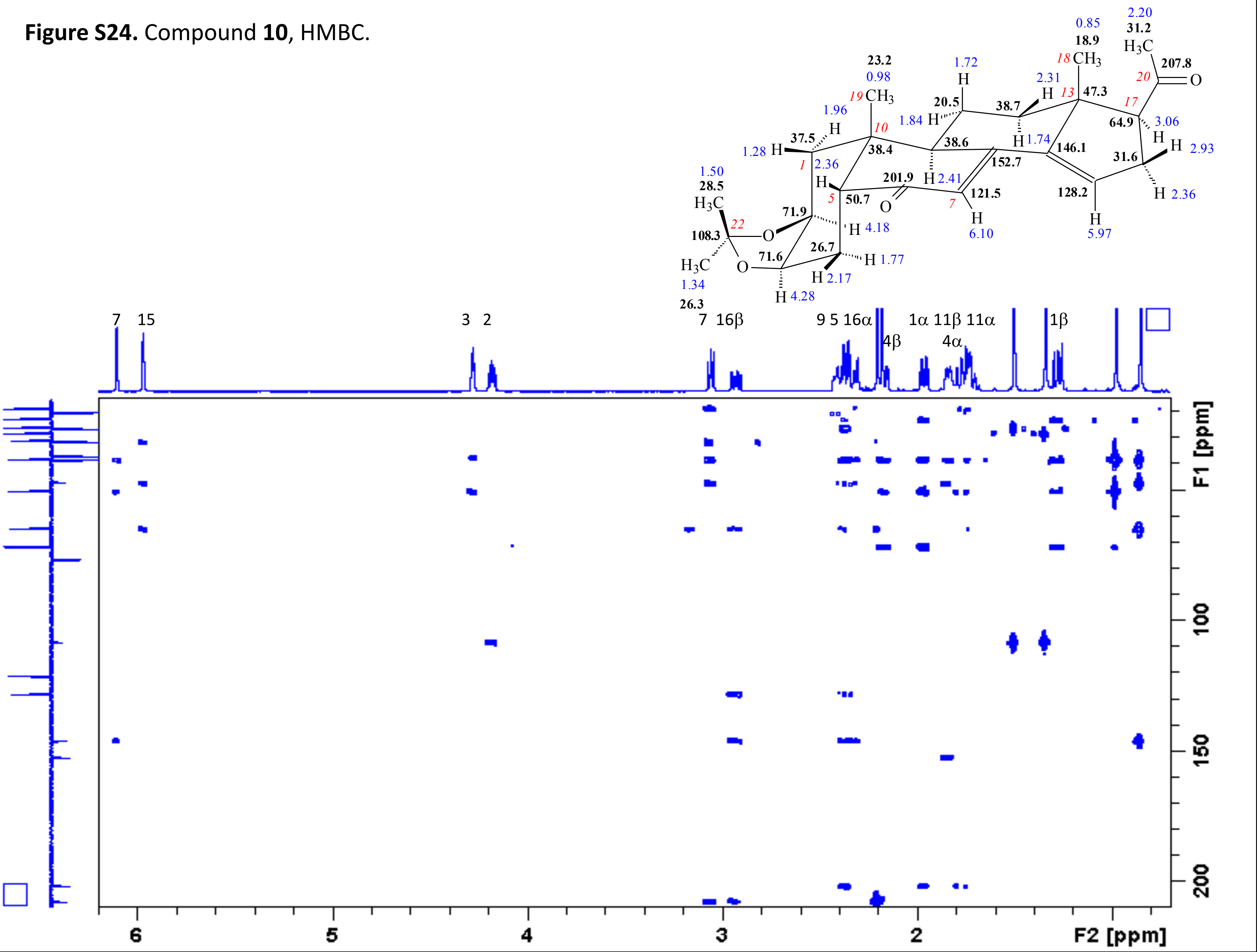

Figure S25. Compound **10**, edHSQC CH_2 section and HMBC CH_3 section.

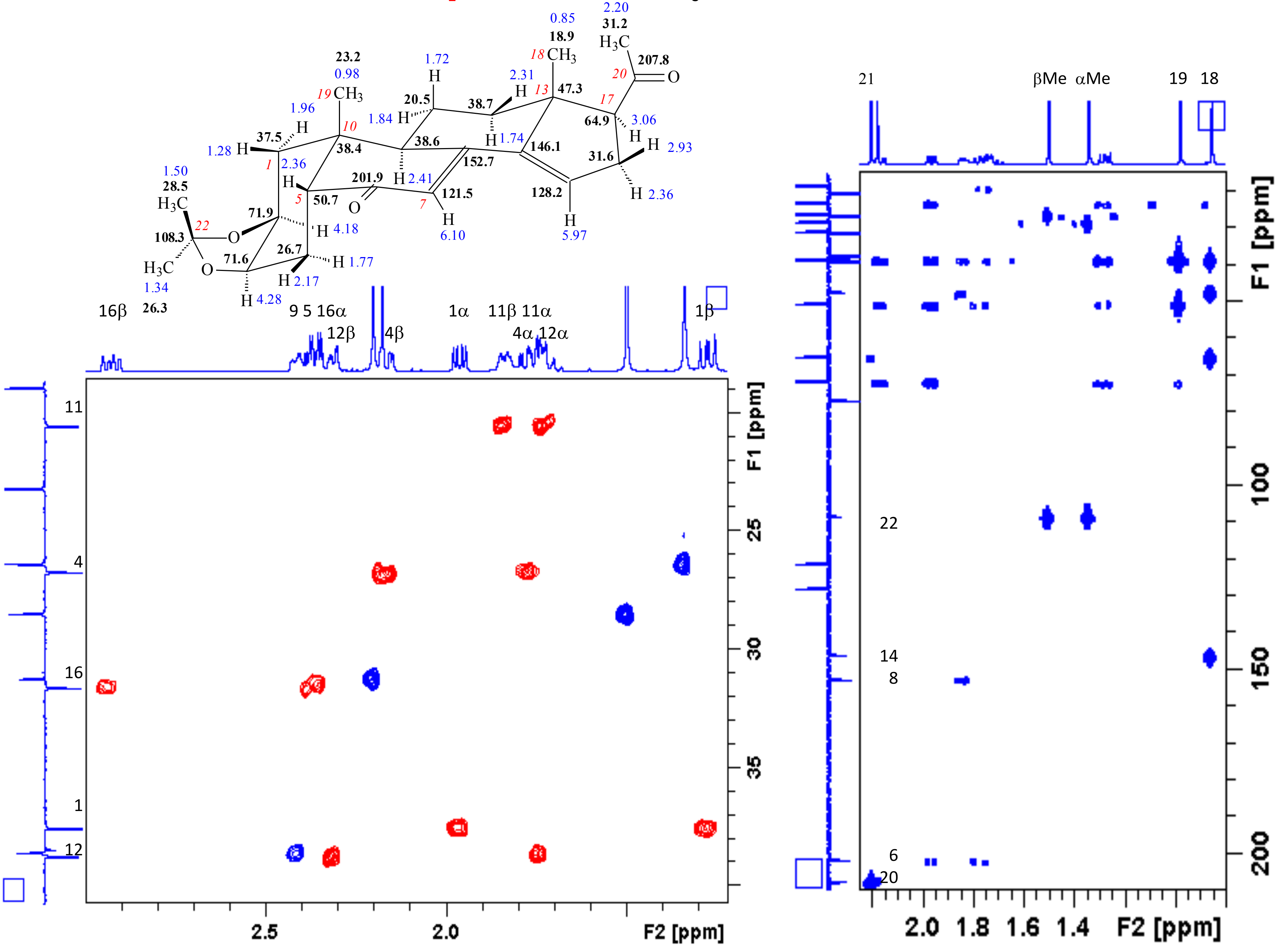

Figure S26. Compound **11**, ^{1}H NMR $CDCl_3$ 600 MHz.

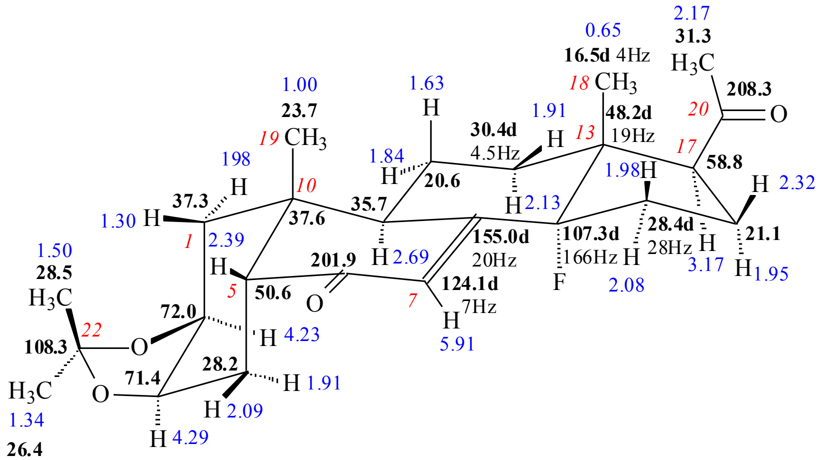

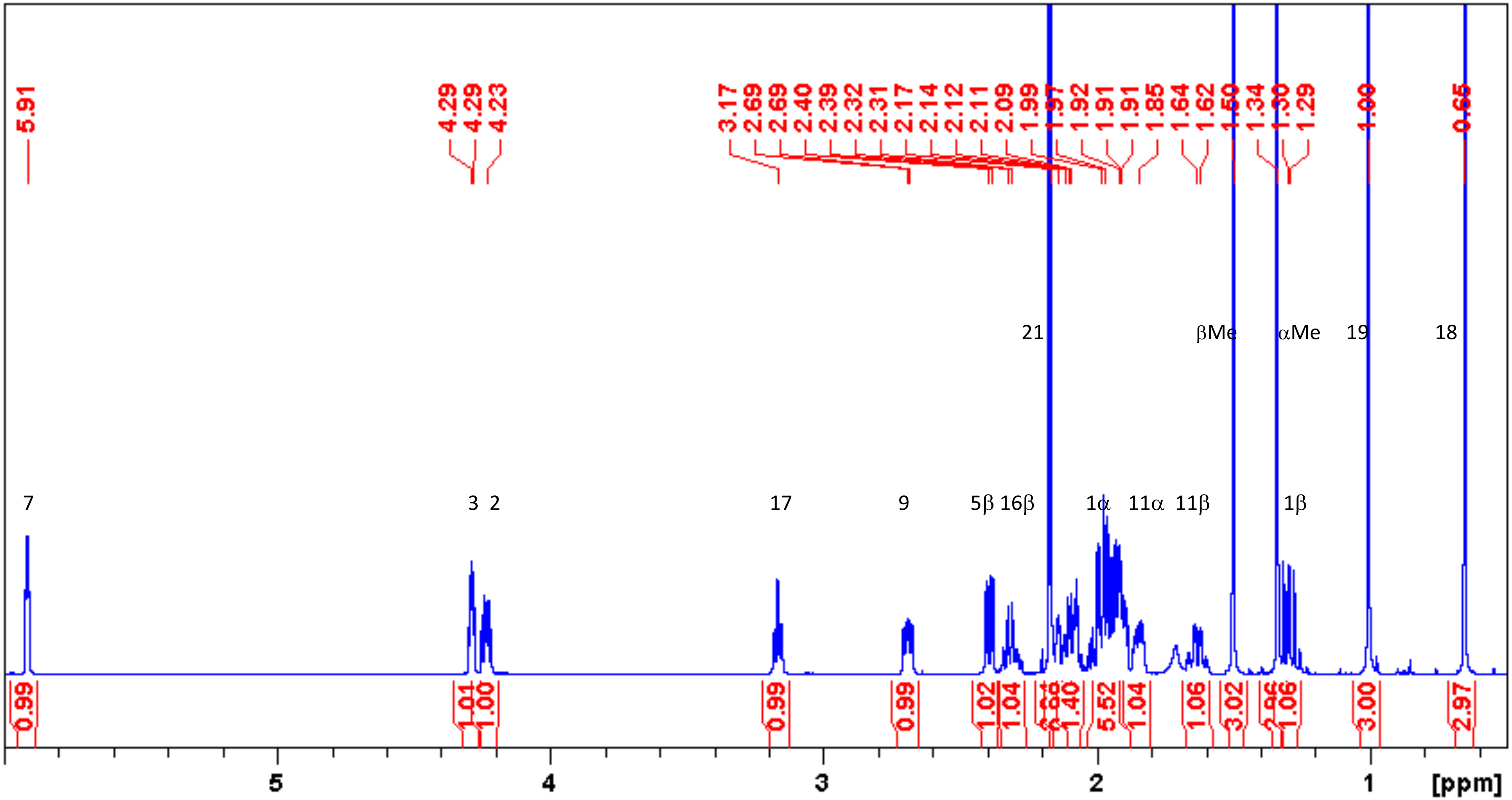

Figure S27. Compound **11**, steric proximities detected by selNOESY on signals βMe, H_3-19 and H_3-18.

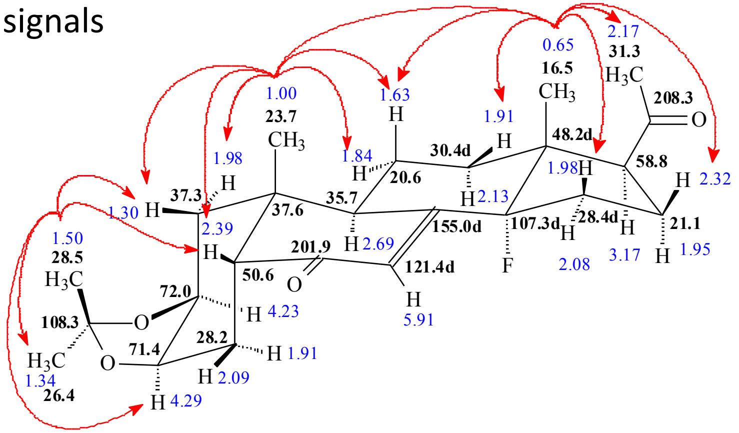

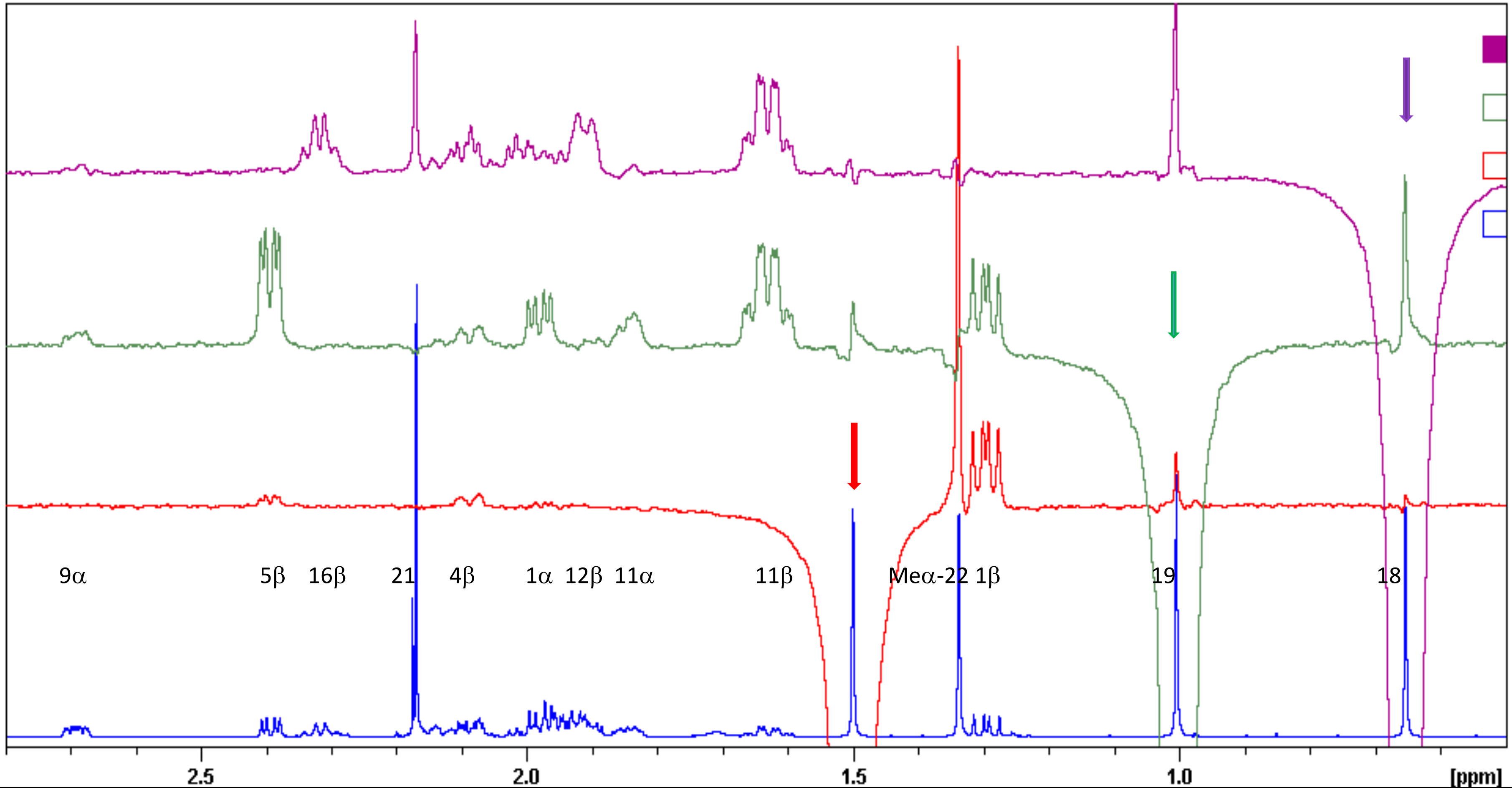

Figure S28. Compound **11**, DEPTQ 150 MHz.

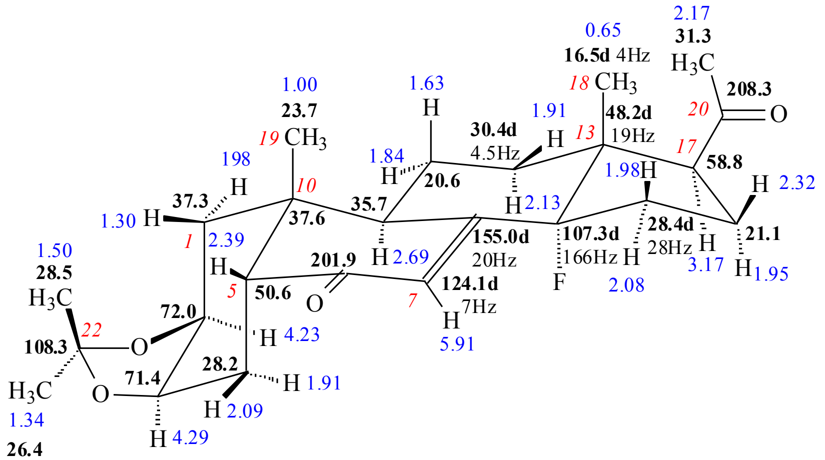

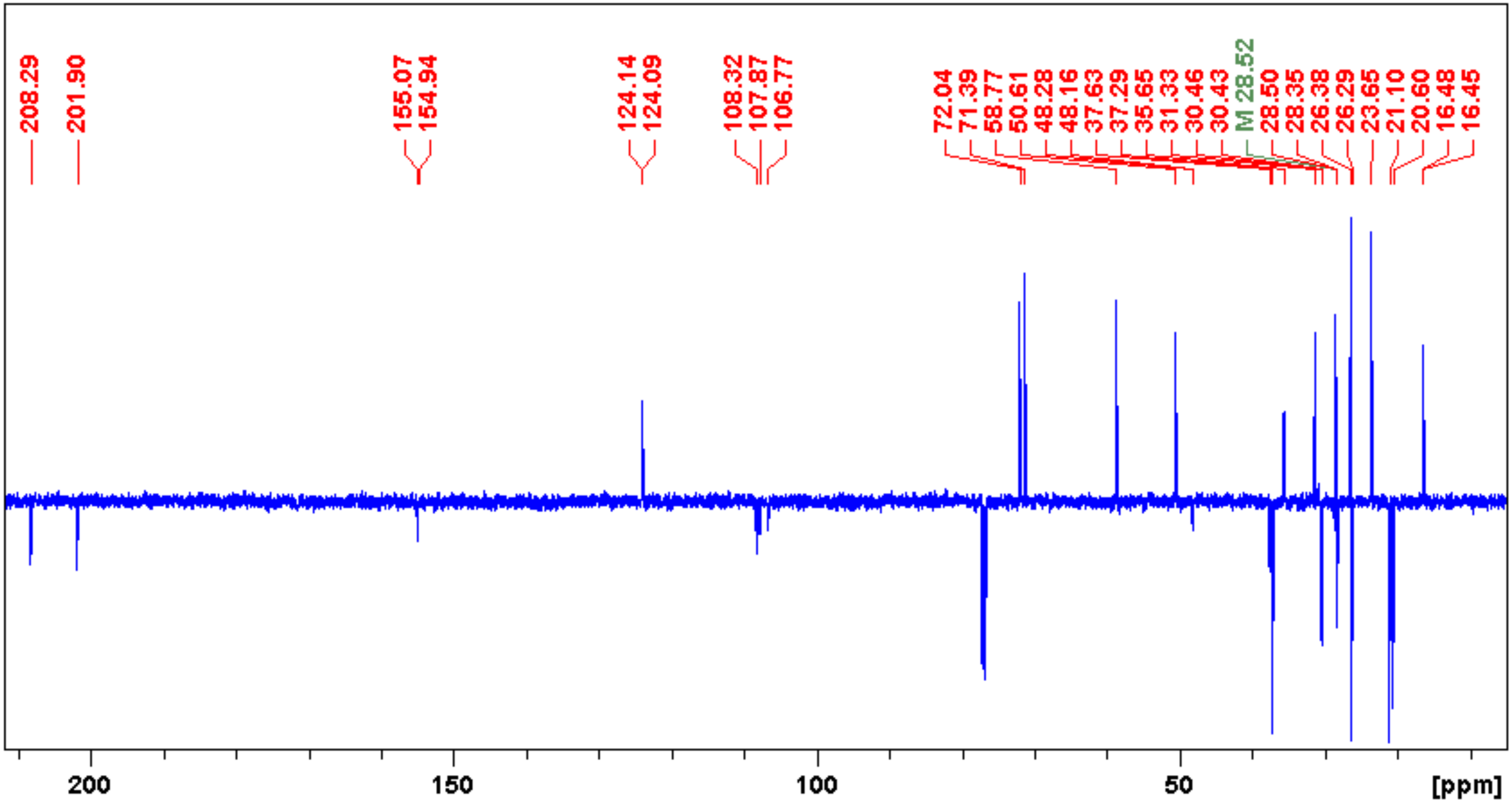

Figure S29. Compound **11**, edHSQC.

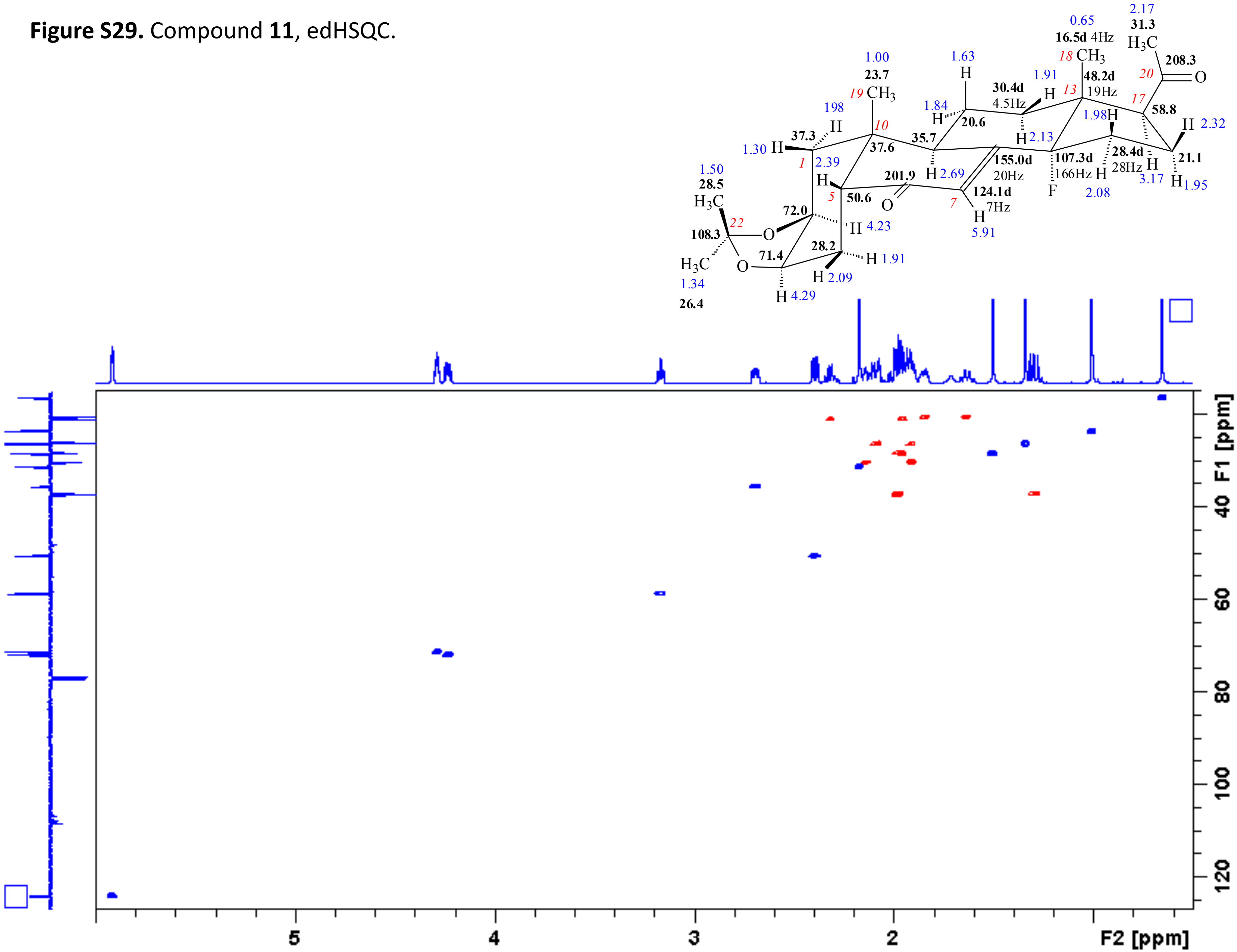

Figure S30. Compound **11**, HMBC.

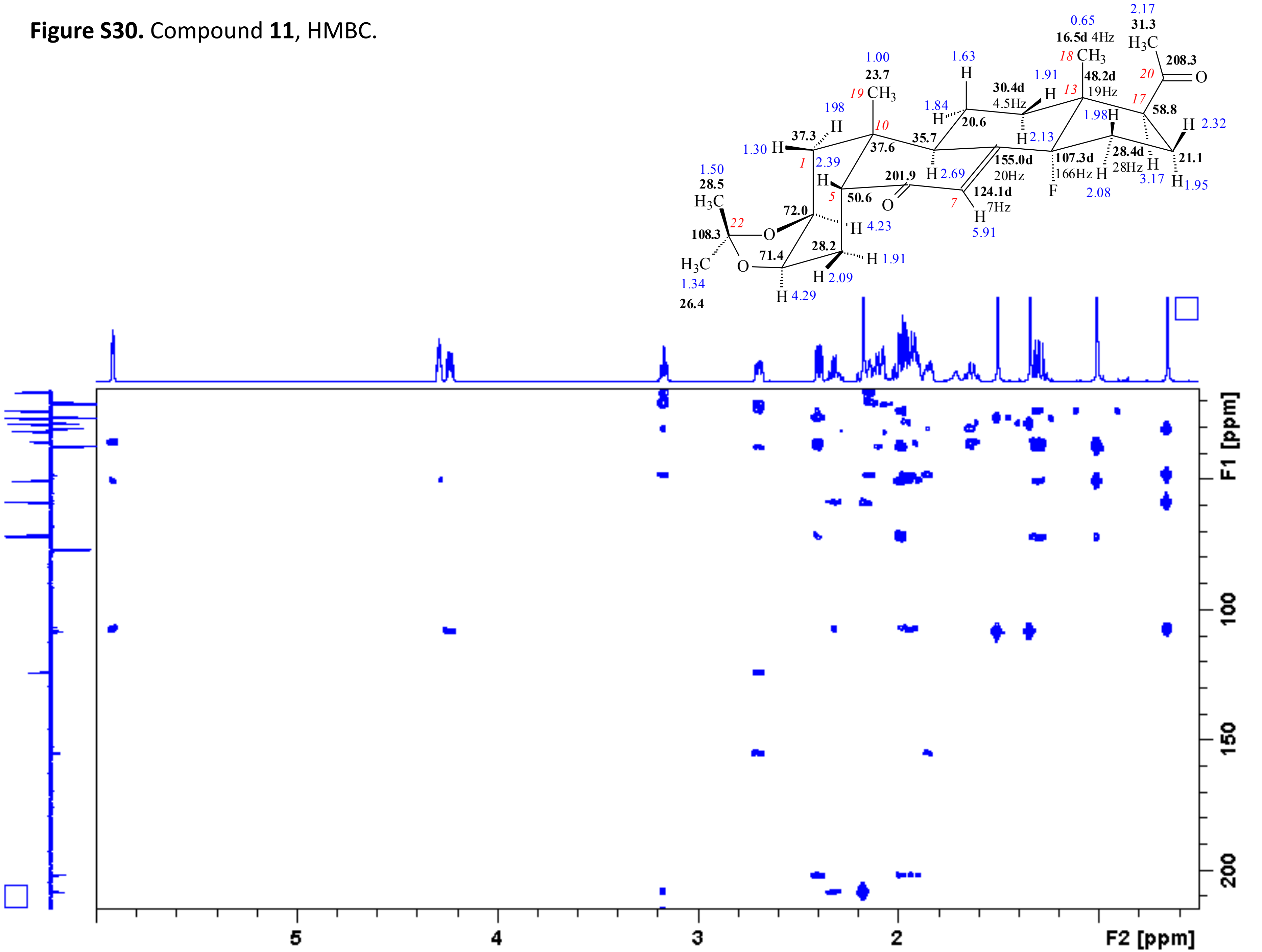

Figure S31. Compound **11**, edHSQC CH_2 section and HMBC CH_3 section.

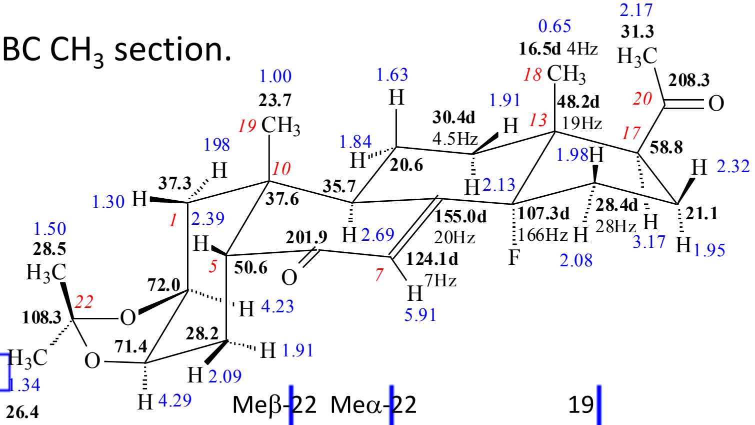

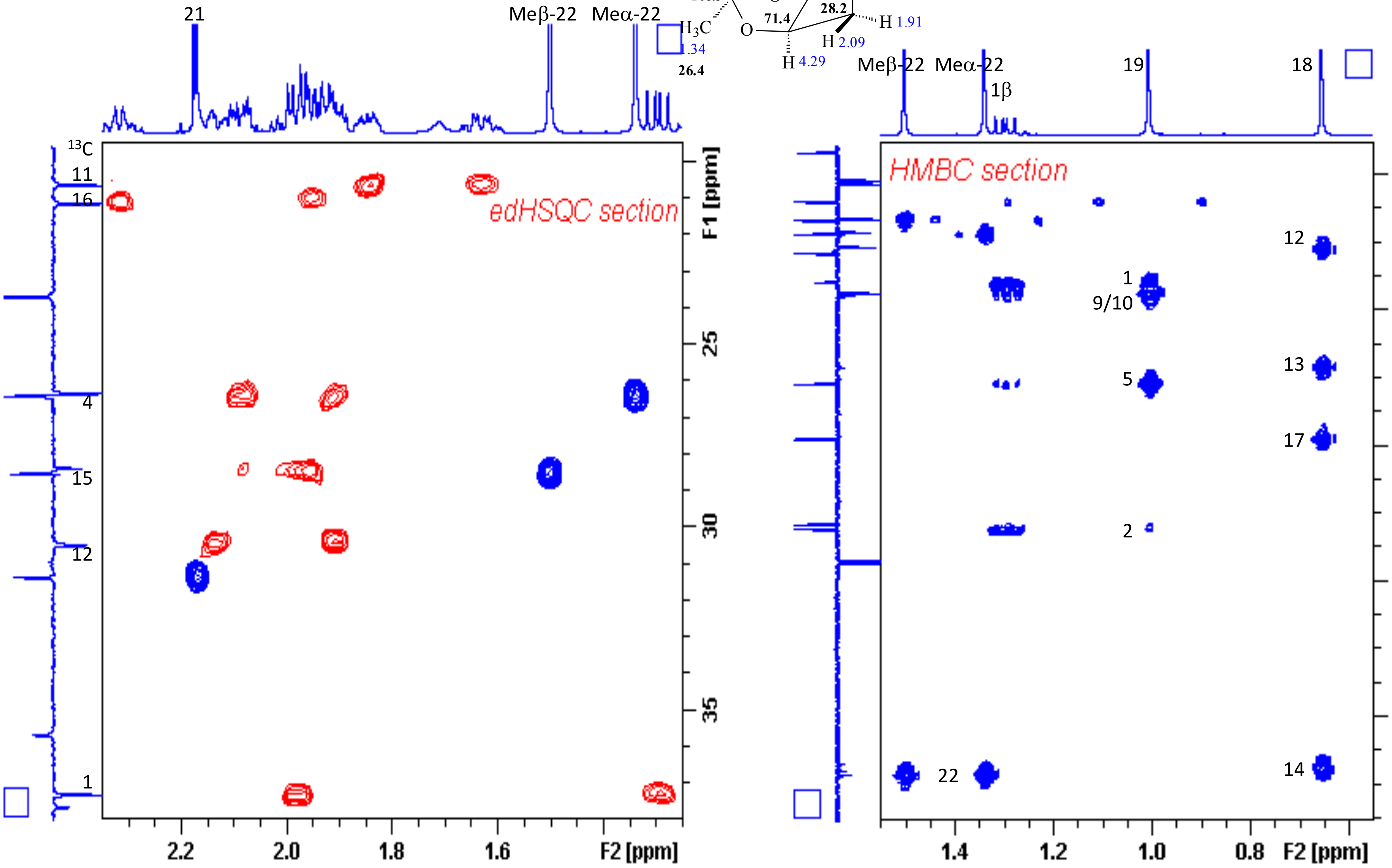

Figure S32. Compound **13**, ^{1}H NMR $CDCl_3$ 600 MHz and selTOCSY on H-17 and H-3.

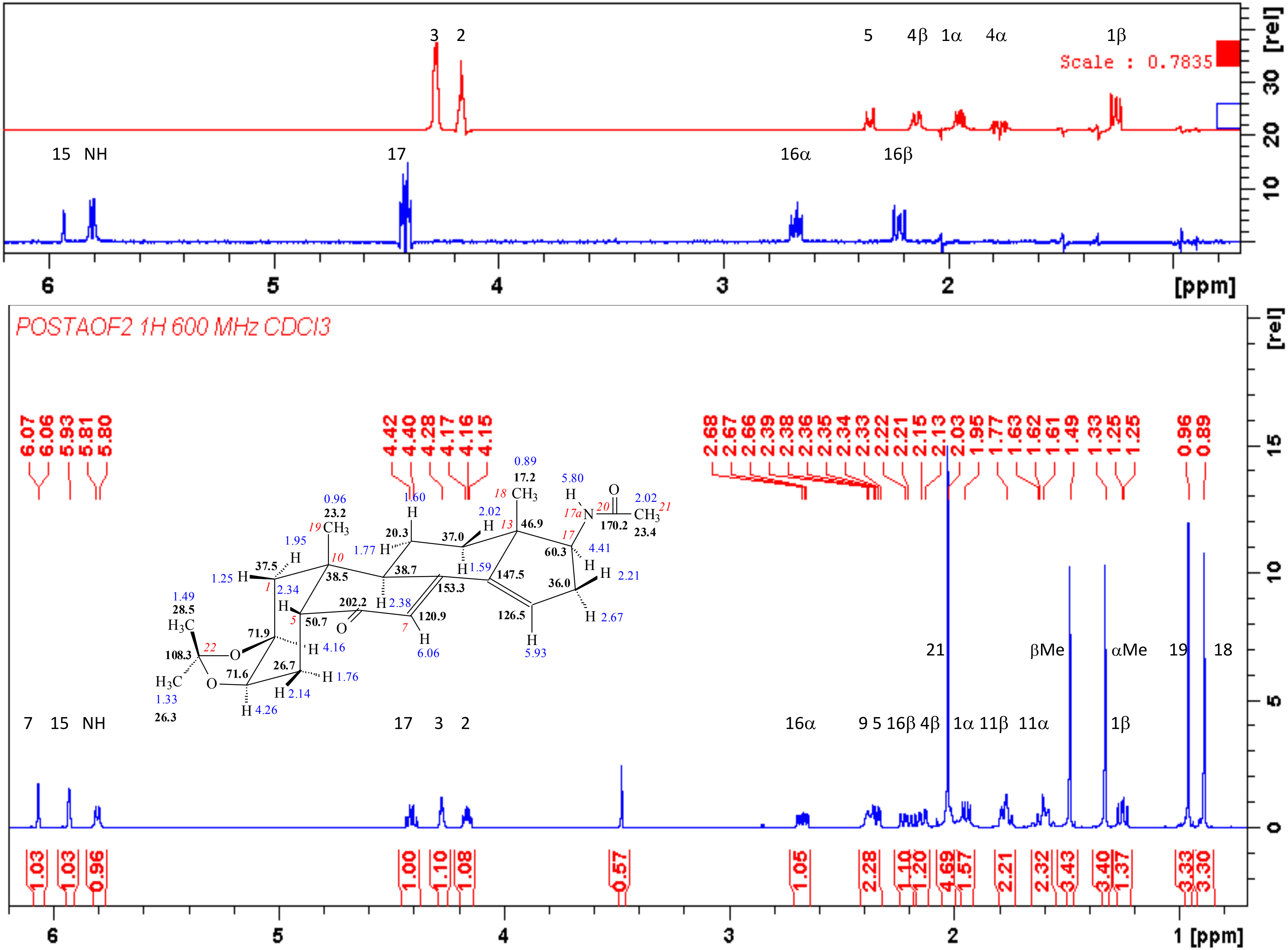

Figure S33. Compound **13**, steric proximities detected by selROESY on signals H_3-19 and H_3-18.

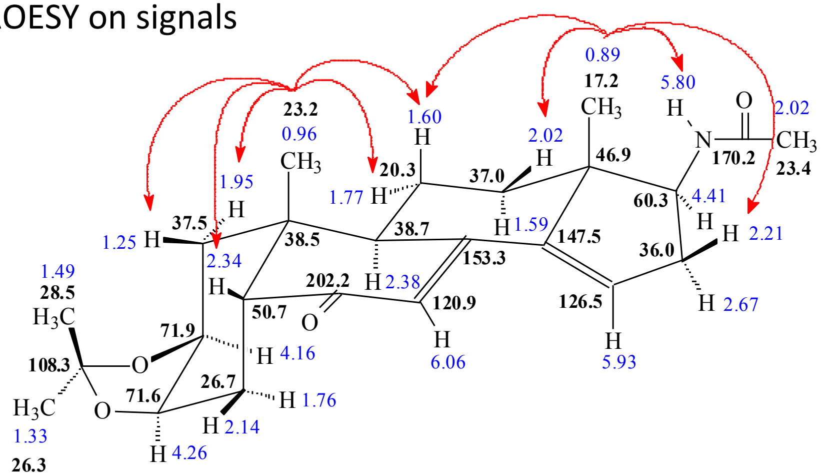

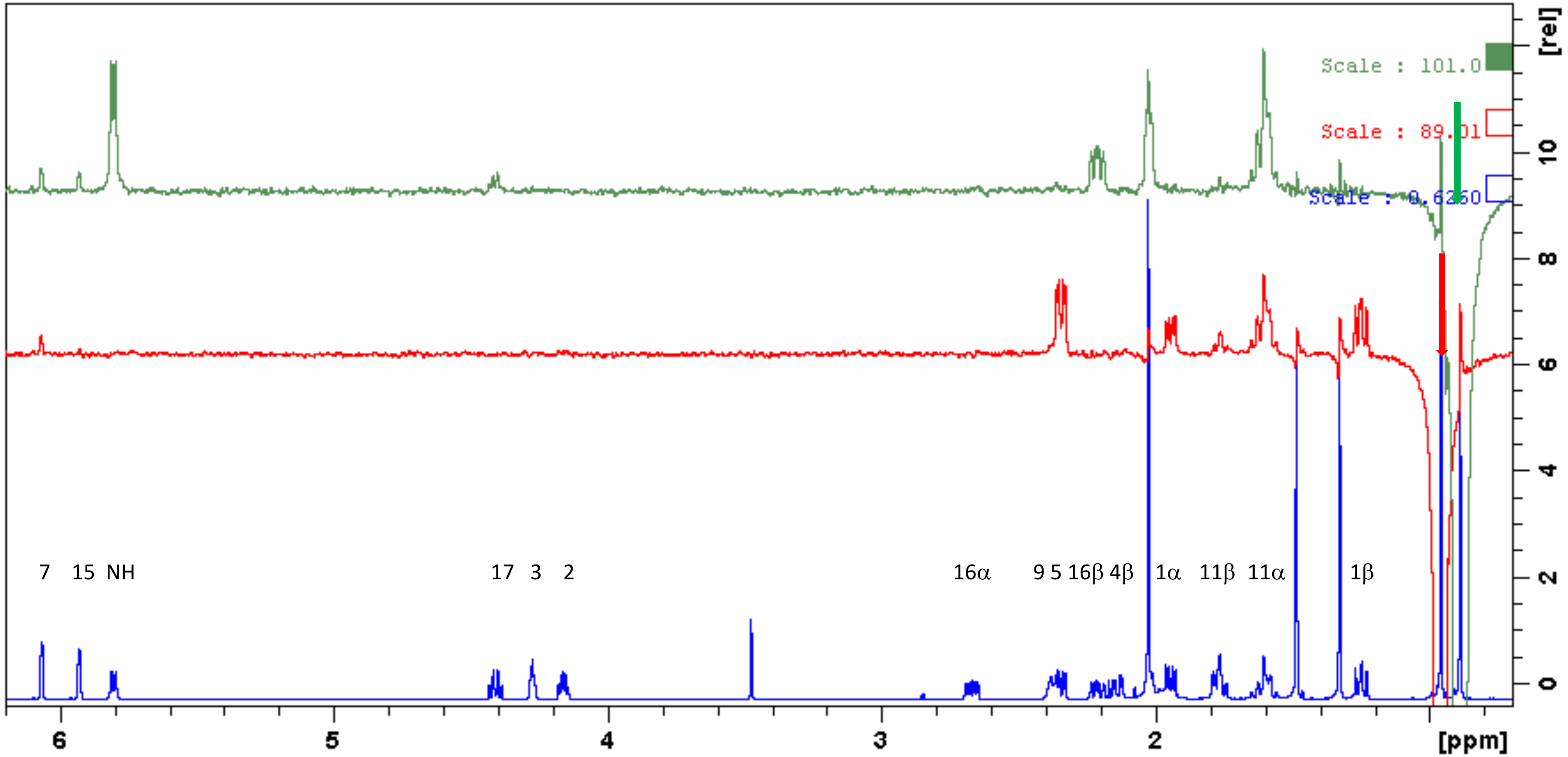

Figure S34. Compound **13**, DEPTQ 150 MHz.

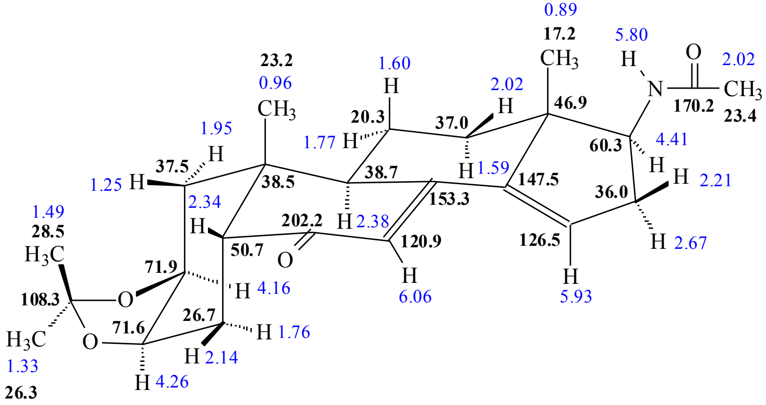

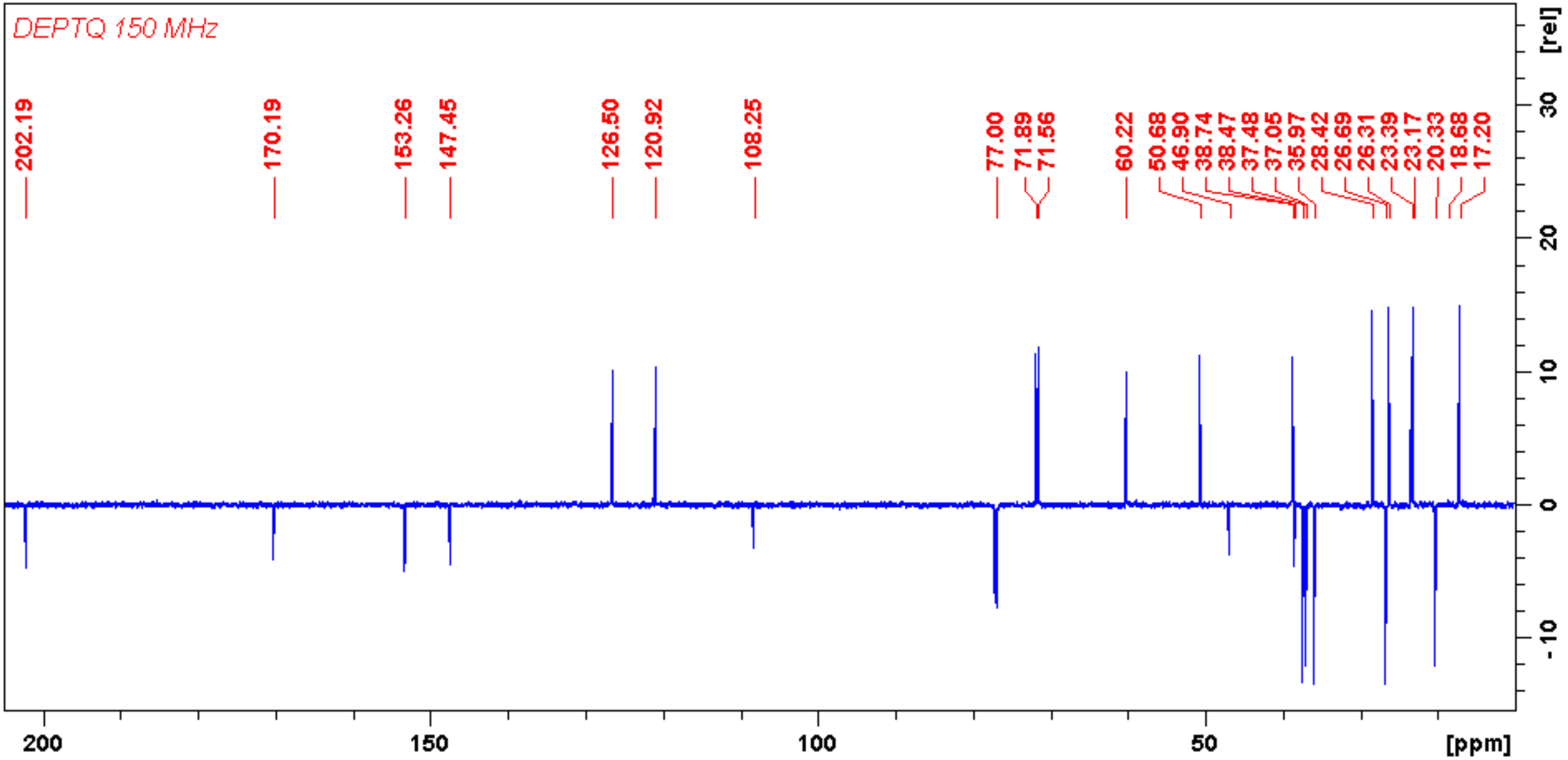

Figure S35. Compound **13**, edHSQC.

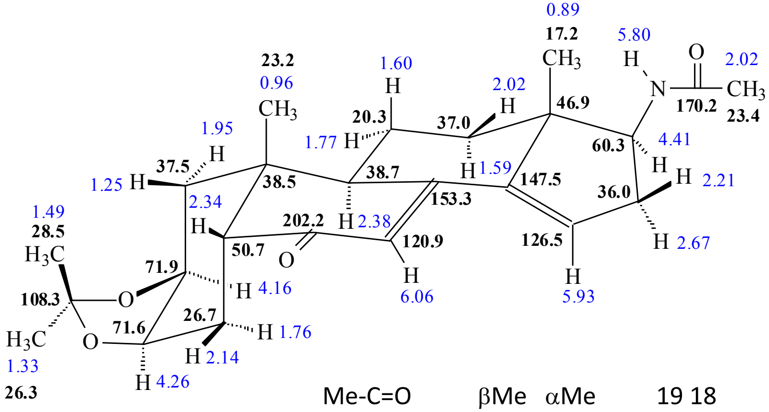

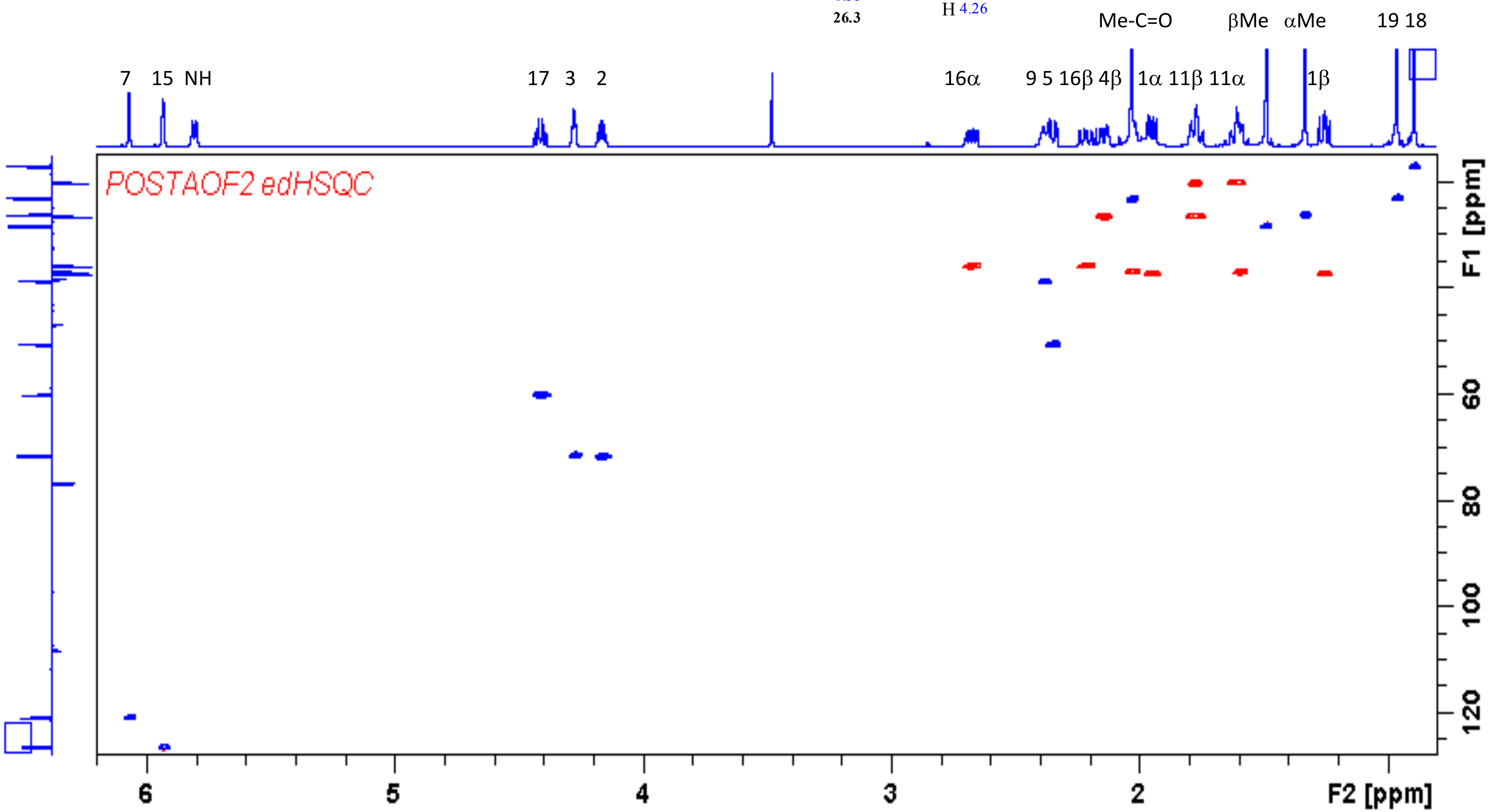

Figure S36. Compound **13**, HMBC.

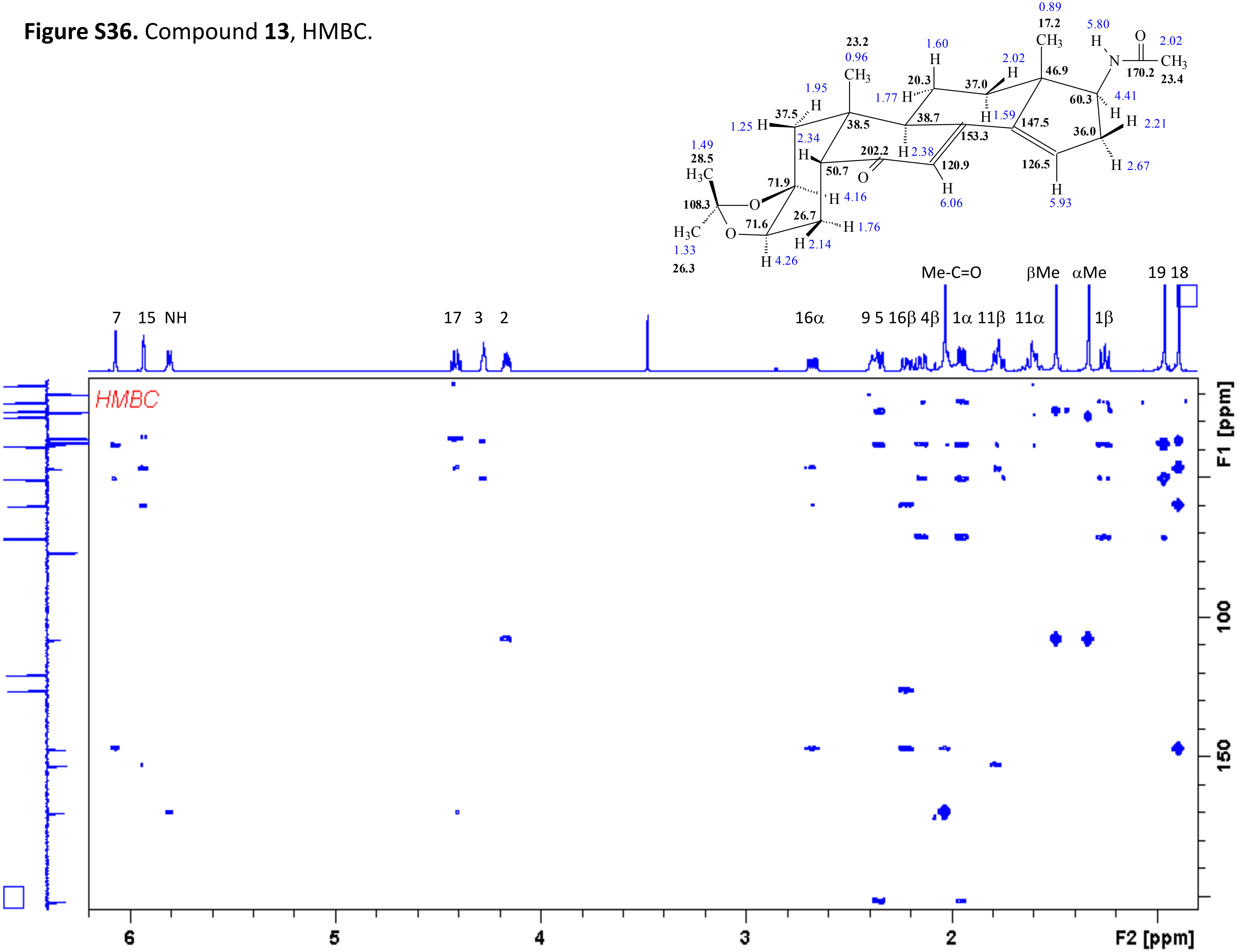

Figure S37. Compound **13**, edHSQC CH_2 section and HMBC CH_3 section.

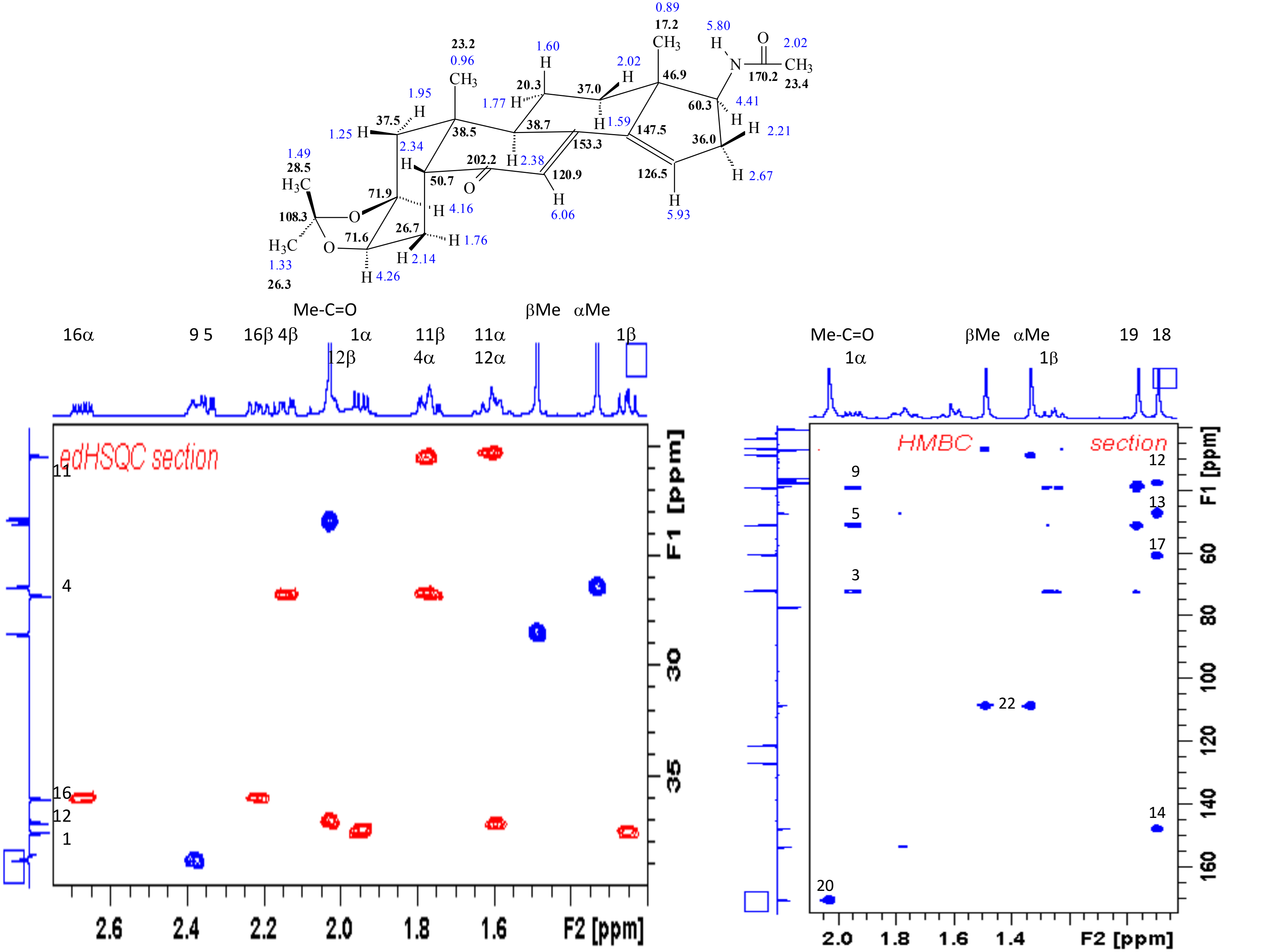

Figure S38. Compound **14**, ^{1}H $CDCl_3$ 600 MHz.

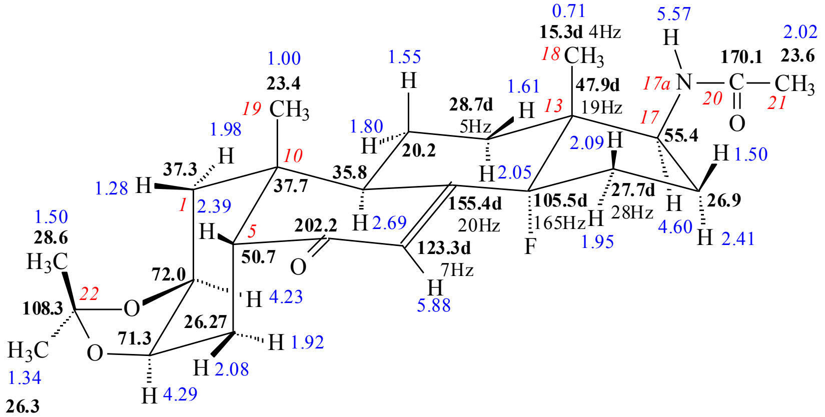

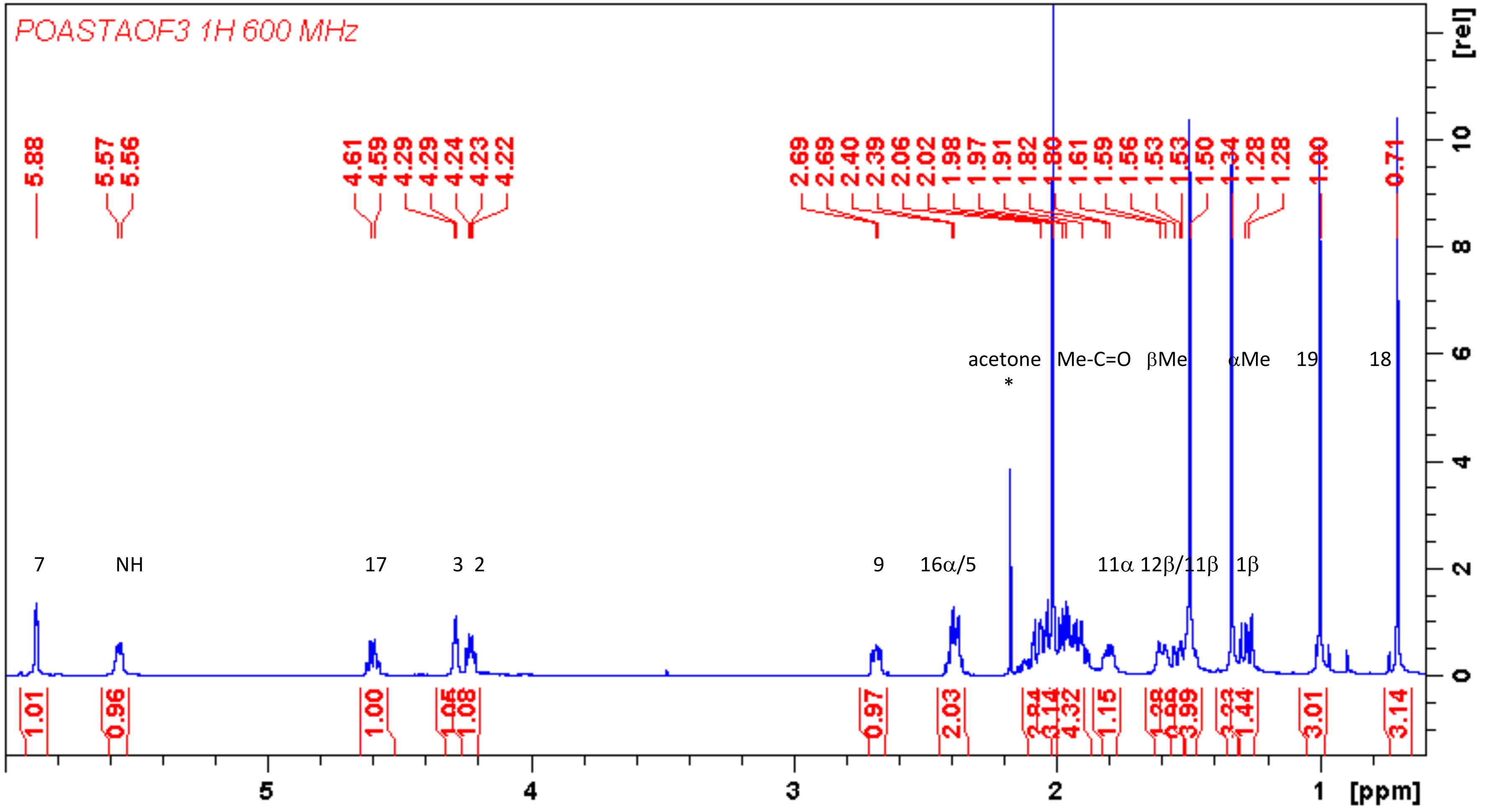

Figure S39. Compound **14**, identification of spin-systems of **A**, **C** and **D** rings by selTOCSY on H-3, H-9 and H-17.

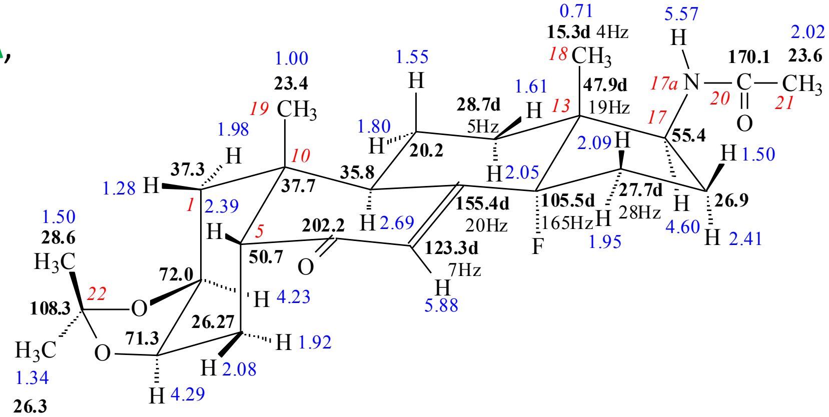

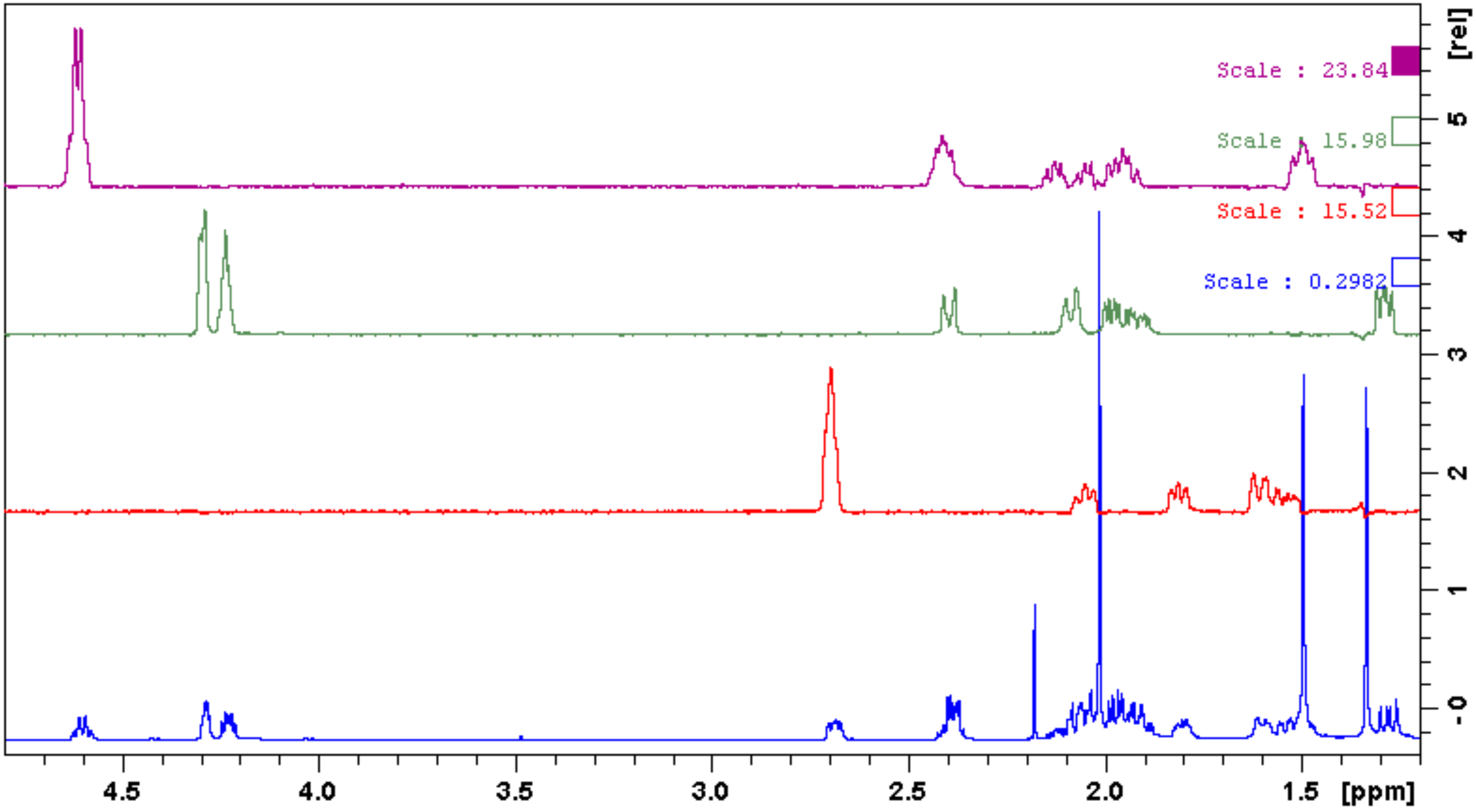

Figure S40. Compound **14**, steric proximities detected by selNOE on CH_3-19, CH_3-18 and NH signals.

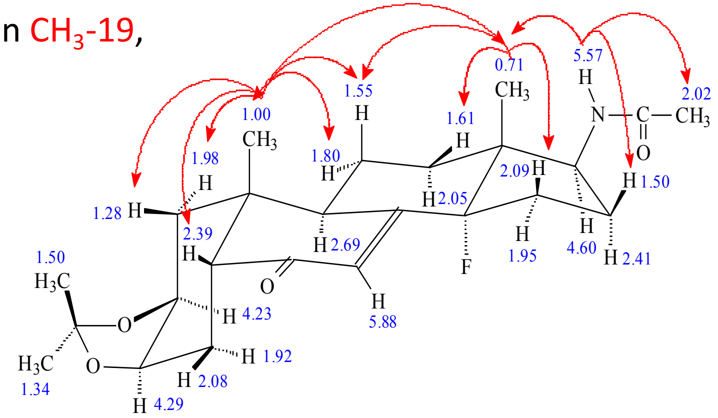

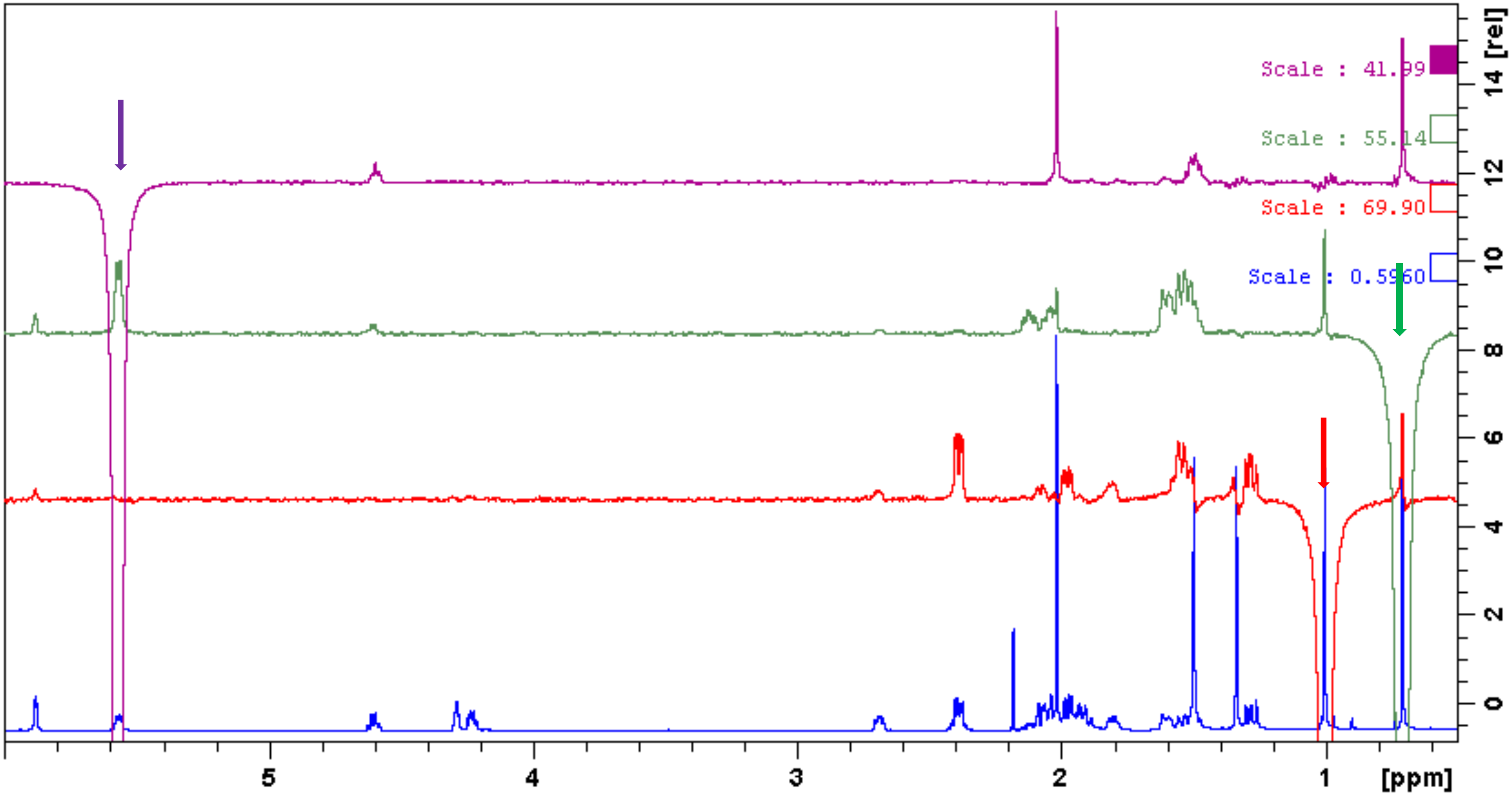

Figure 41. Compound **14**, DEPTQ 150 MHz.

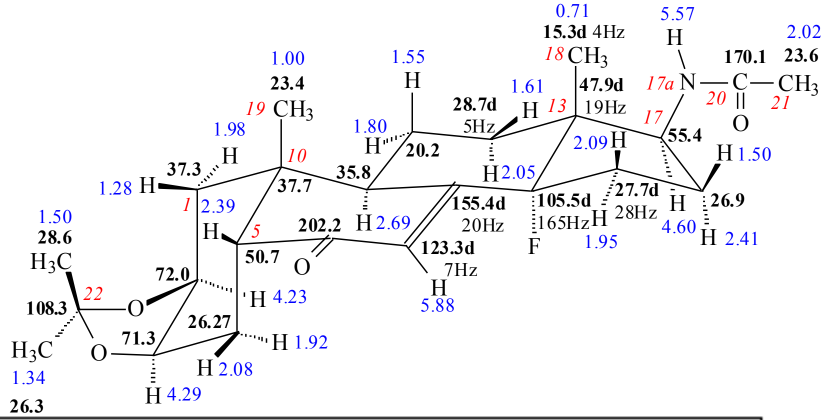

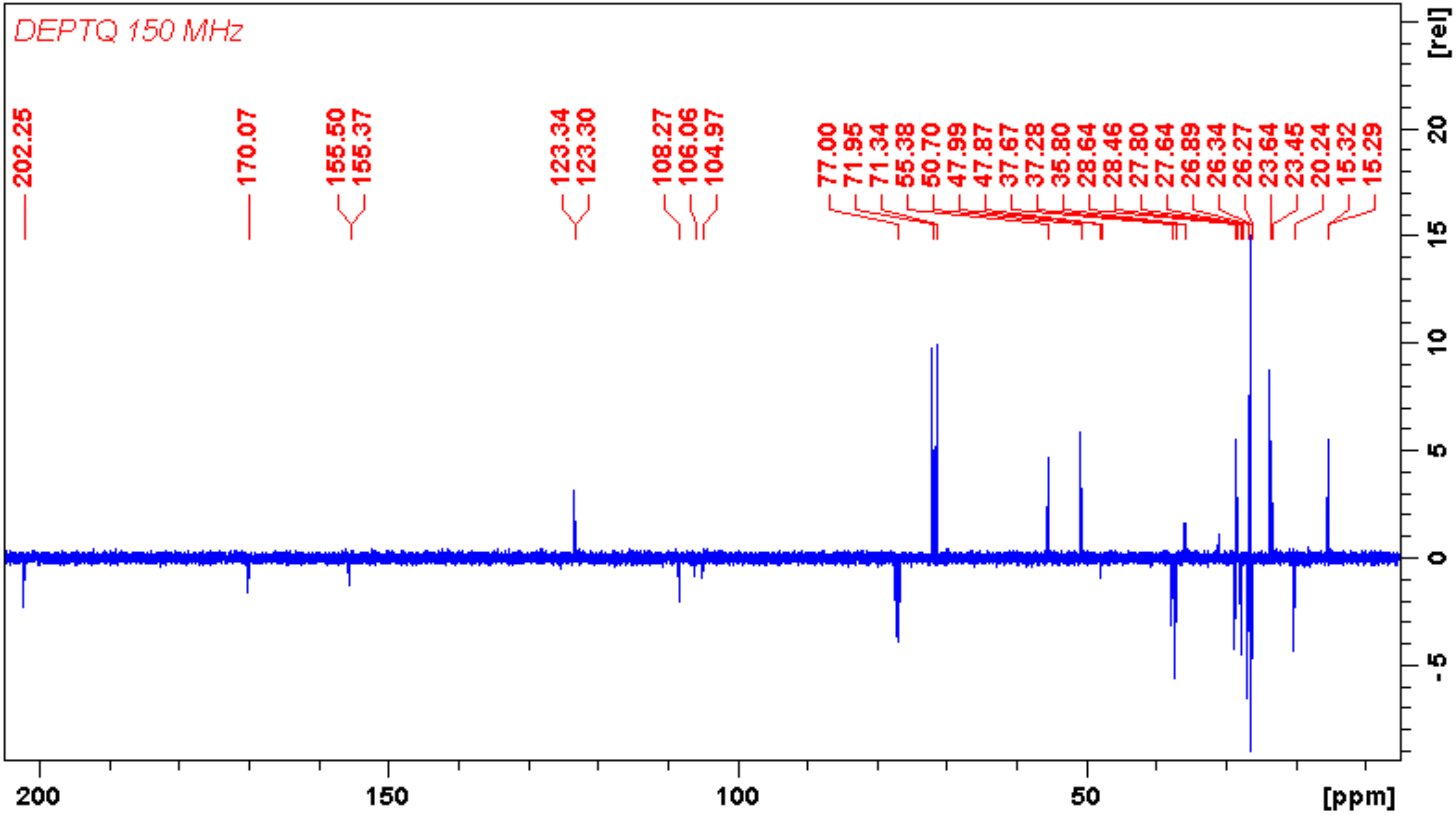

Figure S42. Compound **14**, edHSQC.

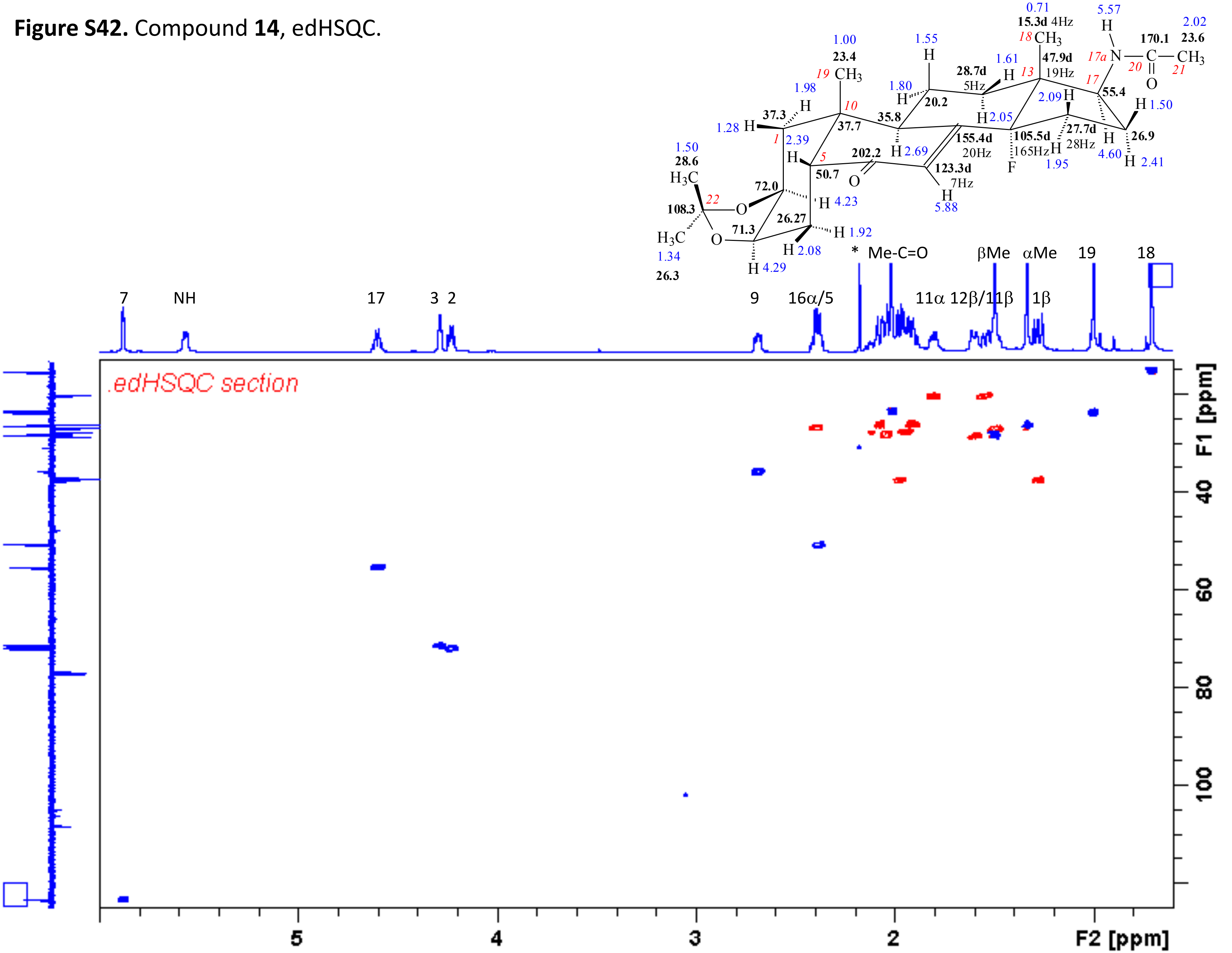

Figure S43. Compound **14**, edHSQC section with inserted selTOCSY on H-17.

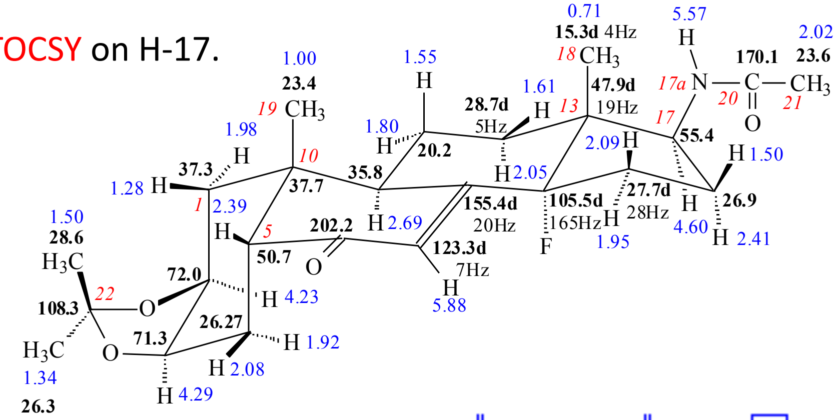

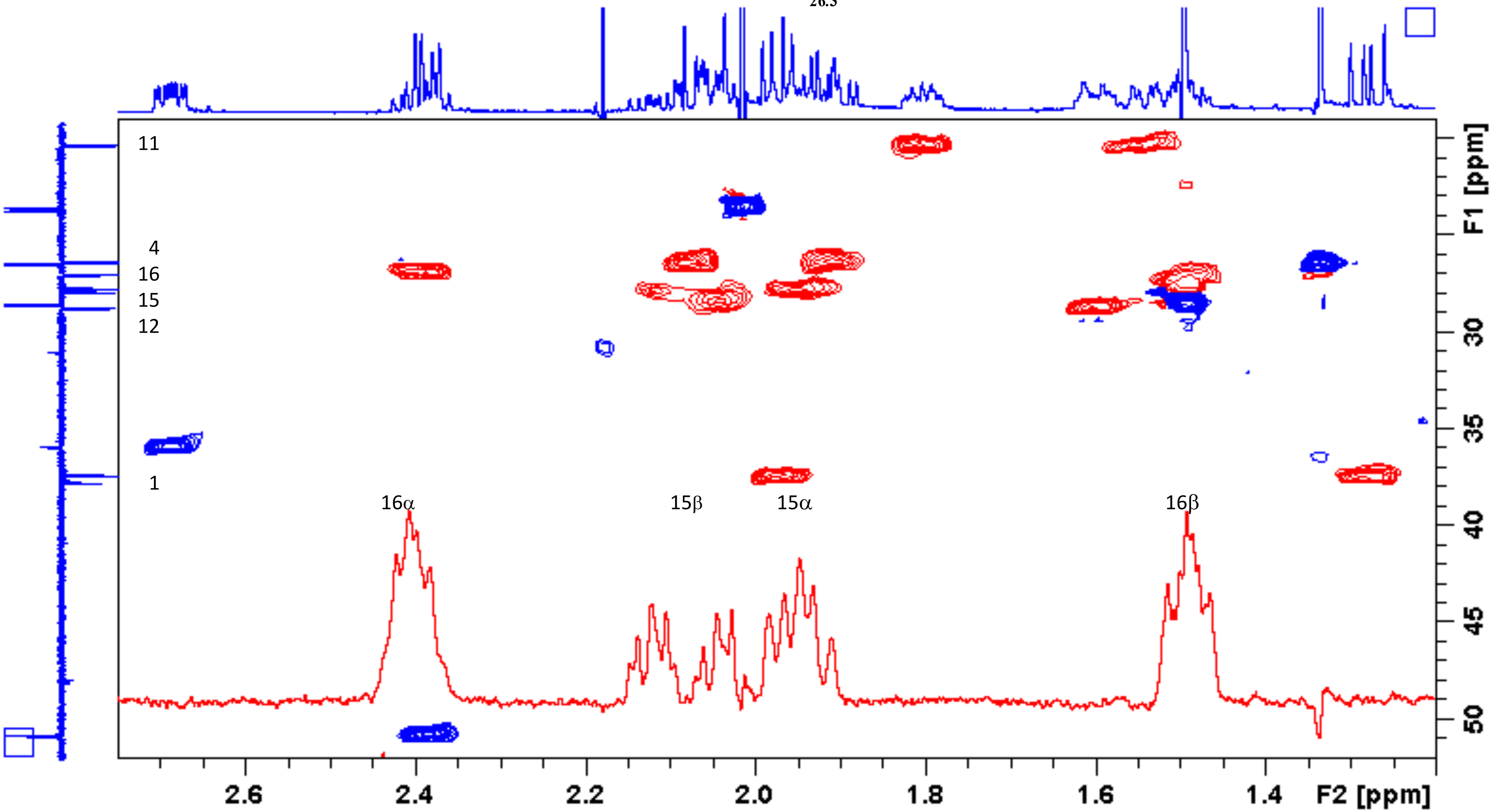

Figure S44. Compound **14**, HMBC.

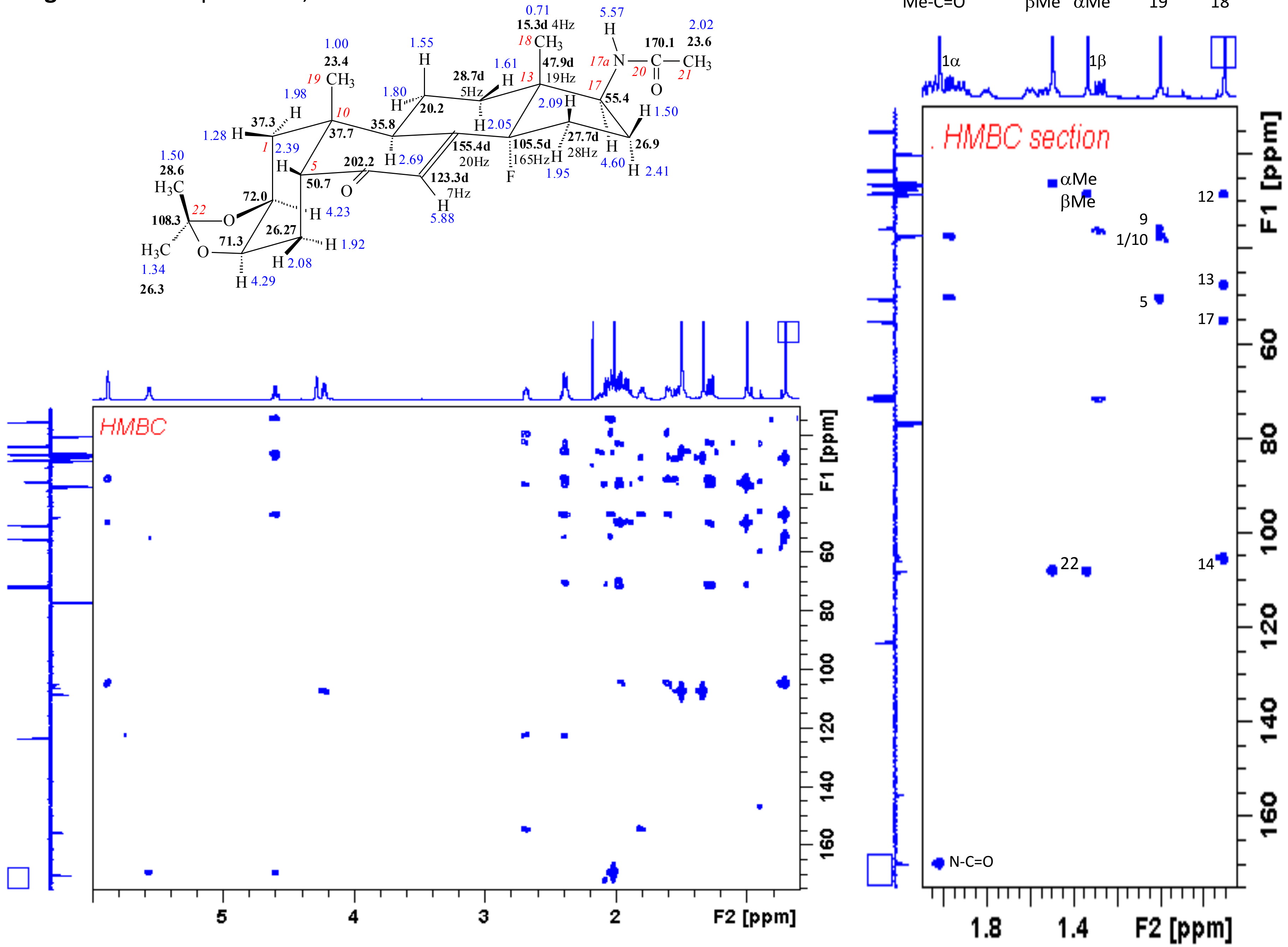

Figure S45. Compound **17**, ^{1}H DMSO-d_6 600 MHz.

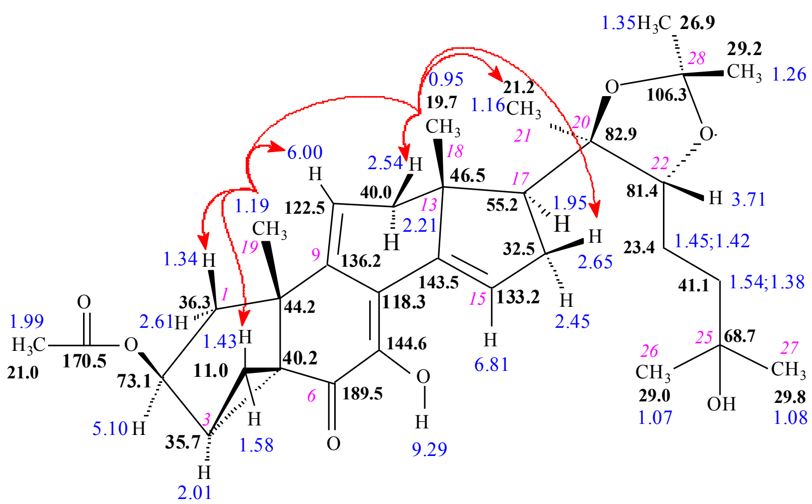

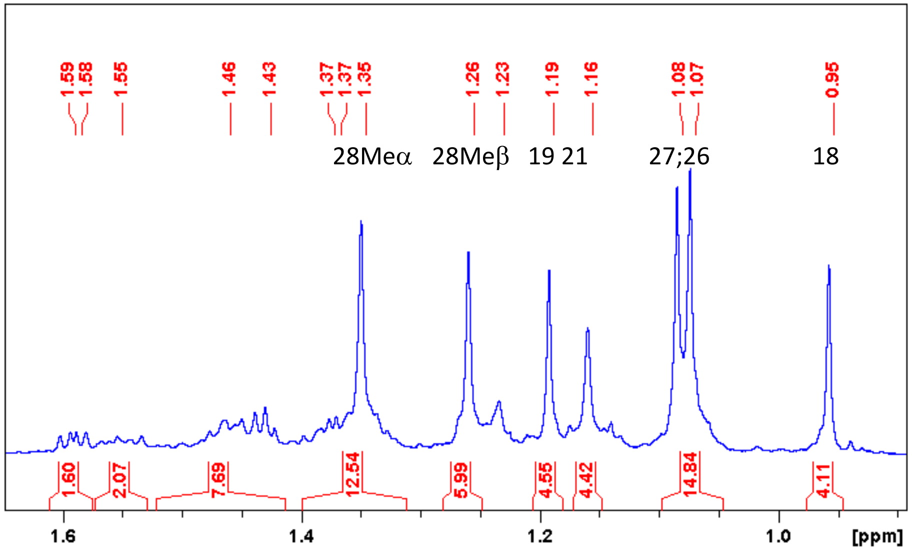

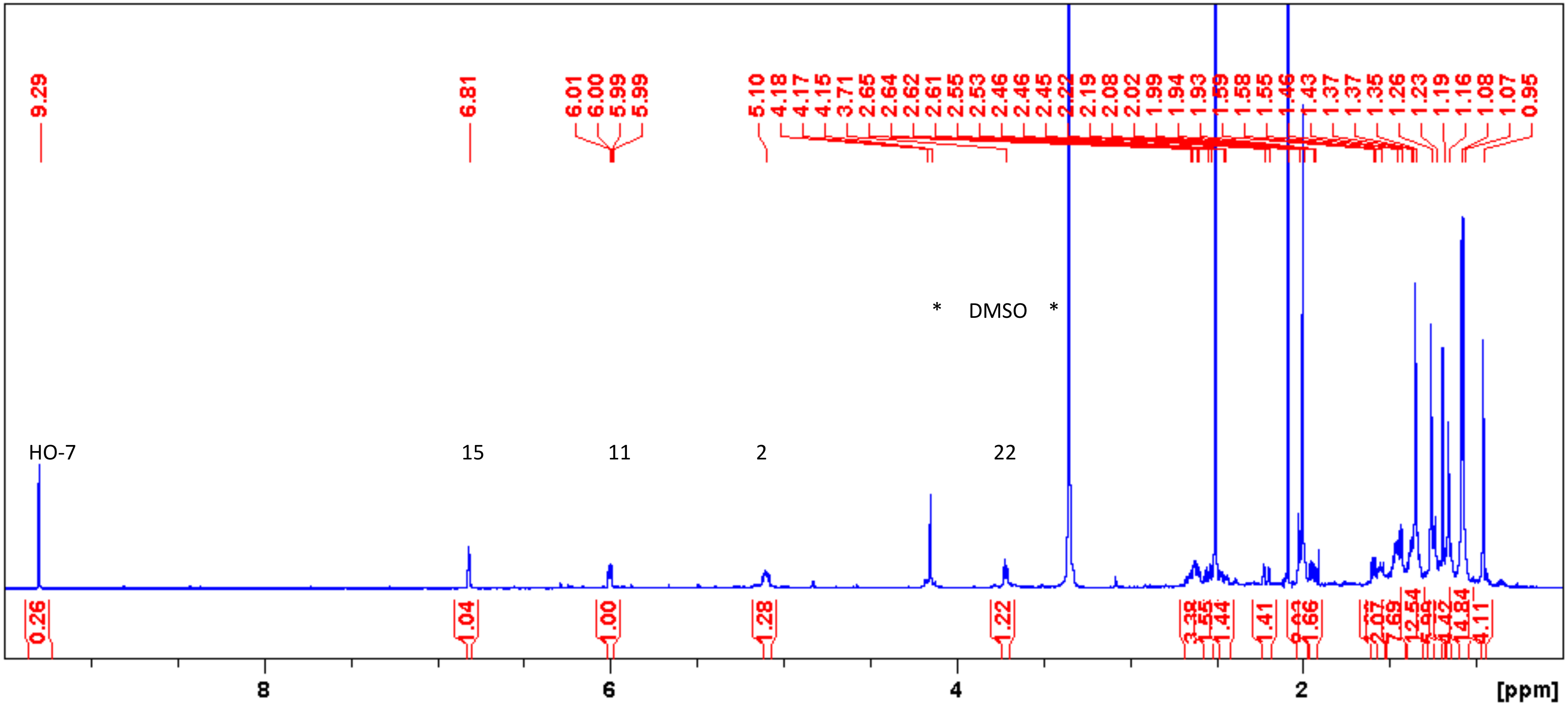

Figure S46. Compound **17**, steric proximities detected by selNOE on CH_3-19 and CH_3-18 signals and selTOCSY on H-2.

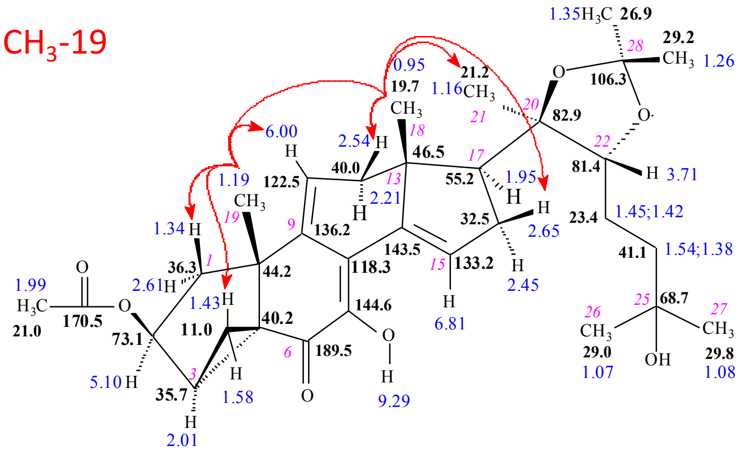

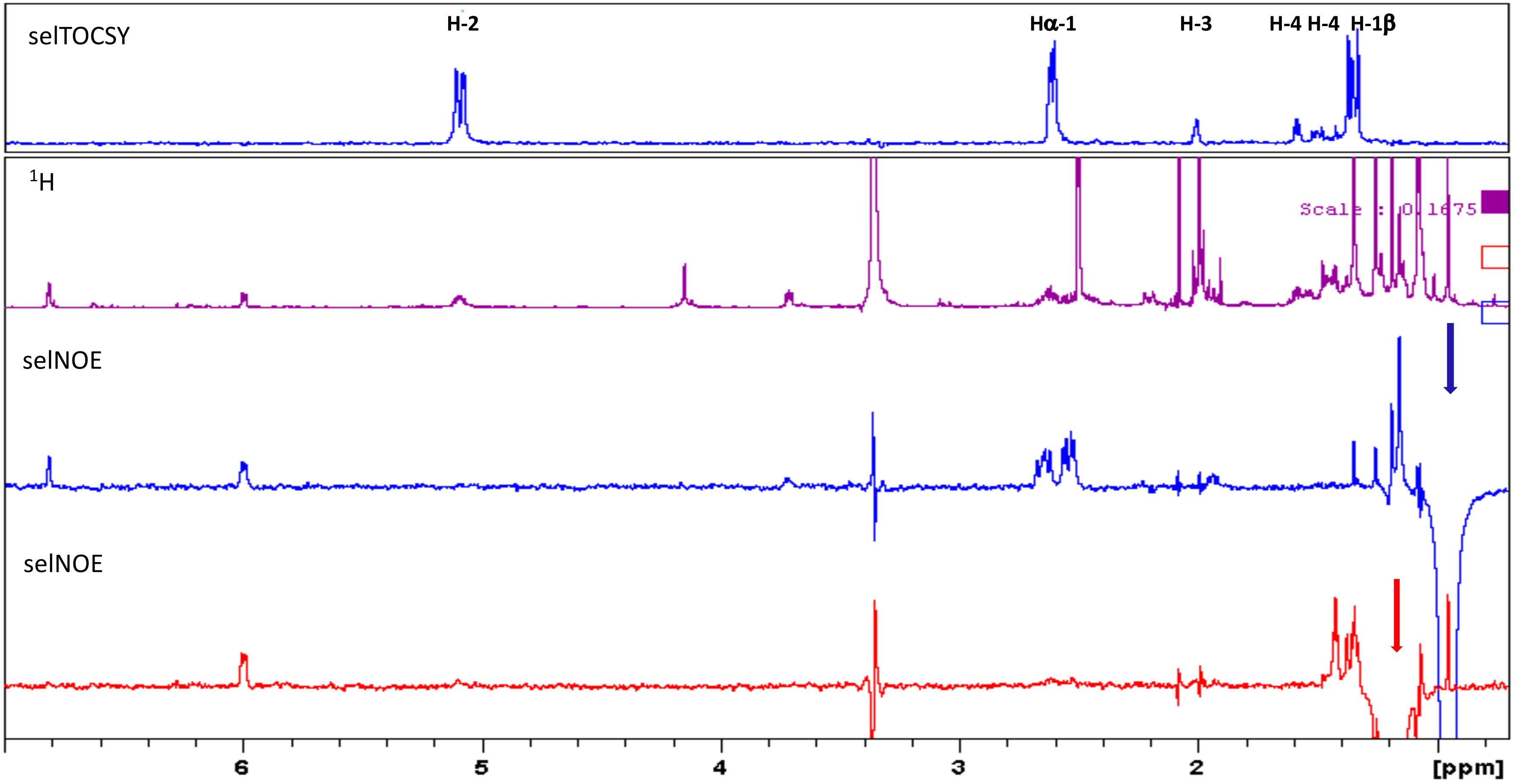

Figure S47. Compound **17**, DEPTQ 150 MHz.

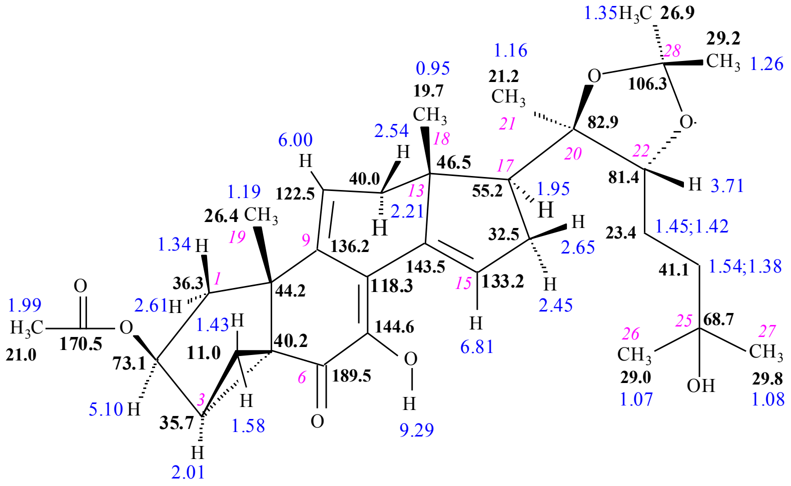

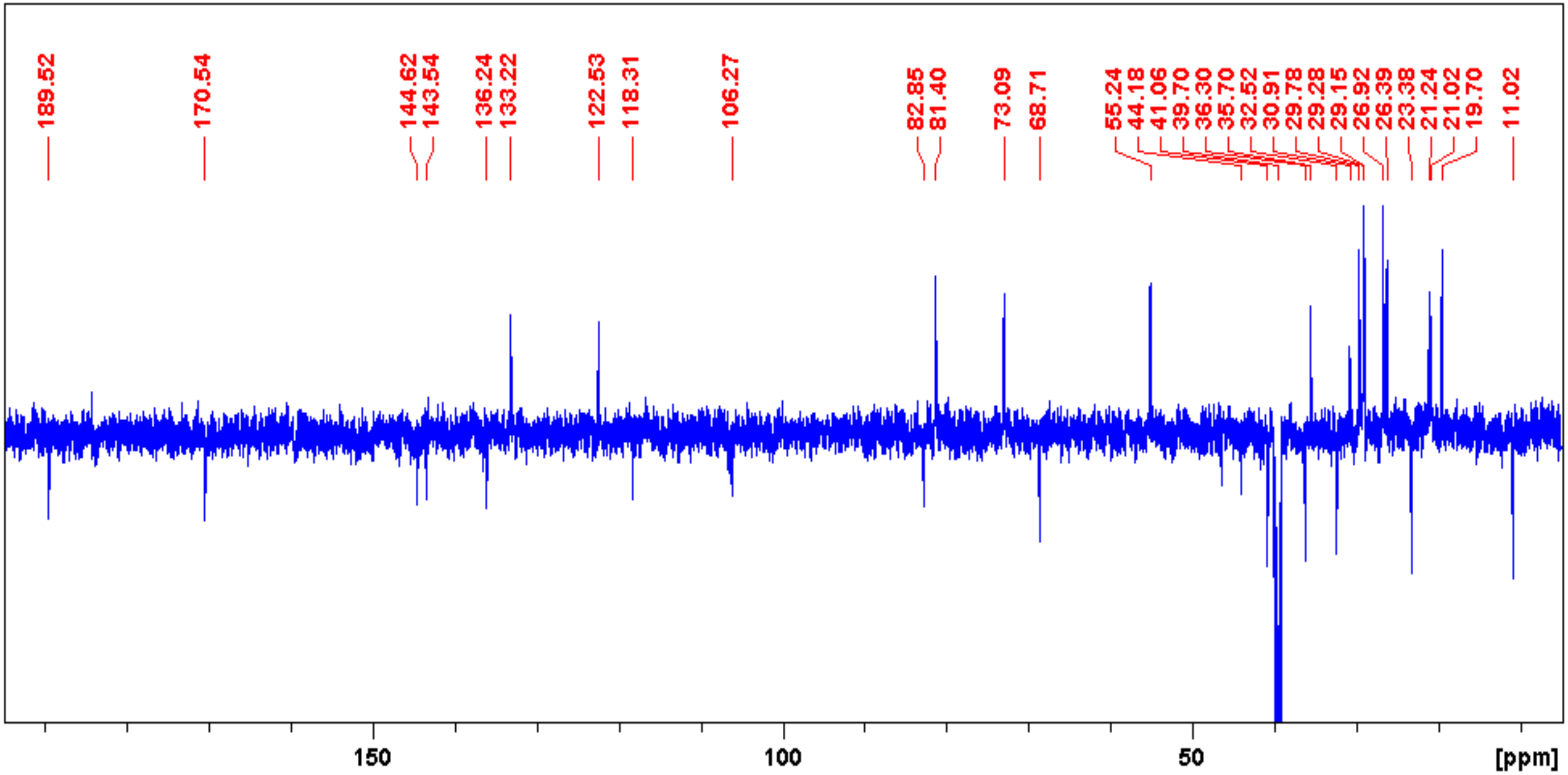

Figure S48. Compound **17**, HSQC.

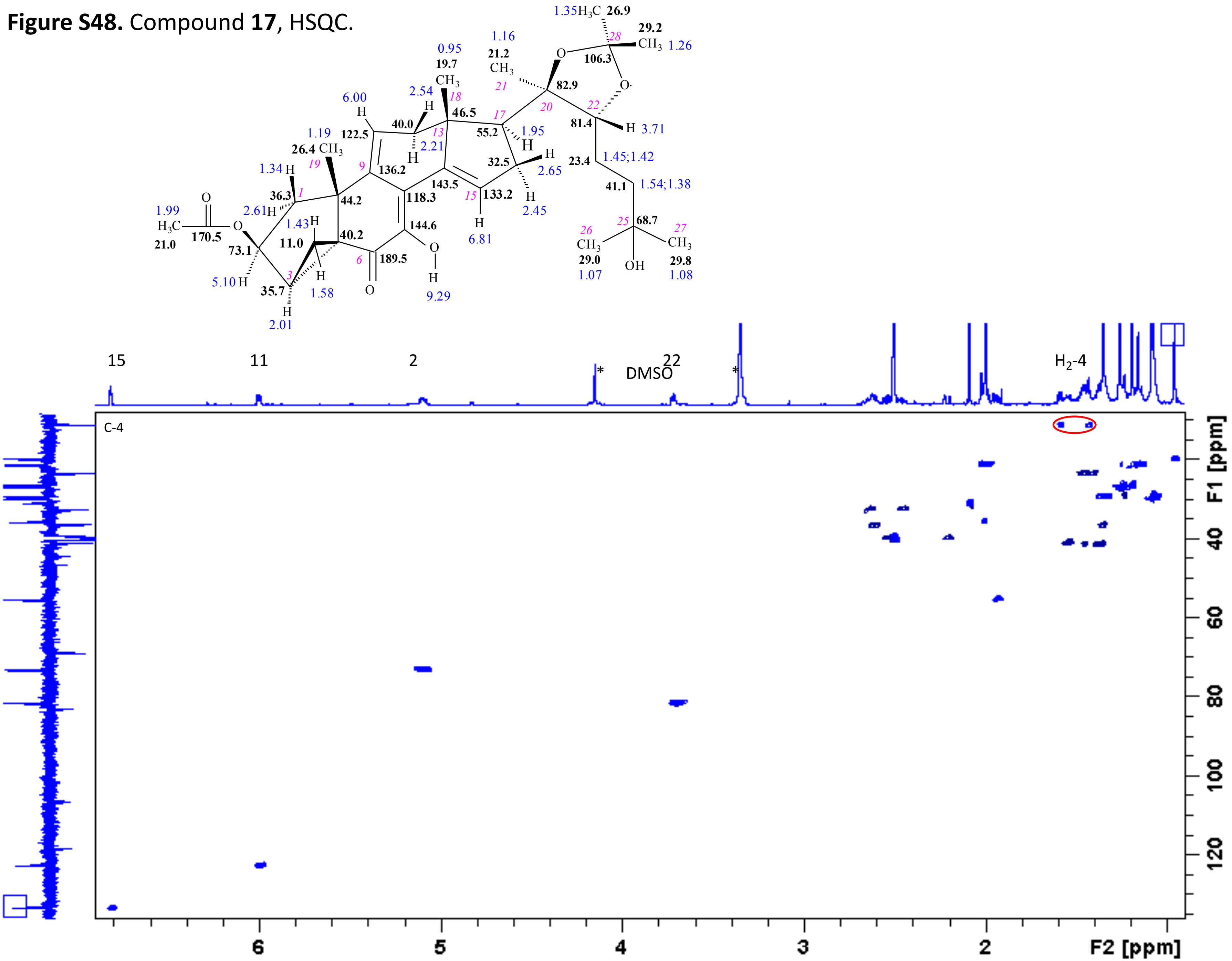

Figure S49. Compound **17**, HMBC and HMBC CH_3 section. Black arrows show CH_3/C responses.

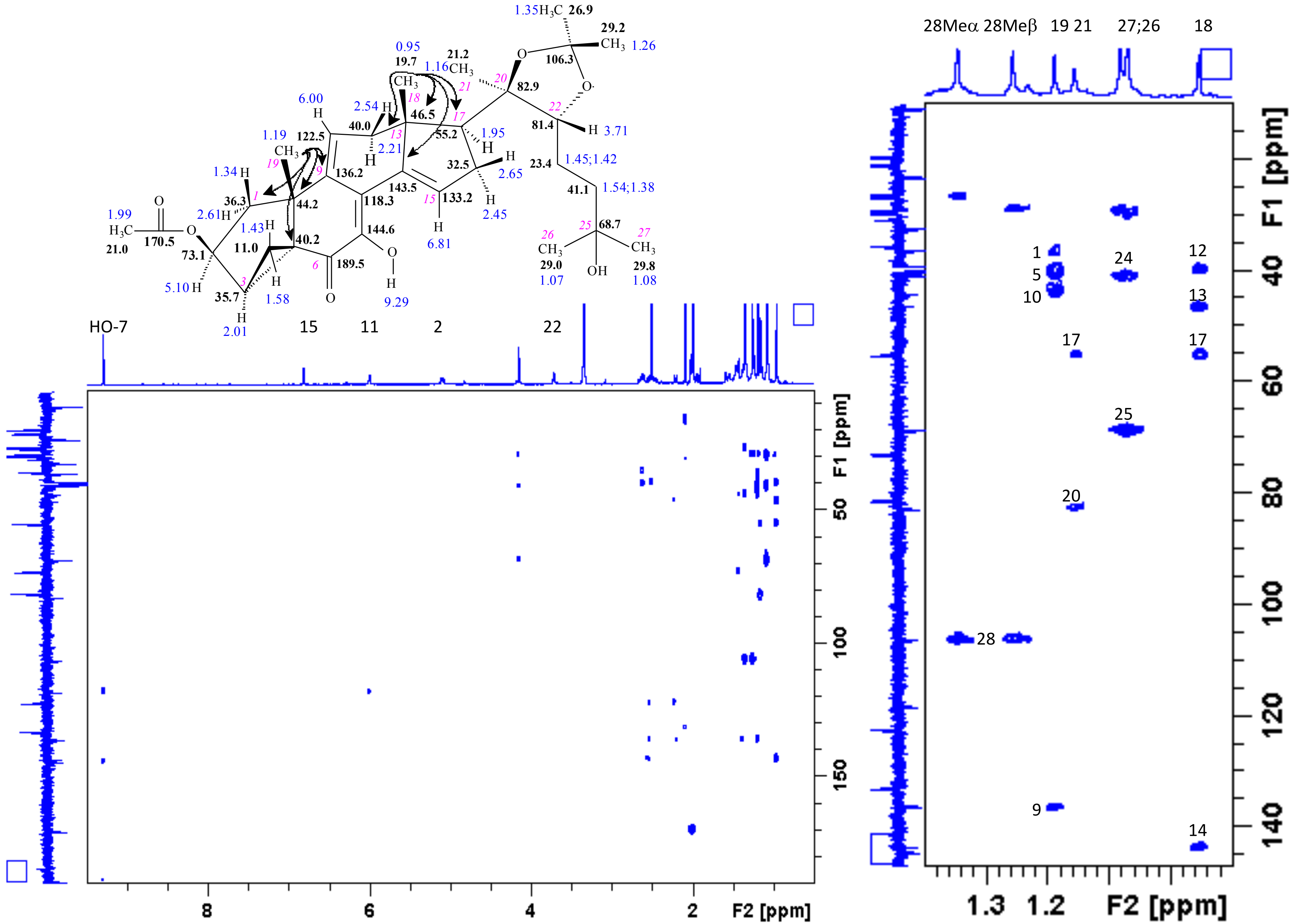

Figure S50. Compound **19**, ^{1}H $CDCl_3$ 500 MHz.

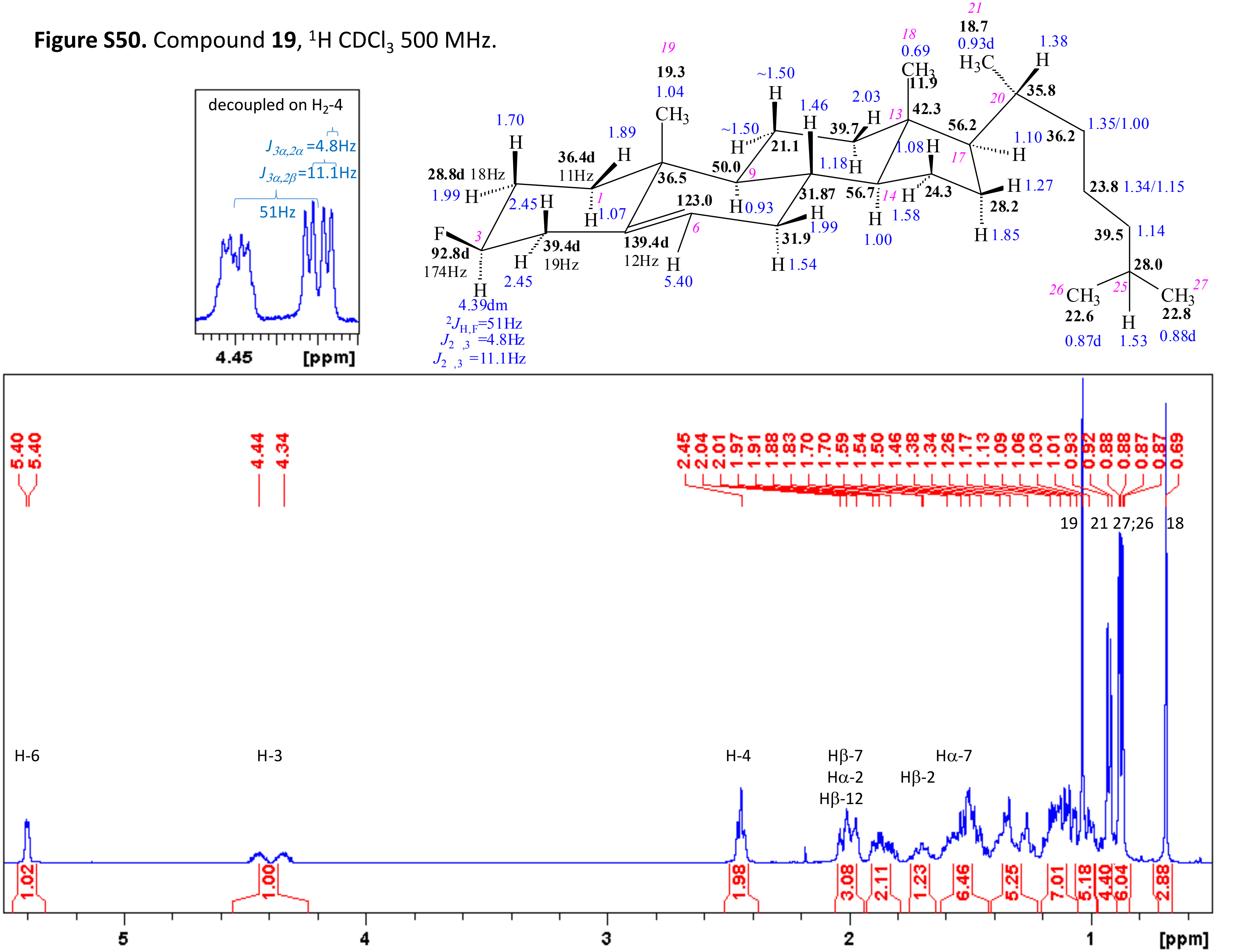

Figure S51. Compound **19**, $^{1}H,^{1}H$-C0SY and selROE on Hα-3.

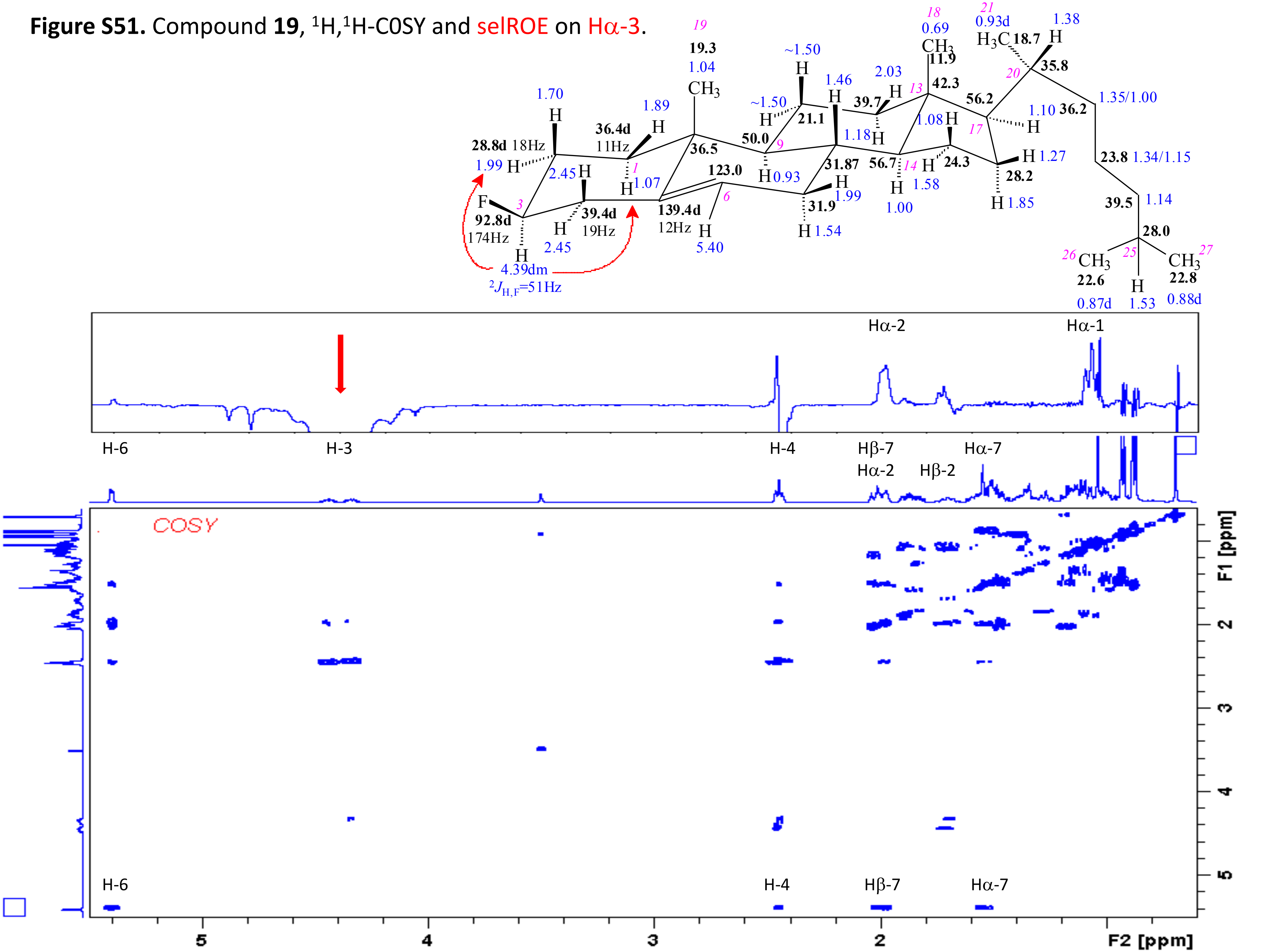

Figure S52. Compound **19**, APT 125 MHz.

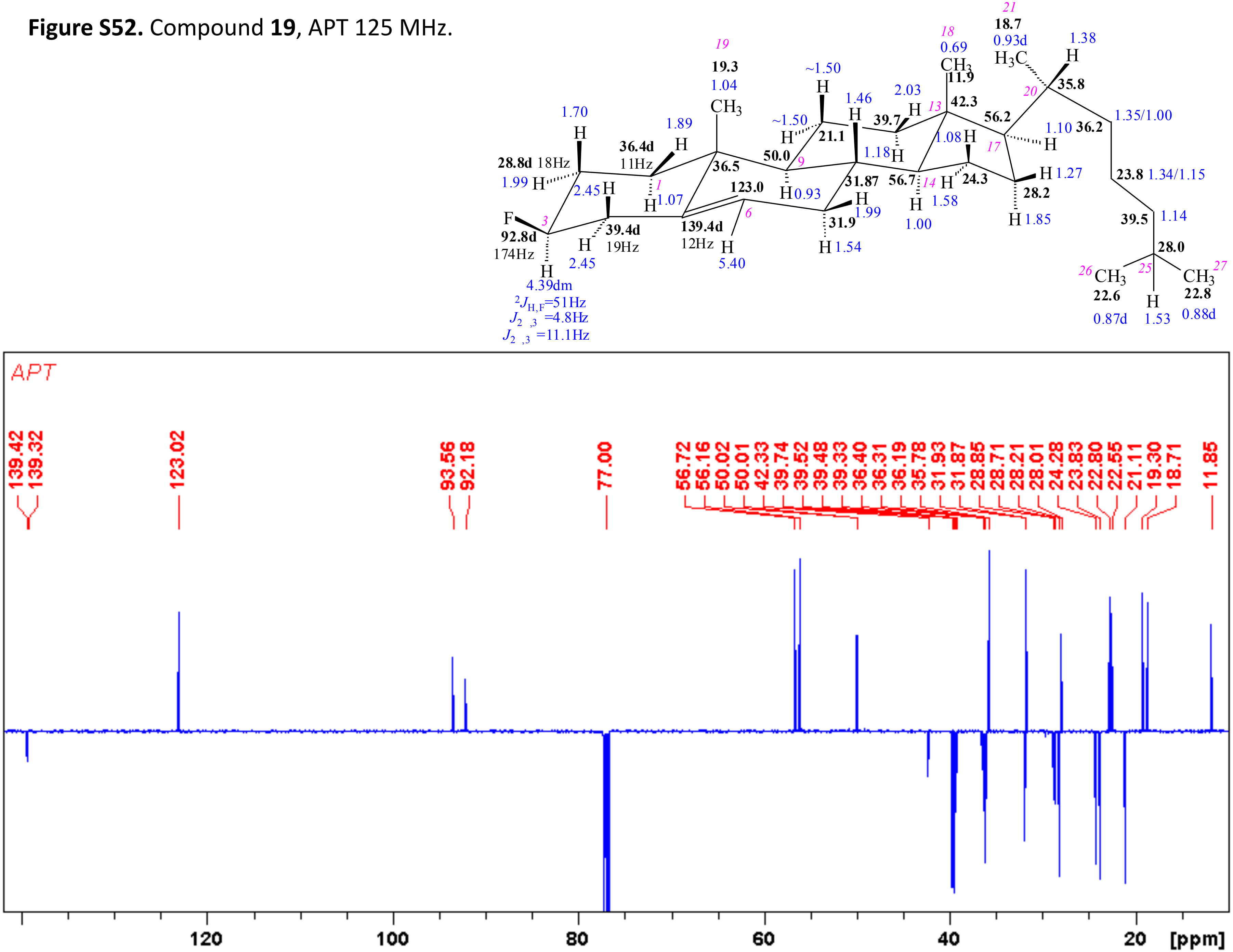

Figure S53. Compound **19**, HSQC section with inserted band-selective HSQC measurements of 37–36 and 40– 39ppm.

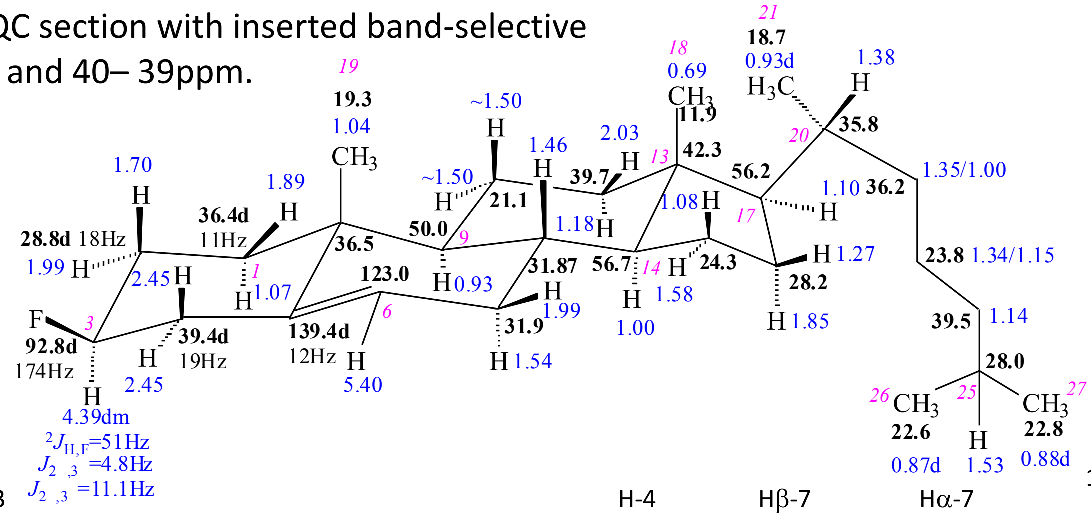

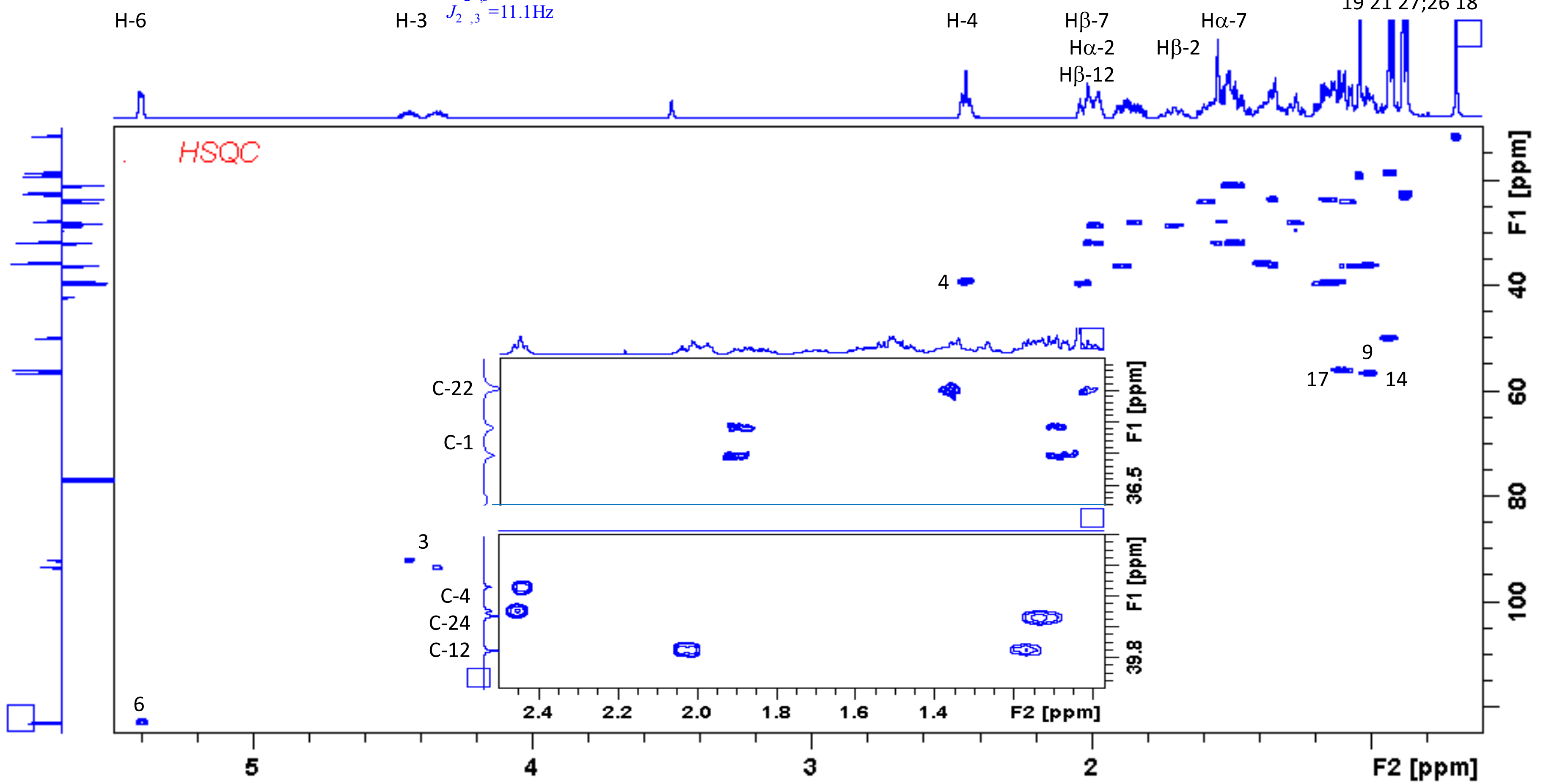

Figure S54. Compound **19**, edHSQC section with inserted selROE on H_3-18.

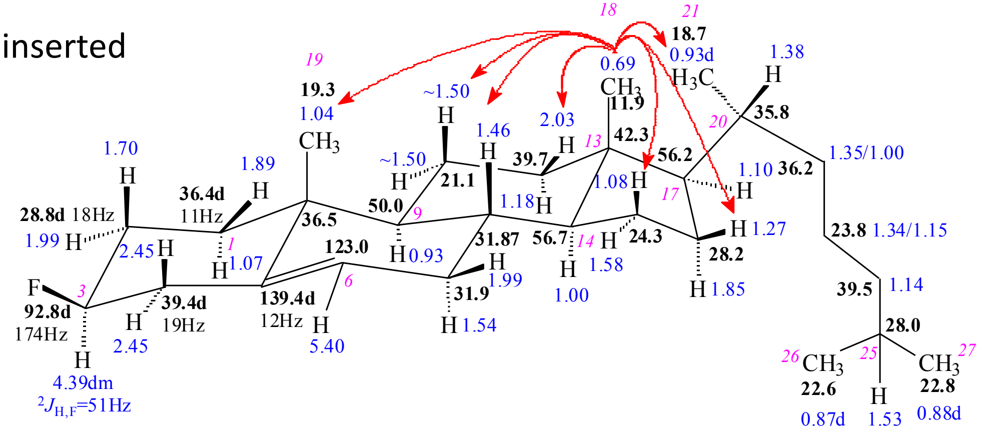

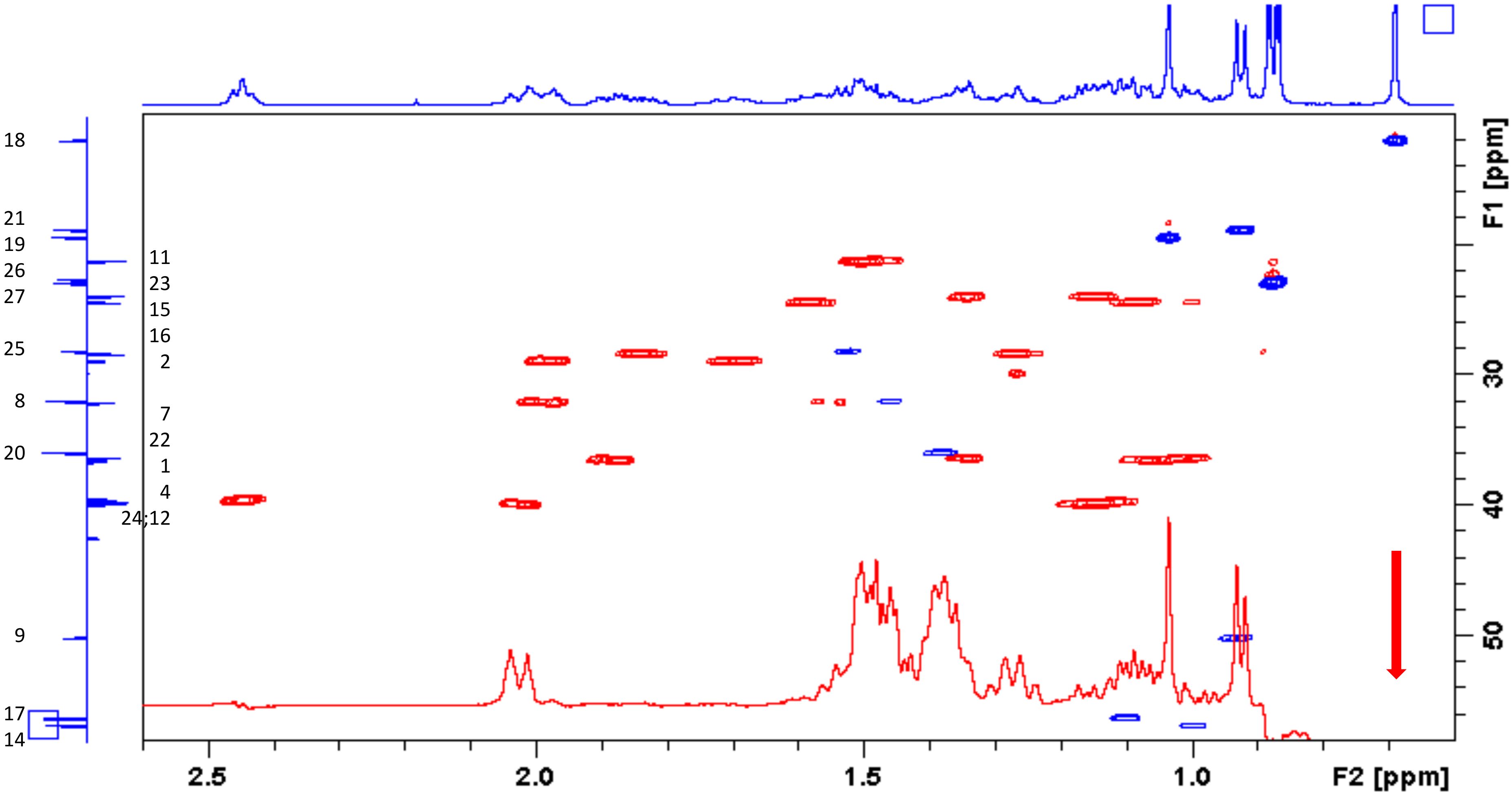

Figure S55. Compound **19**, HMBC and HMBC CH_3 section.

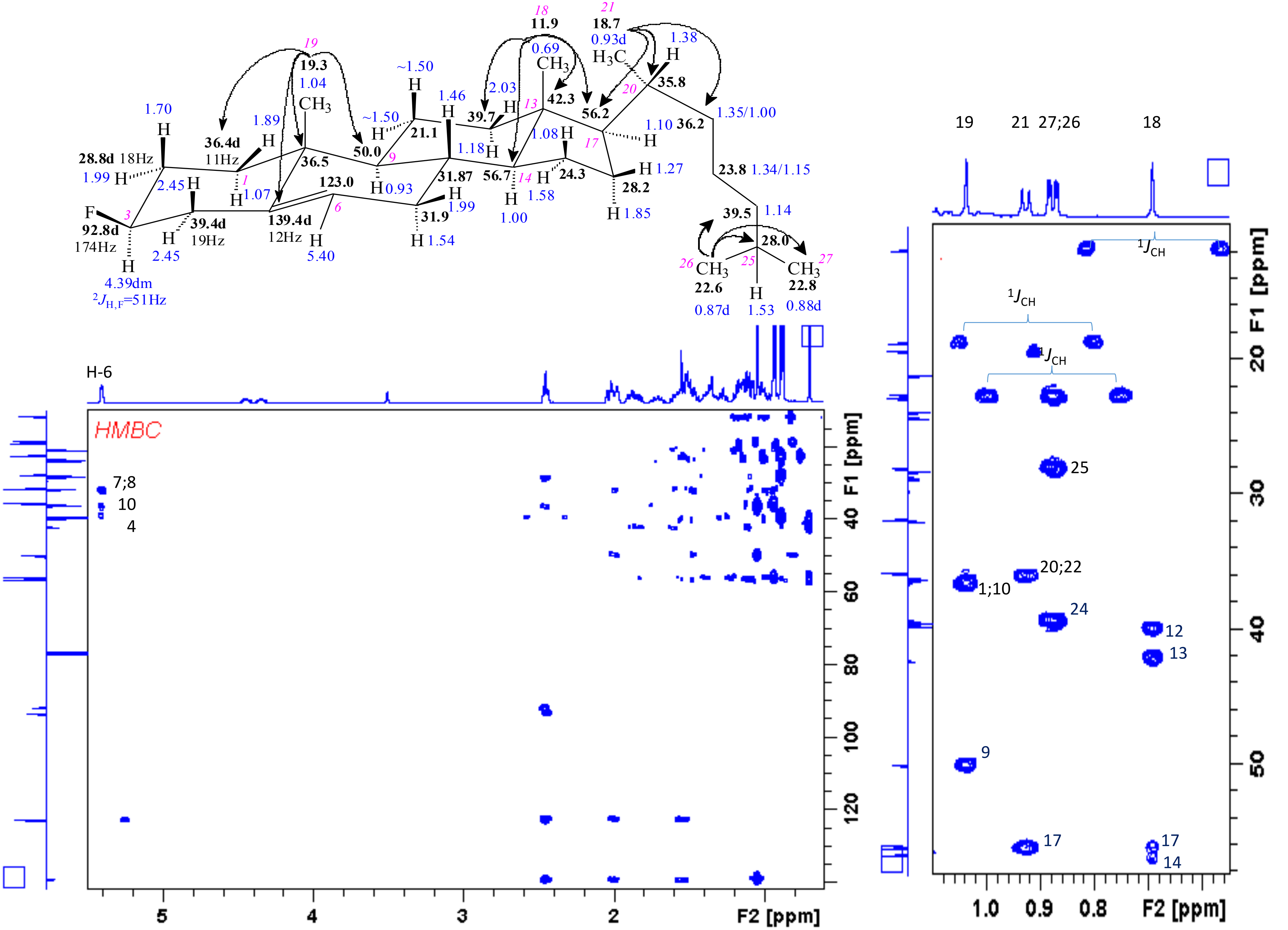

Figure S56. Compound **4**, HR-MS spectra.

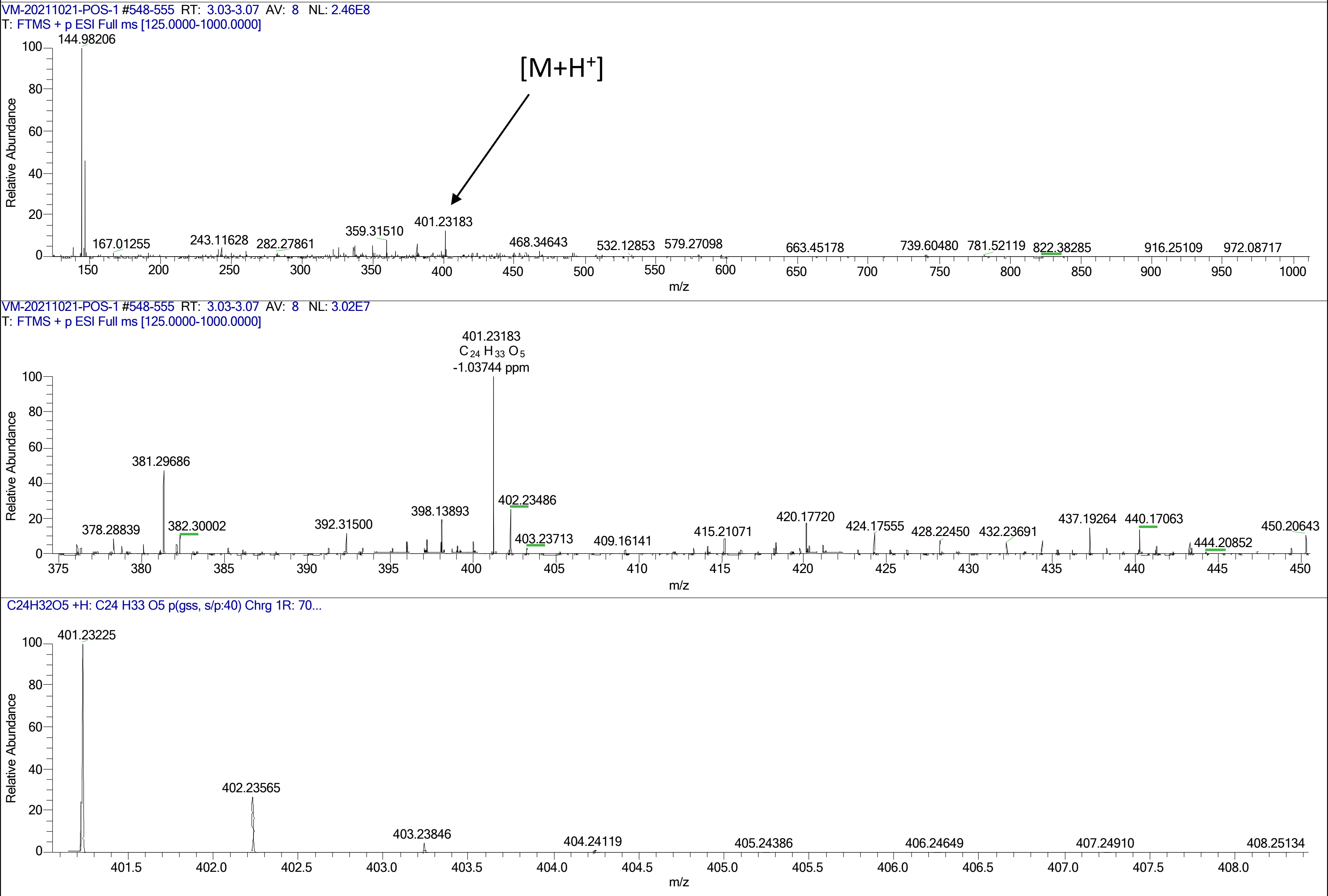

Figure S57. Compound **5**, HR-MS spectra.

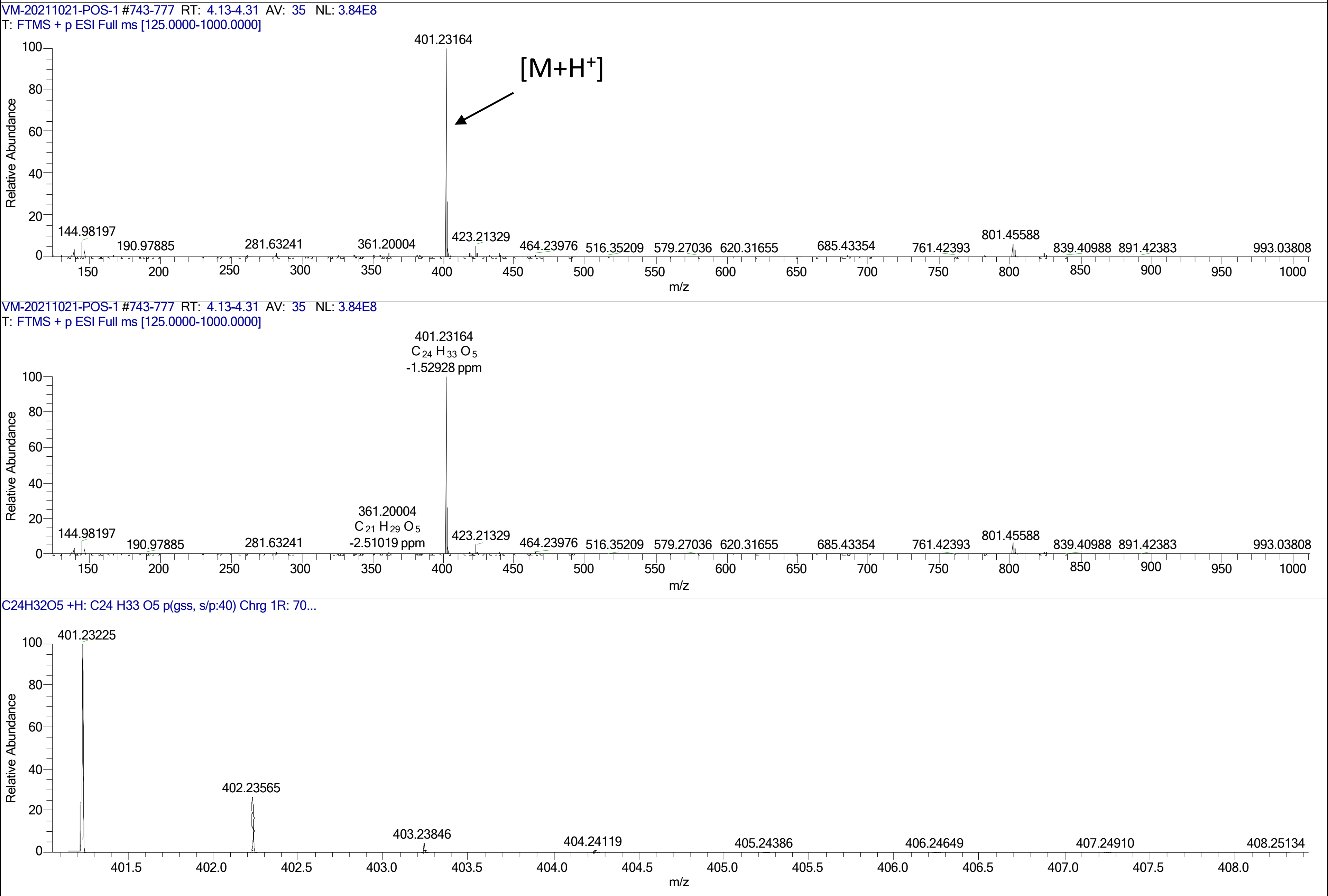

Figure S58. Compound **6**, HR-MS spectra.

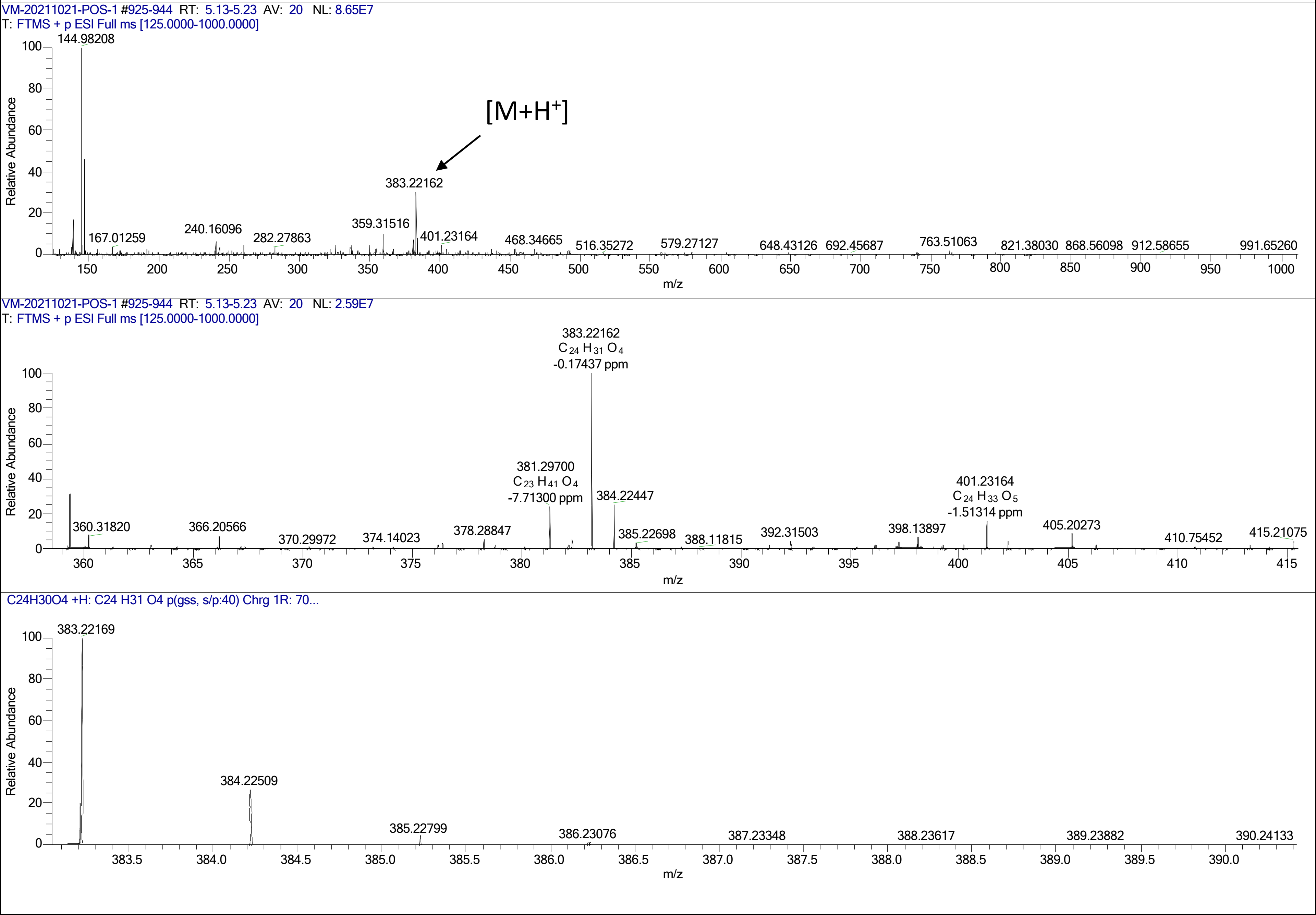

Figure S59. Compound **7**, HR-MS spectra.

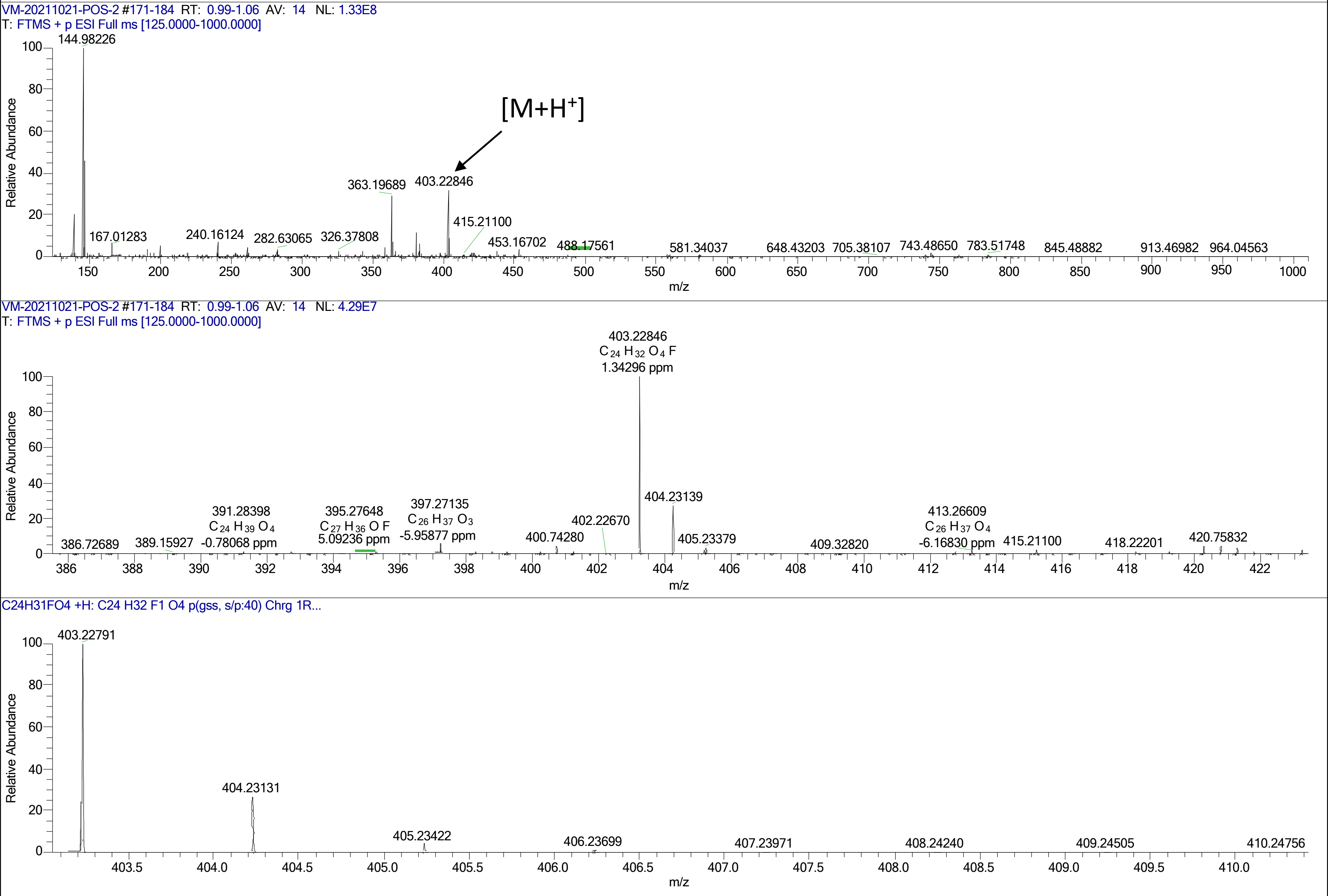

Figure S60. Compound **10**, HR-MS spectra.

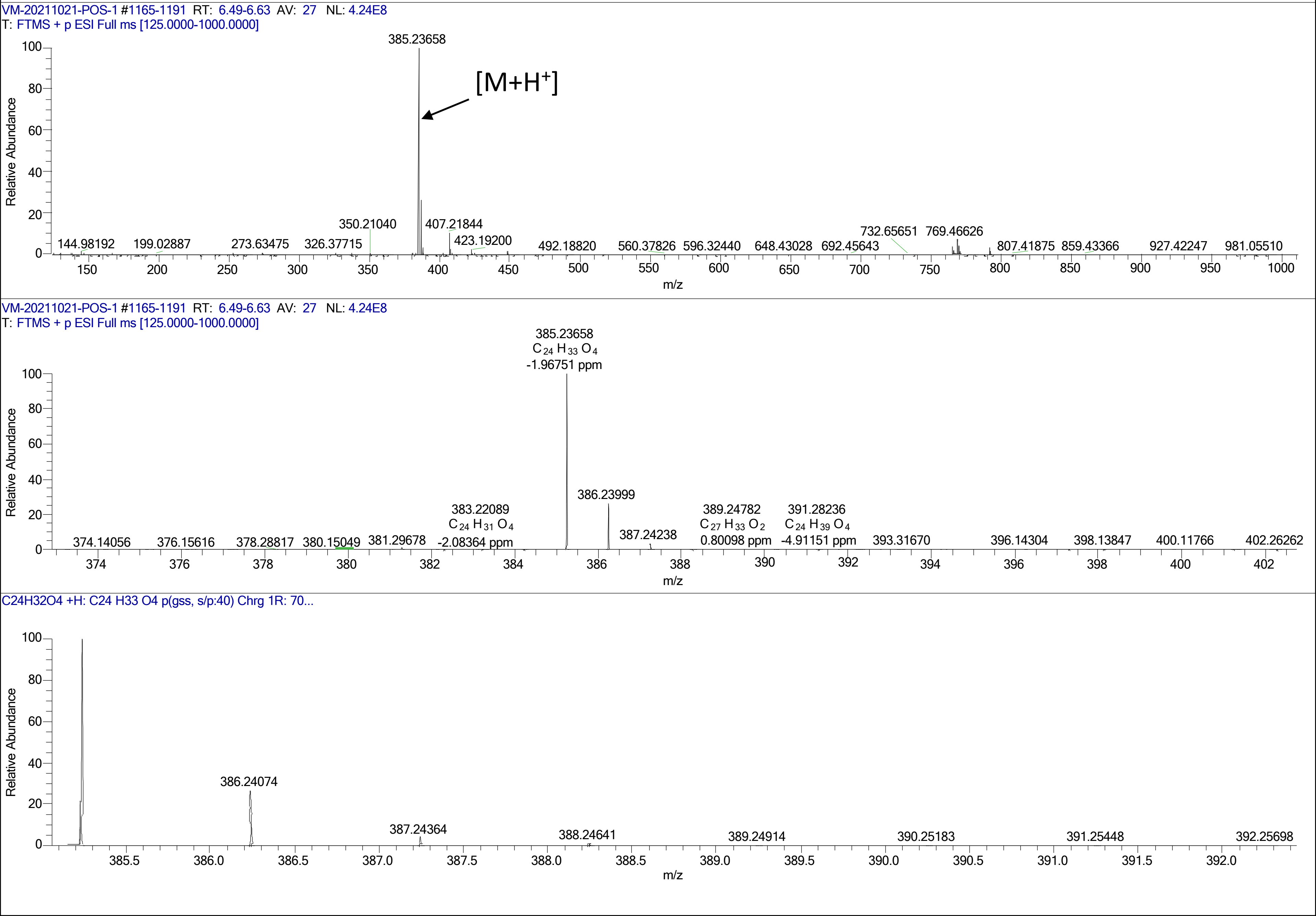

Figure S61. Compound **11**, HR-MS spectra.

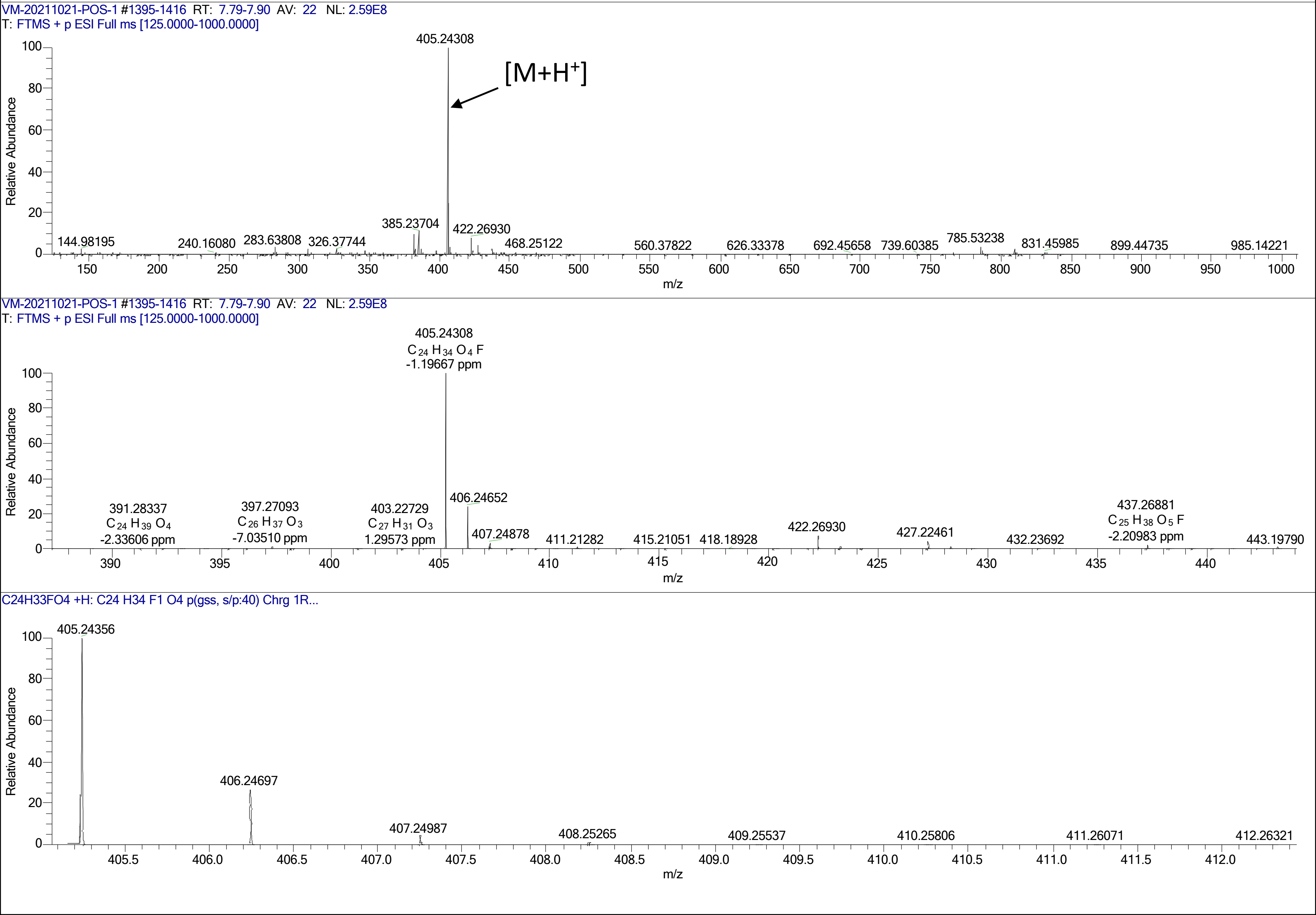

Figure S62. Compound **13**, HR-MS spectrum.

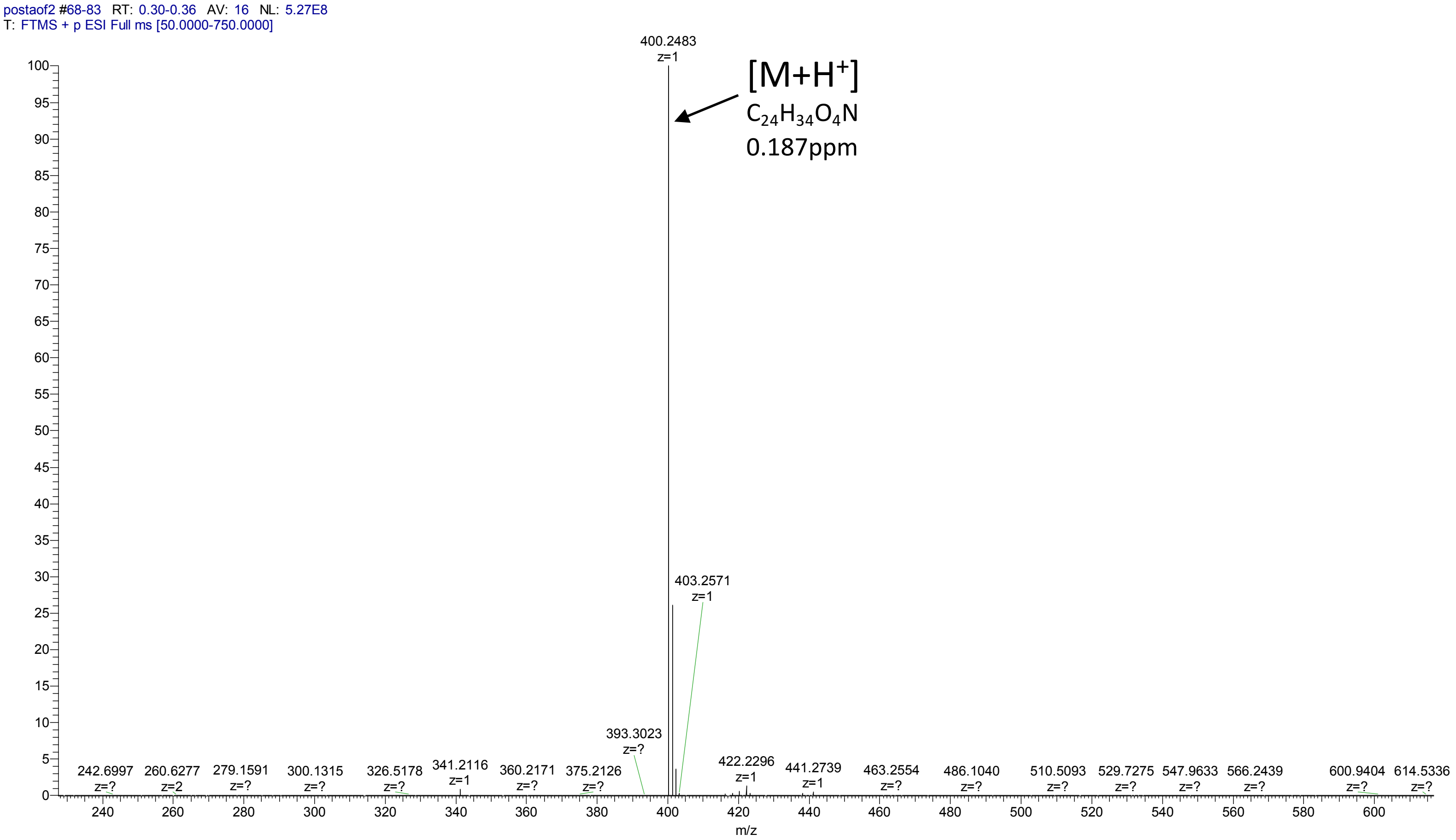

Figure S63. Compound **14**, HR-MS spectrum.

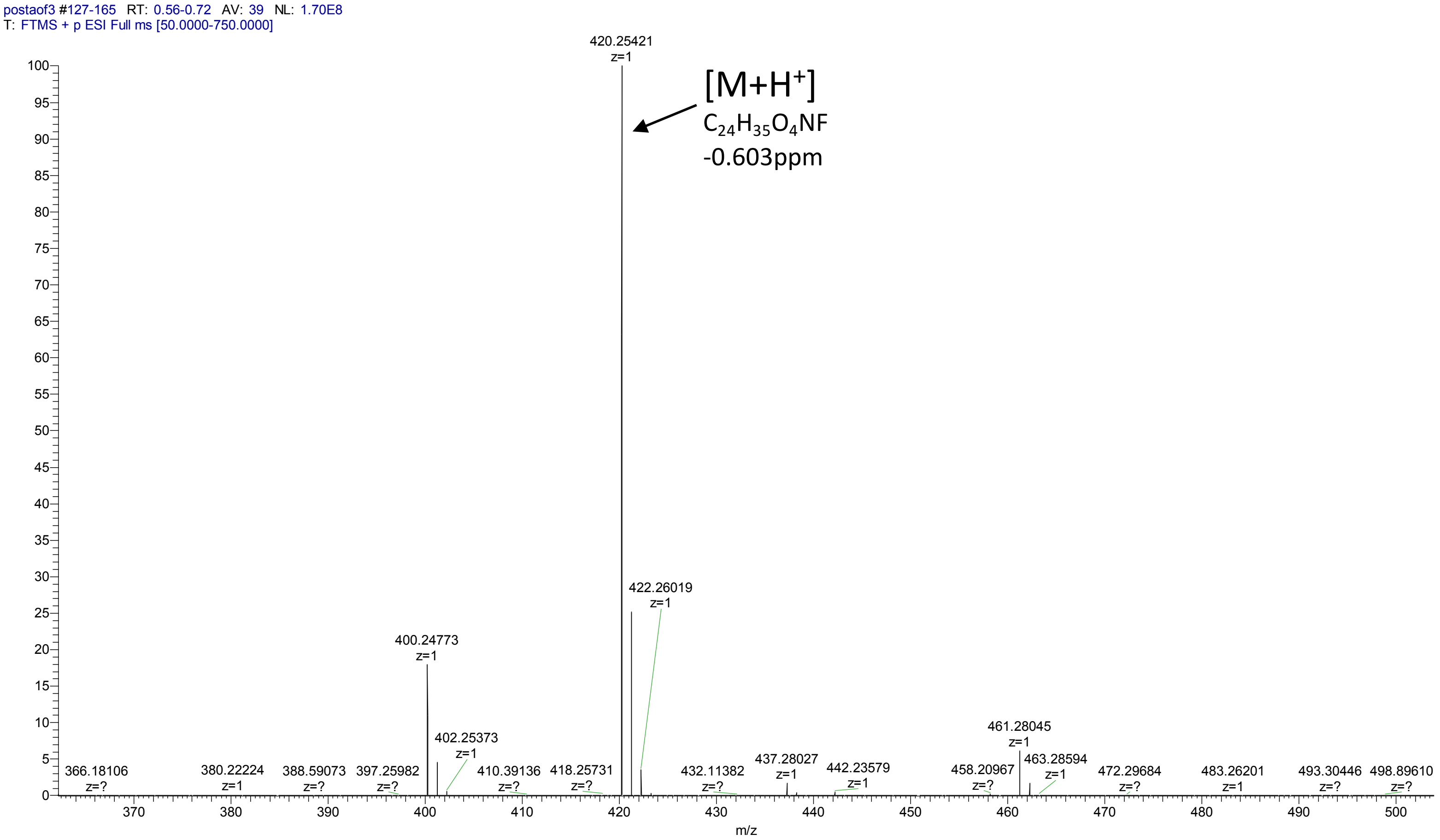

Figure S64. Compound **17**, HR-MS spectrum.

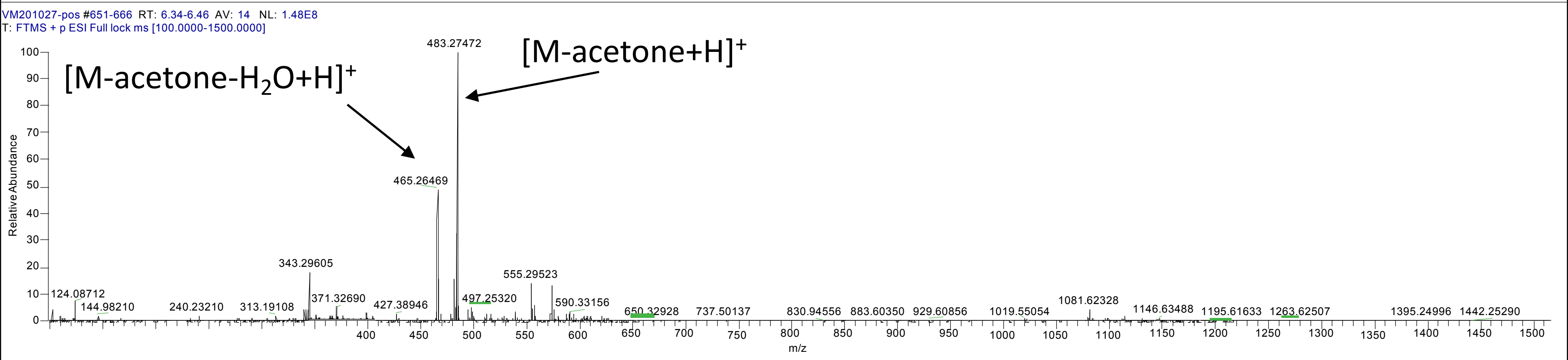

Figure S65. Compound **3**, HPLC chromatogram at its UV absorbance maximum (λ=242.6 nm). Purity: 95.1 %. Column: Kinetex®, 5 µm, XB-C18, 100 Å, 250 x 4.6 mm (Phenomenex Inc.); Elution: water:CH_3CN (A:B) 30→65% B.

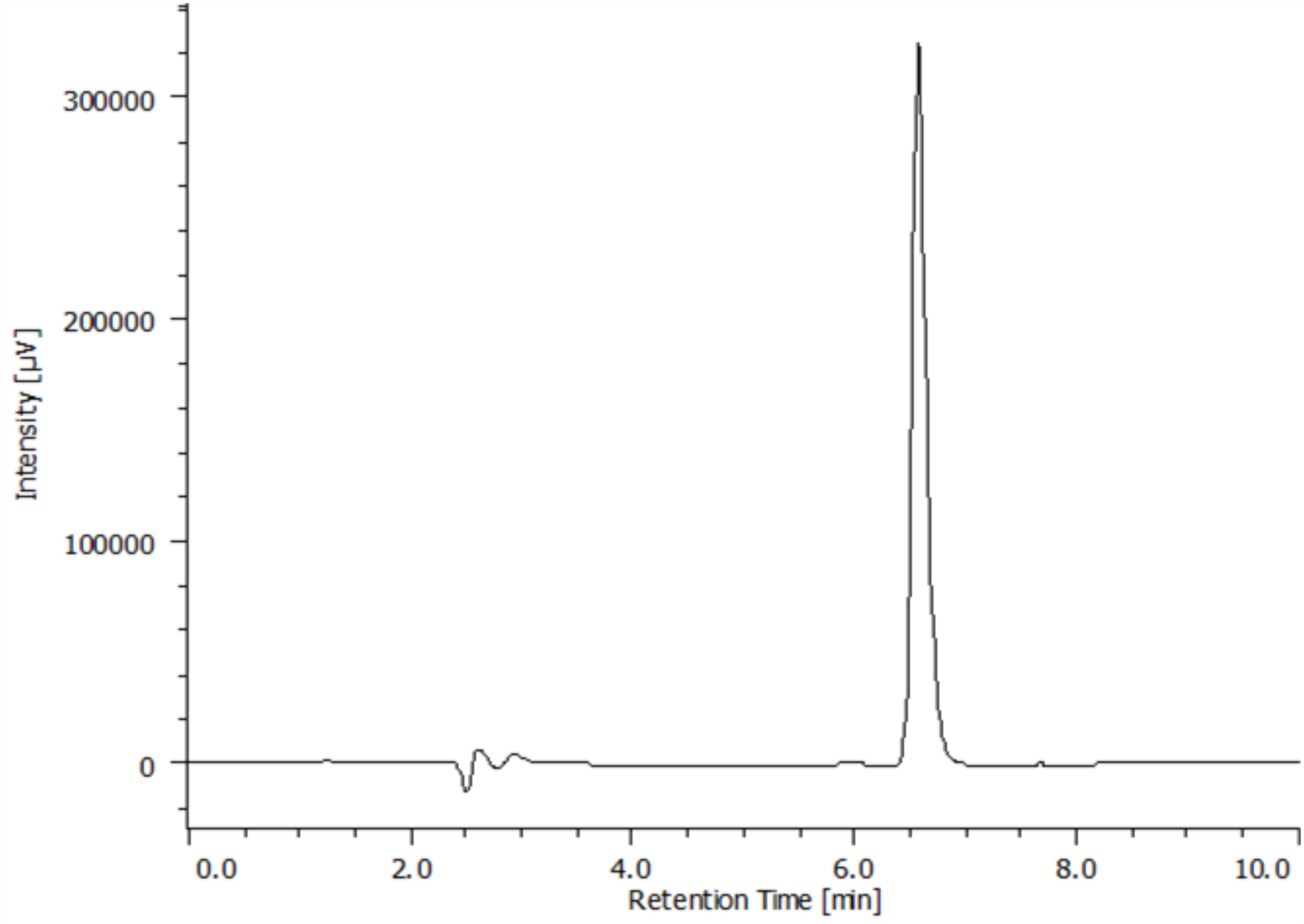

Figure S66. Compound **4**, HPLC chromatogram at its UV absorbance maximum (λ=300 nm). Purity: 97.5 %. Column: Luna®, 5 µm, Phenyl-Hexyl, 100 Å, 250 x 4.6 mm (Phenomenex Inc.); Elution: water:CH_3CN (A:B) 44% B.

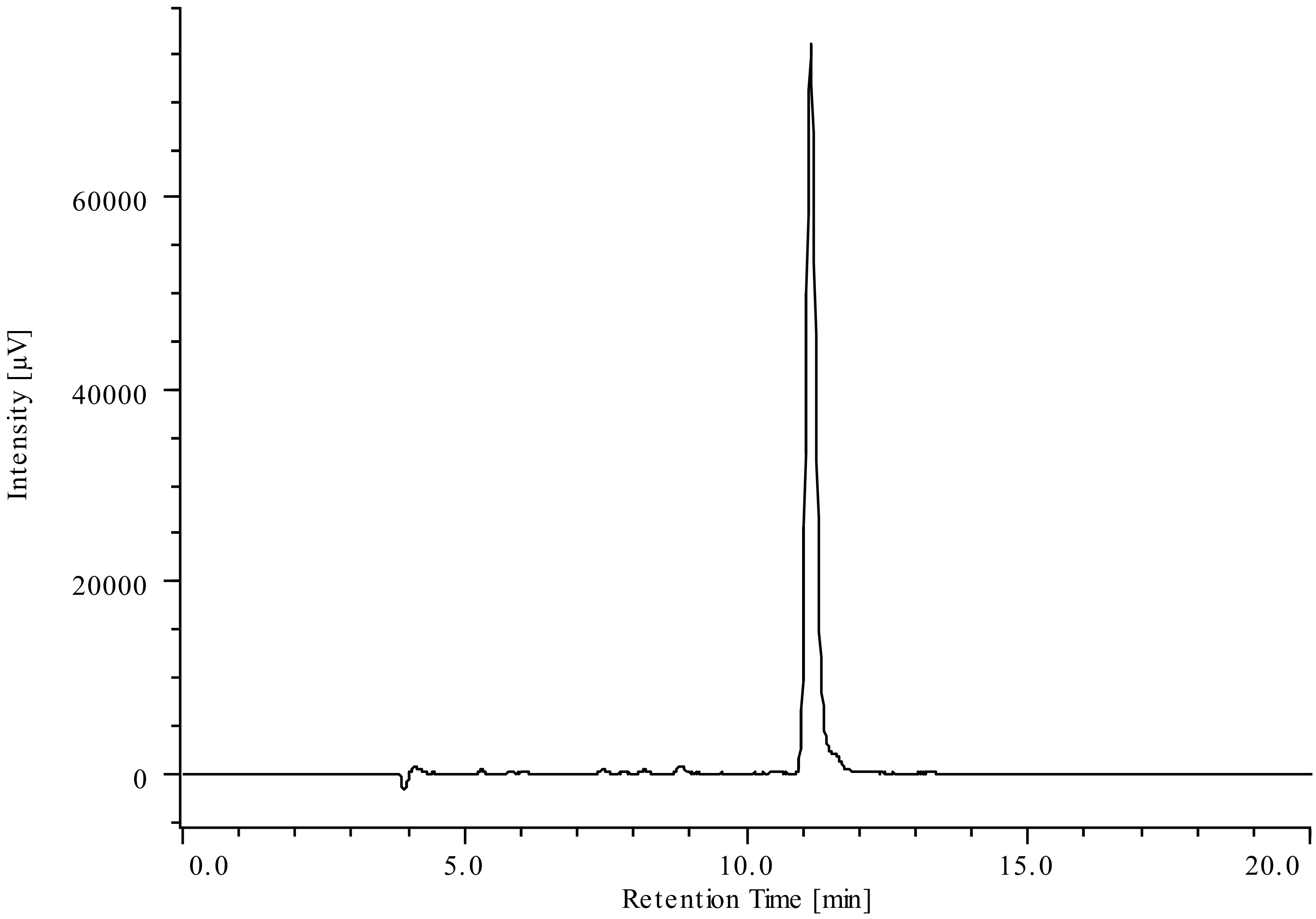

Figure S67. Compound **5**, HPLC chromatogram at its UV absorbance maximum (λ=300 nm). Purity: 97.5 %. Column: Luna®, 5 µm, Phenyl-Hexyl, 100 Å, 250 x 4.6 mm (Phenomenex Inc.); Elution: water:CH_3CN (A:B) 44% B.

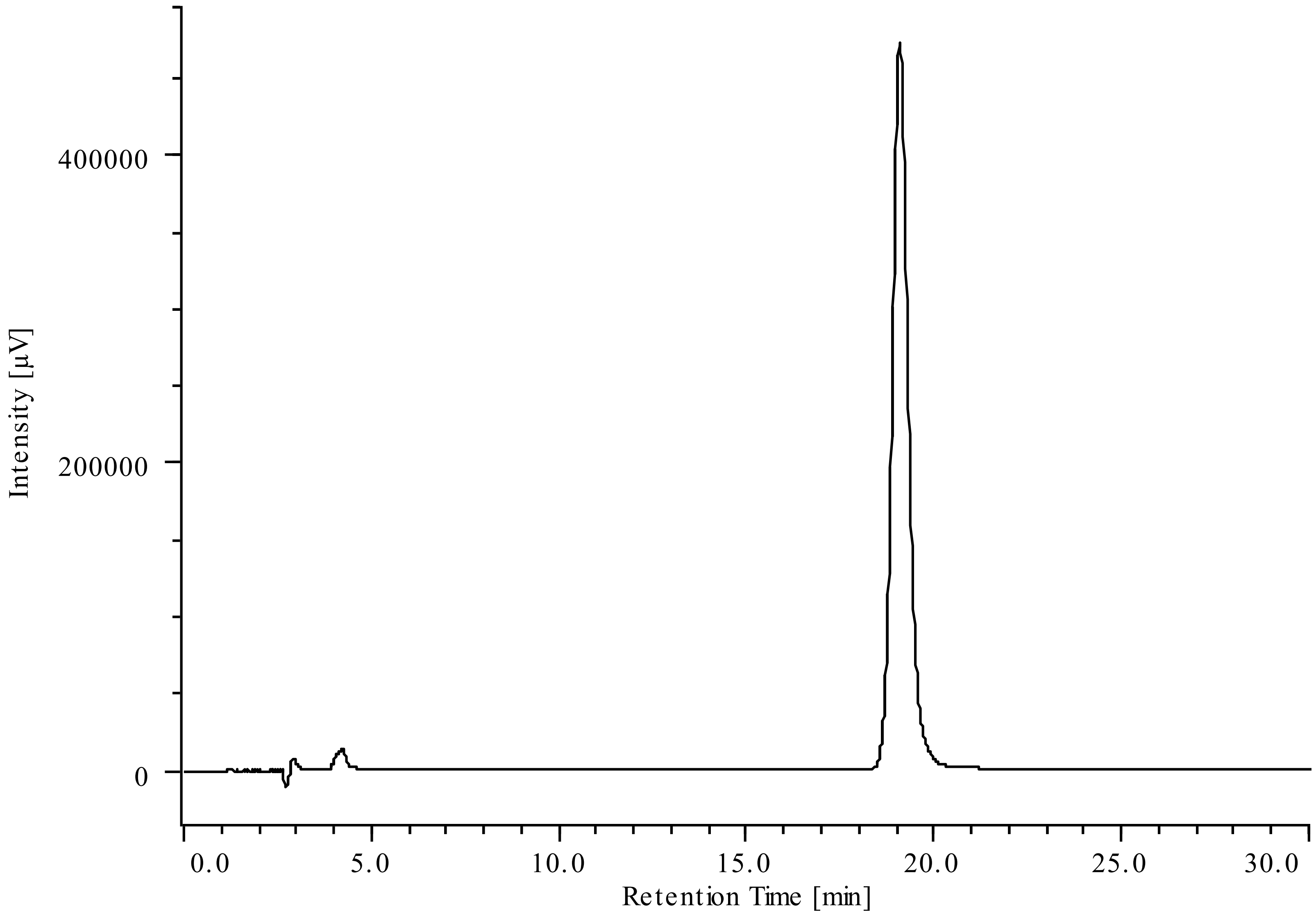

Figure S68. Compound **6**, HPLC chromatogram at its UV absorbance maximum (λ=327.5 nm). Purity: 95.2 %. Column: Kinetex®, 5 µm, Biphenyl, 100 Å, 250 x 4.6 mm (Phenomenex Inc.); Elution: water:CH_3CN (A:B) 40% B.

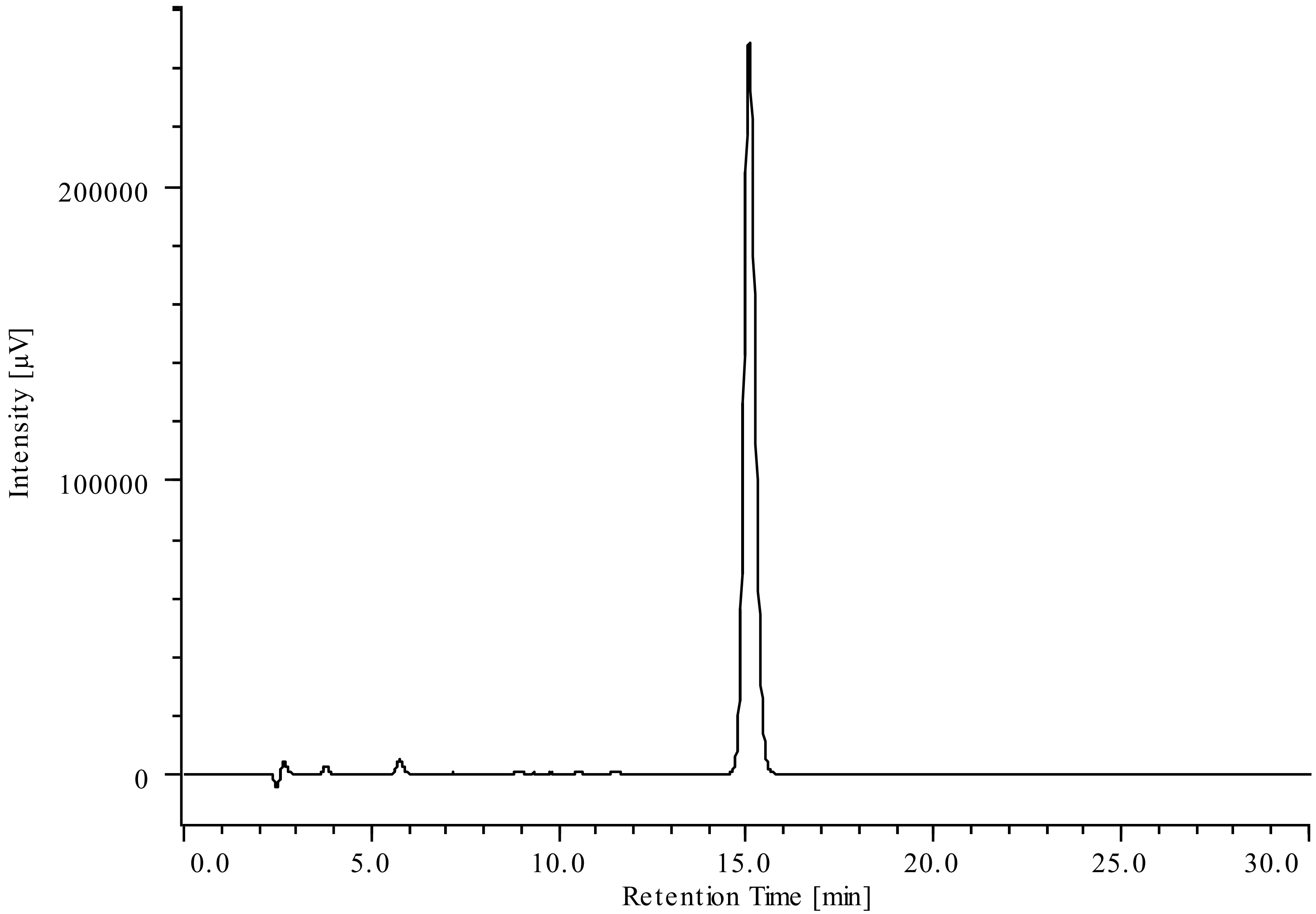

Figure S69. Compound **7**, HPLC chromatogram at its UV absorbance maximum (λ=300 nm). Purity: 95.4 %. Column: Kinetex®, 5 µm, Biphenyl, 100 Å, 250 x 4.6 mm (Phenomenex Inc.); Elution: water:CH_3CN (A:B) 40% B.

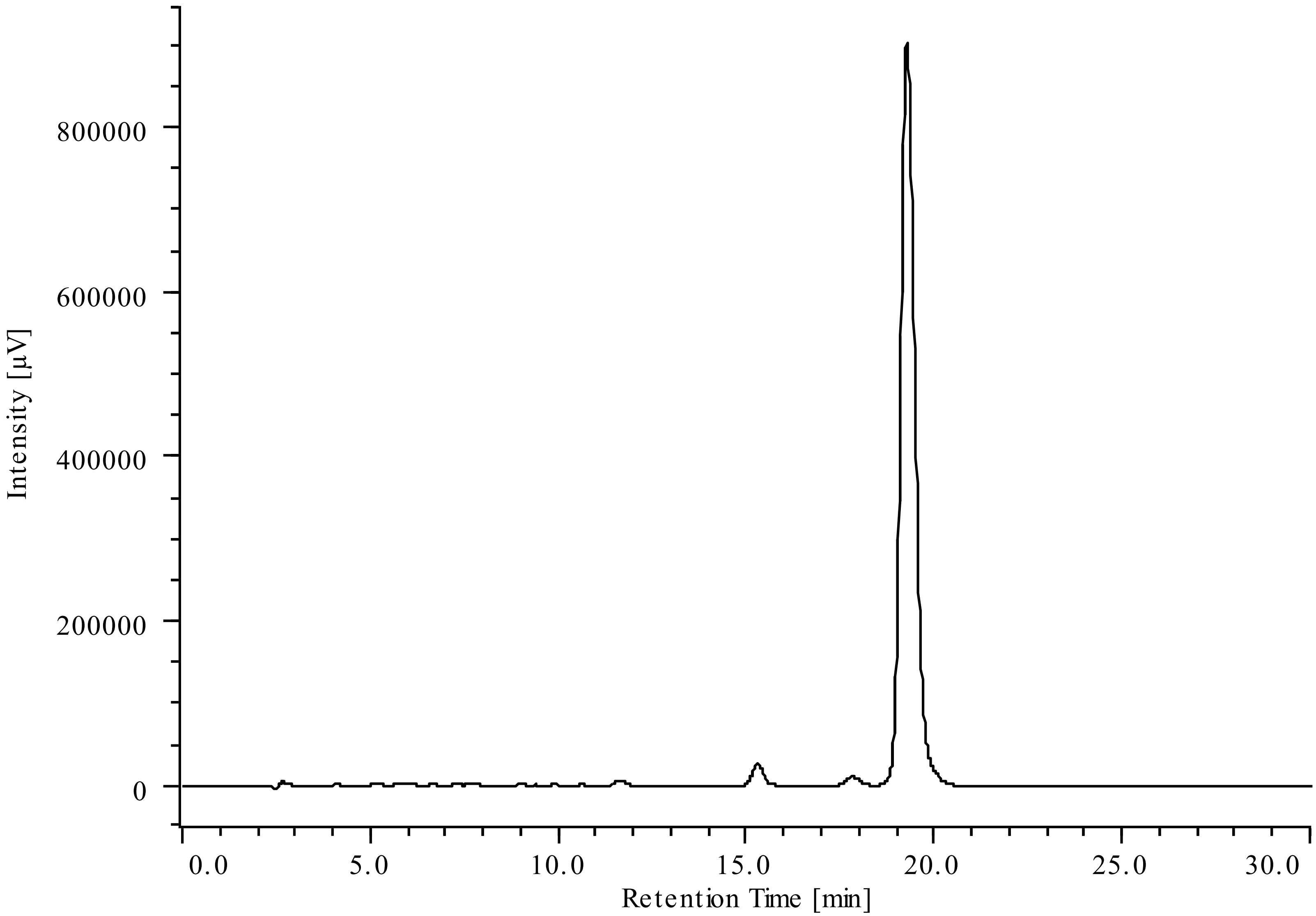

Figure S70. Compound **9**, HPLC chromatogram at its UV absorbance maximum (λ=242.6 nm). Purity: 98.0 %. Column: Kinetex®, 5 µm, XB-C18, 100 Å, 250 x 4.6 mm (Phenomenex Inc.); Elution: water:CH_3CN (A:B) 42% B.

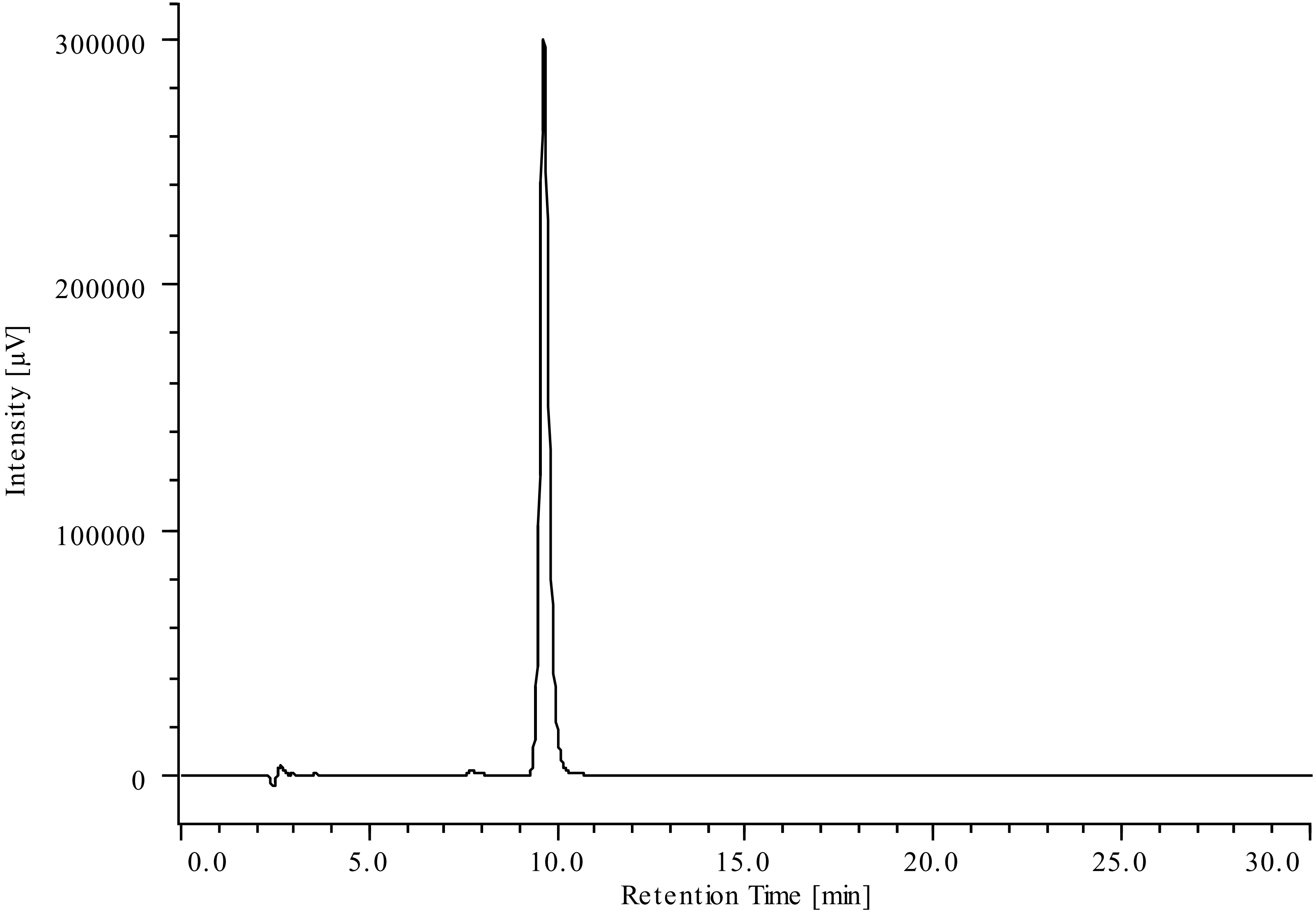

Figure S70. Compound **10**, HPLC chromatogram at its UV absorbance maximum (λ=300 nm). Purity: 99.5 %. Column: Kinetex®, 5 µm, XB-C18, 100 Å, 250 x 4.6 mm (Phenomenex Inc.); Elution: water:CH_3CN (A:B) 42% B.

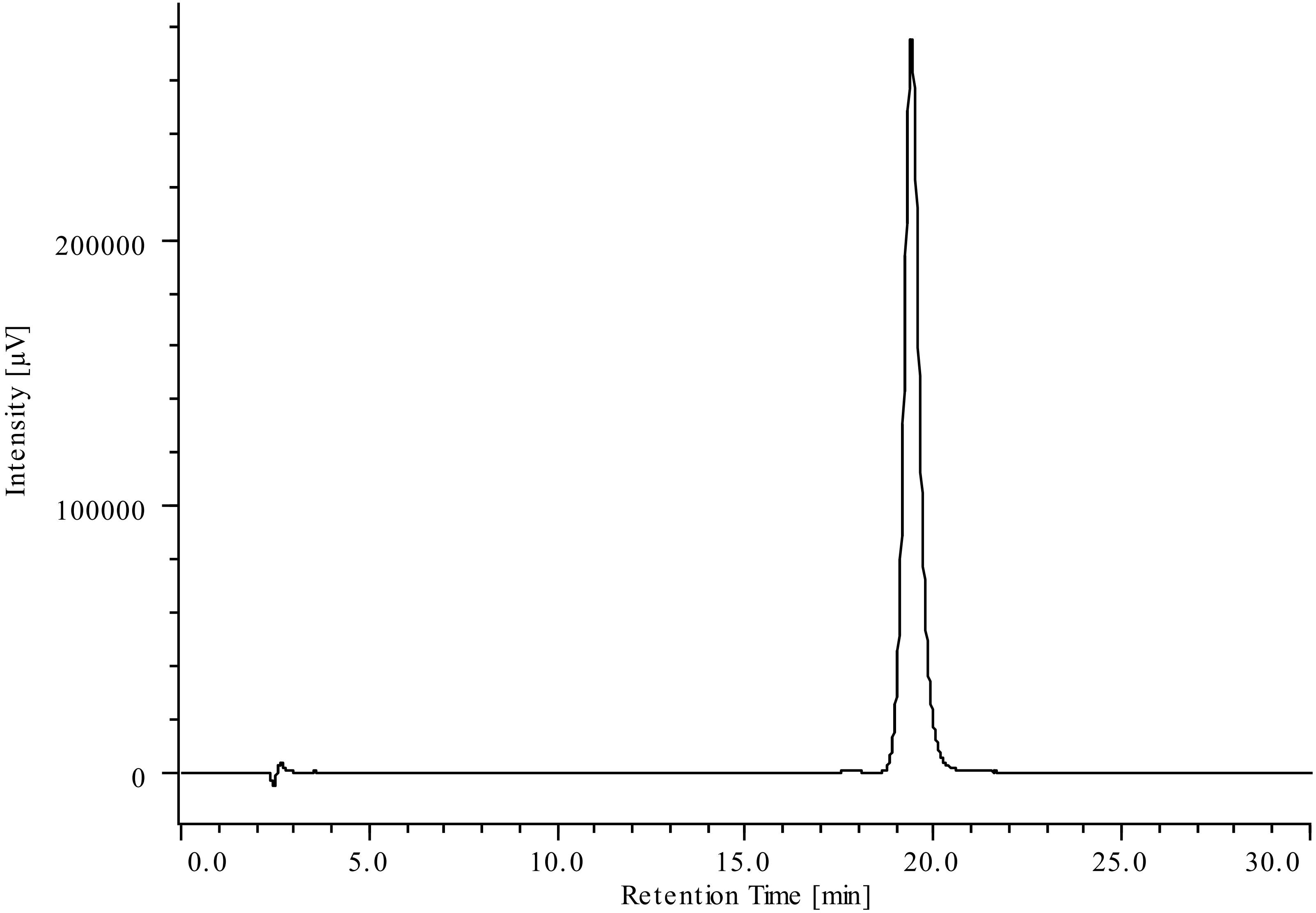

Figure S72. Compound **11**, HPLC chromatogram at its UV absorbance maximum (λ=300 nm). Purity: 99.3 %. Column: Kinetex®, 5 µm, XB-C18, 100 Å, 250 x 4.6 mm (Phenomenex Inc.); Elution: water:CH_3CN (A:B) 42% B.

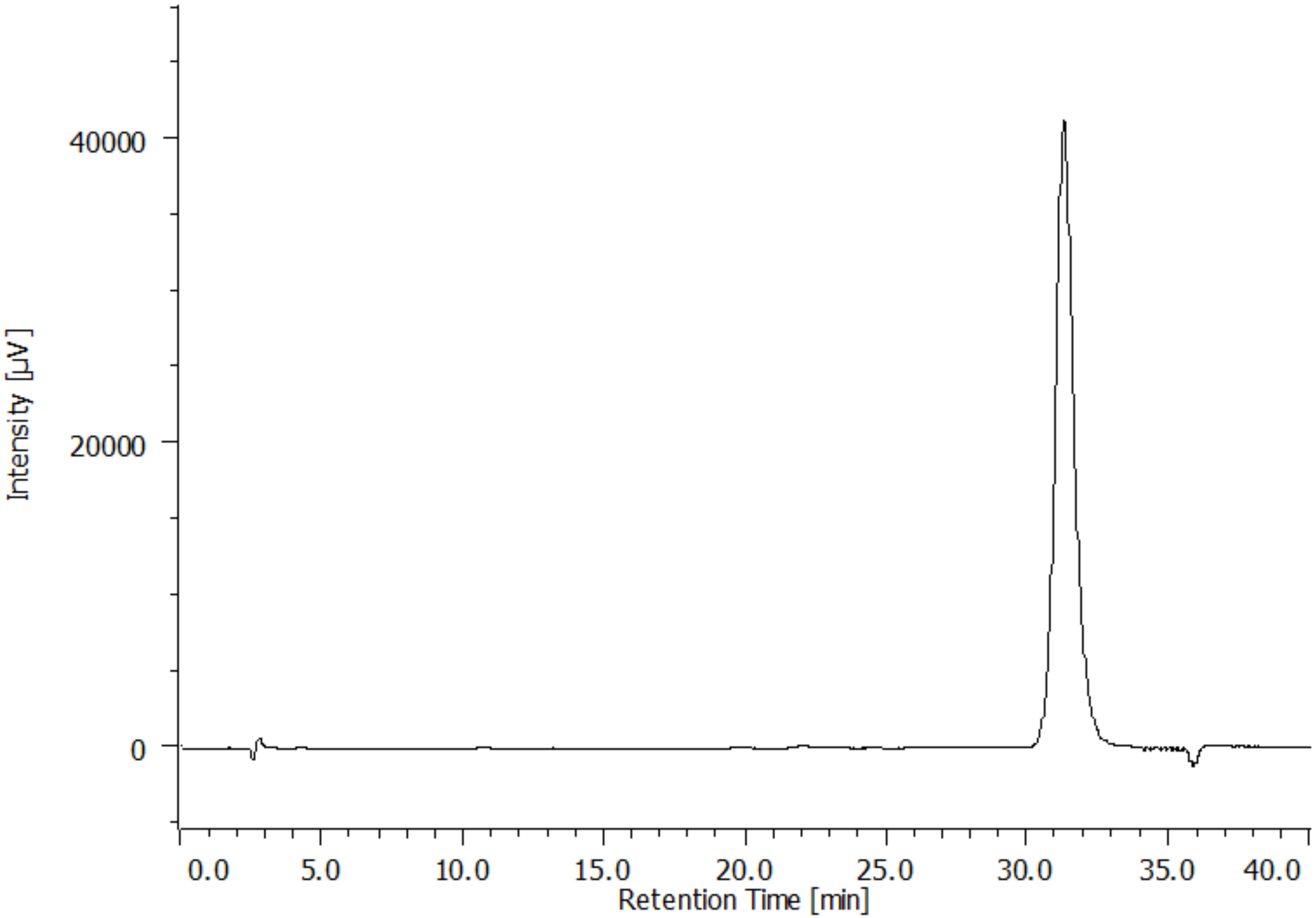

Figure S73. Compound **13**, HPLC chromatogram at its UV absorbance maximum (λ=300 nm). Purity: 98.4 %. Column: Kinetex®, 5 µm, XB-C18, 100 Å, 250 x 4.6 mm (Phenomenex Inc.); Elution: water:CH_3CN (A:B) 35% B.

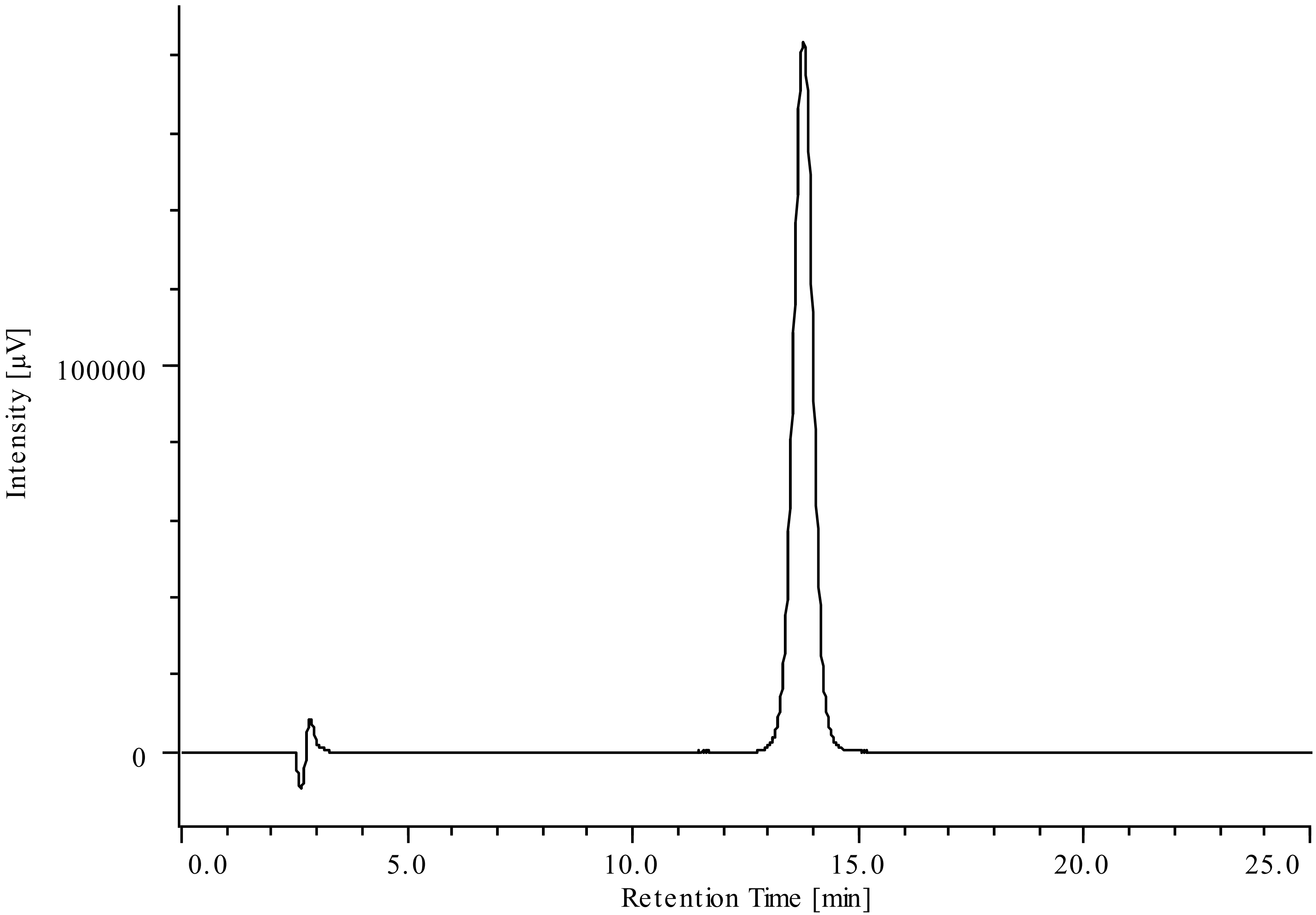

Figure S74. Compound **14**, HPLC chromatogram at its UV absorbance maximum (λ=240 nm). Purity: 97.7 %. Column: Kinetex®, 5 µm, XB-C18, 100 Å, 250 x 4.6 mm (Phenomenex Inc.); Elution: water:CH_3CN (A:B) 35% B.

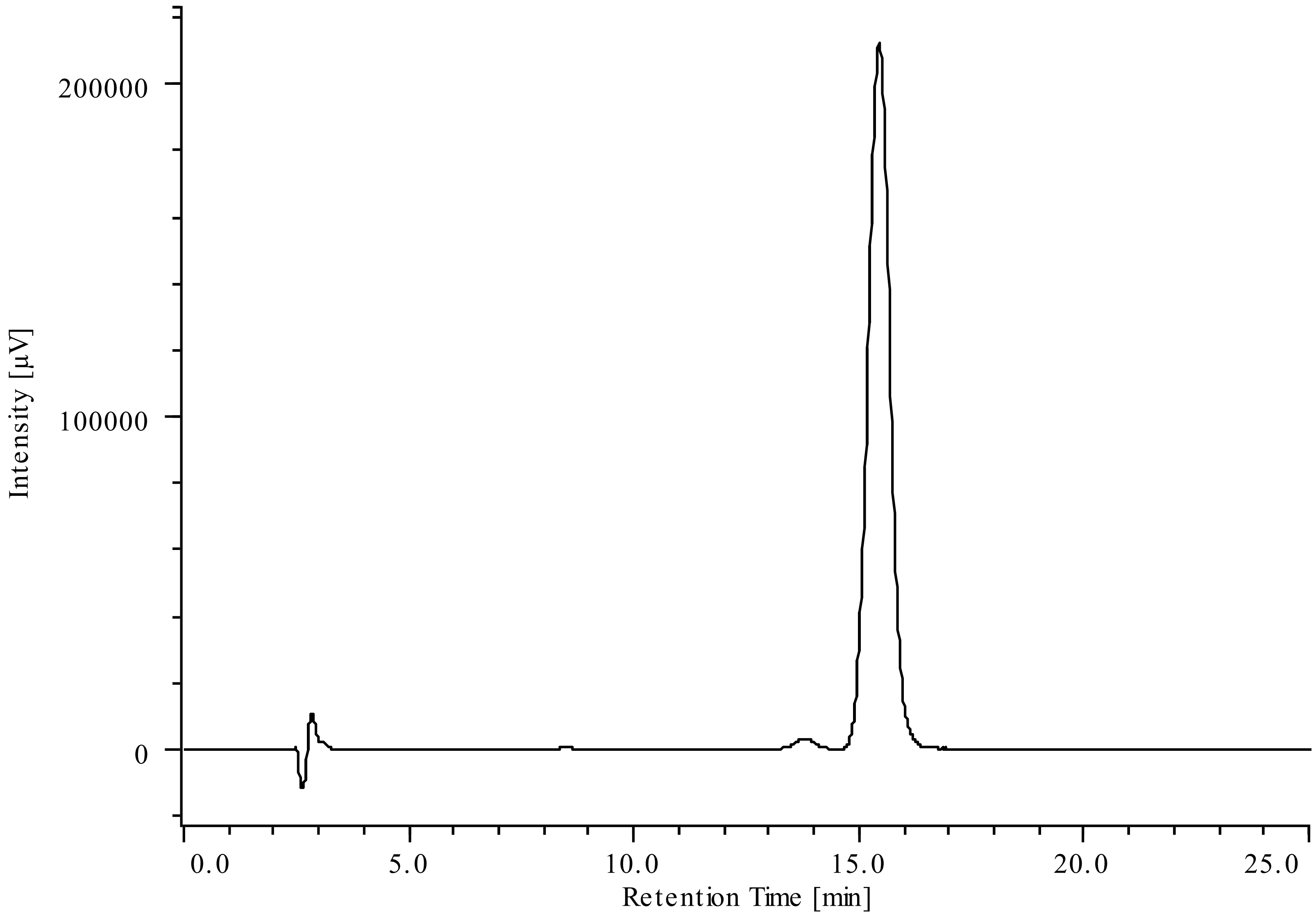

Figure S75. Compound **16**, HPLC chromatogram at its UV absorbance maximum (λ=220.8 nm). Purity: 98.4 %. Column: Kinetex®, 5 µm, XB-C18, 100 Å, 250 x 4.6 mm (Phenomenex Inc.); Elution: water:CH_3CN (A:B) 50% B.

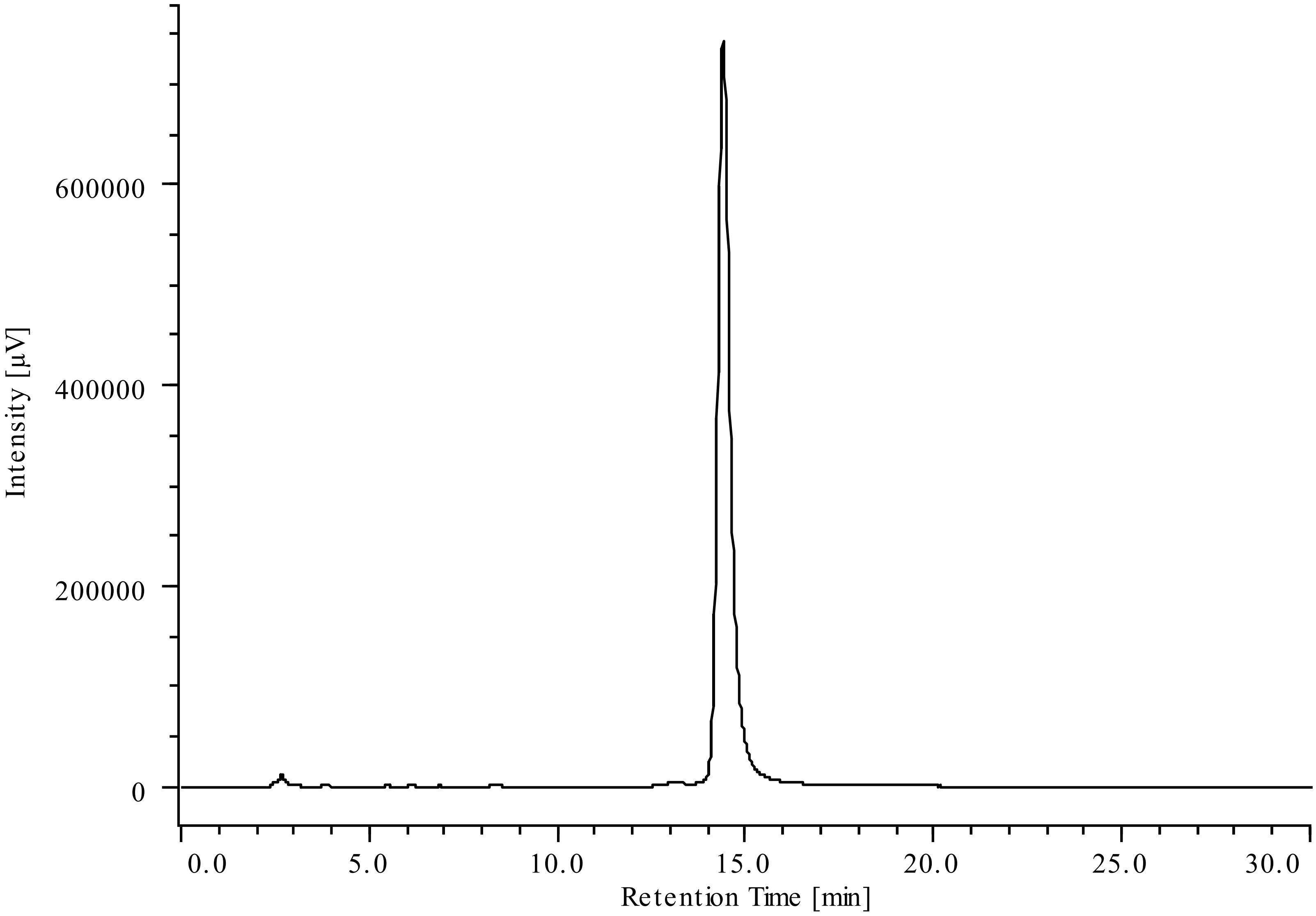

Figure S76. Compound **17**, HPLC chromatogram at its UV absorbance maximum (λ=358.5 nm). Purity: 98.1 %. Column: Kinetex®, 5 µm, XB-C18, 100 Å, 250 x 4.6 mm (Phenomenex Inc.); Elution: water:CH_3CN (A:B) 71% B.

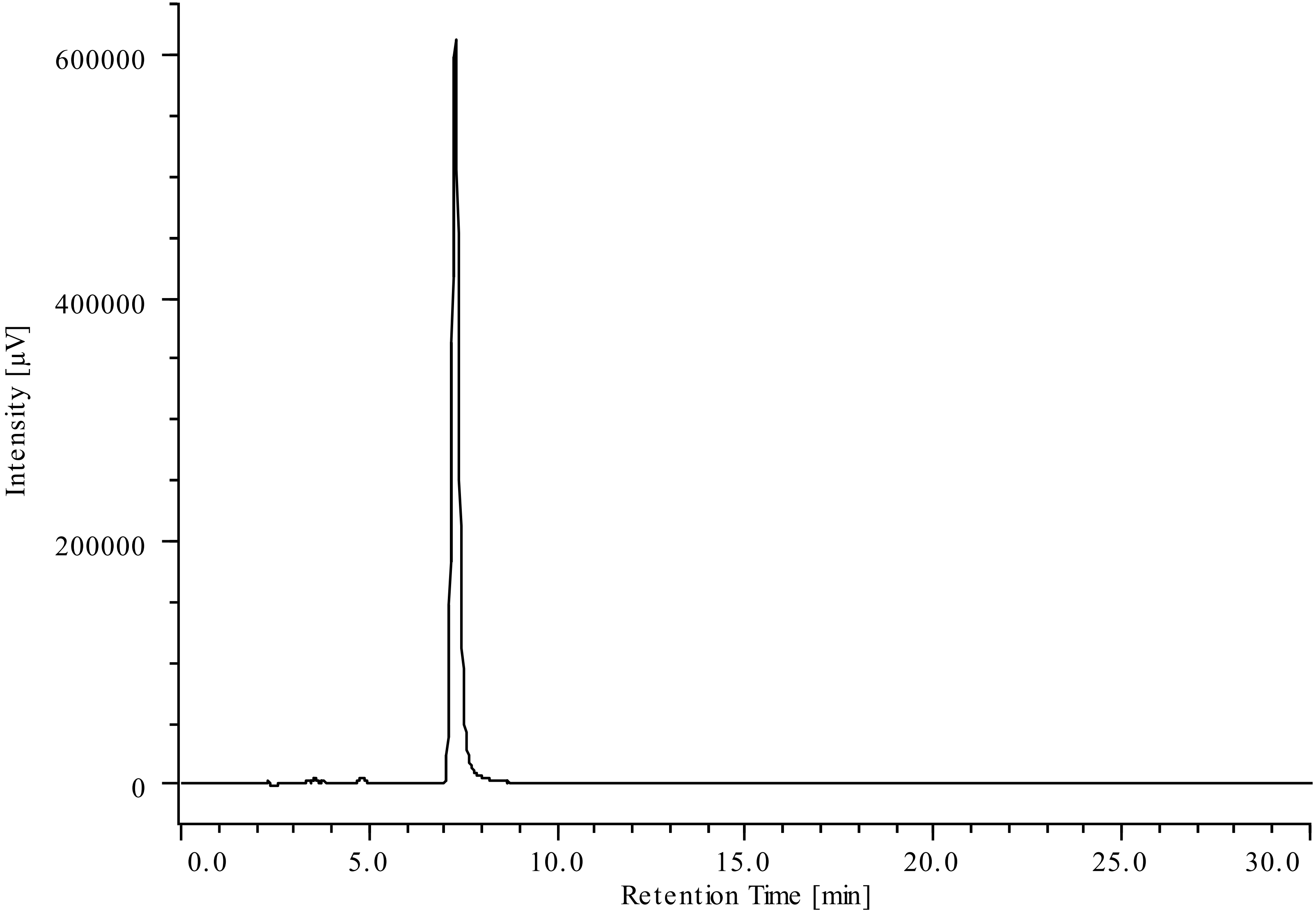

Supplement: Supplementary file 1 [file ijms-23-03447-s001.zip › ijms-1594754-supplementary.pdf]
